# Characterizing the impact of an exotic soybean line on elite cultivar development

**DOI:** 10.1371/journal.pone.0235434

**Published:** 2020-07-10

**Authors:** Benjamin B. Stewart-Brown, Justin N. Vaughn, Thomas E. Carter, Zenglu Li

**Affiliations:** 1 Department of Crop and Soil Sciences, Institute of Plant Breeding, Genetics and Genomics, University of Georgia, Athens, GA, United States of America; 2 Genomics and Bioinformatics Research Unit, USDA-ARS, Athens, GA, United States of America; 3 Soybean & Nitrogen Fixation Unit, USDA-ARS, Raleigh, NC, United States of America; University of Guelph, CANADA

## Abstract

The genetic diversity of North American soybean cultivars has been largely influenced by a small number of ancestors. High yielding breeding lines that possess exotic pedigrees have been developed, but identifying beneficial exotic alleles has been difficult as a result of complex interactions of yield alleles with genetic backgrounds and environments as well as the highly quantitative nature of yield. PI 416937 has been utilized in the development of many high yielding lines that have been entered into the USDA Southern States Uniform Tests over the past ~20 years. The primary goal of this research was to identify genomic regions under breeding selection from PI 416937 and introduce a methodology for identifying and potentially utilizing beneficial diversity from lines prevalent in the ancestry of elite cultivars. Utilizing SoySNP50K Infinium BeadChips, 52 high yielding PI 416937-derived lines as well as their parents were genotyped to identify PI 416937 alleles under breeding selection. Nine genomic regions across three chromosomes and 17 genomic regions across seven chromosomes were identified where PI 416937 alleles were under positive or negative selection. Minimal significant associations between PI 416937 alleles and yield were observed in replicated yield trials of five RIL populations, highlighting the difficulty of consistently detecting yield associations.

## Introduction

Generally, the genetic diversity present in improved plant cultivars only represents a small fraction of the total diversity present in the species from which the cultivar was derived [1]. This reduction in diversity is exemplified by soybean, wherein ~75% of North American cultivars released from 1947–1988 were derived from 17 ancestors and ~50% was derived from only six ancestral lines [2]. To further increase the rate of genetic gain in applied breeding beyond that now observed, it is imperative to mine global germplasm for beneficial alleles, such as novel alleles for pest-resistance [3]. These alleles can then be introgressed into cultivars via a conventional breeding or a marker-assisted selection approach [4–6]. Historically, the introgression of traits from wild progenitor germplasm as well as landraces has generally been limited to traits controlled by major genes. Such traits are easier to identify with confidence, less dependent on genetic background, and simpler to track during introgression. Though wild alleles for complex traits such as yield have been successfully identified using near-isogenic lines, these methods are highly resource intensive and often miss relevant alleles [7].

Ude et al. [8] indicated that Japanese cultivars provided a genetically distinct pool of material for improvement of North American soybean cultivars. Plant introduction (PI) 416937 is a Japanese landrace present in the pedigree of many elite lines/cultivars in the southeastern USA, most notably ‘Woodruff’ [9]. Woodruff has 25% genetic contribution from PI 416937 by pedigree and yielded 111, 122, and 111% of elite check, ‘Benning’ [10] in United States Department of Agriculture (USDA) Southern States Uniform Tests from 2003–2005 [11–13]. It has been reported that PI 416937 possesses several distinguishing characteristics including slow canopy wilting [14–17], proliferous fibrous roots [18–20], aluminum tolerance [21–22], altered vapor pressure deficit response, and other physiologically controlled drought-stress related traits [23–26]. ‘N7002’ (PI 647085) [27], ‘N8001’ (PI 647086) [28], and ‘USDA-N8002’ (PI 676972) [29] are additional cultivars which have >12.5% genetic contribution from PI 416937 by pedigree and have outperformed check cultivars in terms of yield within USDA Southern States Uniform Tests [11–13, 30–37]. Thus, unlike the common case in which exotic germplasm is used as a donor of a specific gene, the contributions of PI 416937 appear to be complex and its derived lines are examples of incorporating exotic germplasm in developing cultivars with increased yield and providing diversity for long-term genetic gain.

In this study, lines with known pedigree information related to PI 416937 were exploited using genome-wide single nucleotide polymorphism (SNP) marker data to track new exotic genomic regions that were selected for and against over approximately the last 20 years. The idea of exploiting breeding pedigrees to detect selected loci has been used previously in attempts to detect agronomically important loci in soybean [38–40]. Similar analysis has also been performed in peanut (*Arachis hypogaea*) [41]. The approach is akin to transmission disequilibrium tests (TDTs) pioneered in animal genetics [42]. Released cultivars are assumed to be the product of many stages of selection and, thus, alleles conferring superior fitness are expected to deviate from random (50%) transmission [42]. While original versions of the TDT have largely been performed in animal genetics, Jannink et al. [43] suggested that TDT can be adapted to self-pollinating crops by examining breeding lines and cultivars over decades to identify preferentially transmitted alleles hypothesized to be in linkage disequilibrium (LD) with favorable quantitative trait loci (QTL). Though the approach is theoretically very powerful, previous studies suffered from low marker density [38, 44] or gapped pedigrees that made rigorous statistical inference problematic [40]. Higher marker density allows for the confident inference of shared haplotypes in parent-offspring combinations, and, thus, enhances the ability to accurately define and count the number of crosses that test a locus for the influence of selection. In contrast with simple bi-parental mapping of yield, this deep pedigree approach has the advantage of differentiating genomic regions under breeding selection across multiple genetic backgrounds and environments and is robust to artifacts of segregation distortion that may be observed for a single parental combination.

The objective of this study was to identify haplotypes under breeder selection from PI 416937. Haplotypes have been defined as consecutive SNPs inherited from an individual progenitor genotype. This methodology has broad application to any breeding line which is prevalent within the ancestry of high yielding cultivars. We also discussed the implications of haplotypes identified via this methodology to target genomic regions lacking diversity within modern cultivars.

## Materials and methods

### Plant materials and population development

#### PI 416937-derived lines

High yielding PI 416937-derived lines were chosen based on inclusion in the USDA Southern States Uniform Tests, indicating that these lines had excellent yield potential based on previous replicated yield trials. PI 416937-derived lines spanned maturity groups (MGs) V-VIII. The combination of a PI 416937-derived line and its immediate parental lines was defined as a trio. A total of 52 trios were compiled and each member of the trio was genotyped for the pedigree analysis. For seven of these trios, both parents were derived from PI 416937. There were several lines that were derived from the same parent combinations. Thirteen of the 29 unique parental combinations had multiple progeny which were each considered as independent trios. These lines were developed by a modified single-seed descent (SSD) breeding method in which a single pod is selected from each plant in early selfing generations [45] so each line has a high likelihood of tracing to a unique F_2_ plant.

Lines chosen for the analysis were present in the USDA Southern States Uniform Test between 1994 and 2015, except for N93-110-6 which was bred for superior seed yield under drought-prone conditions [46]. Forty-four of the lines included in the analysis were bred within the USDA-Agricultural Research Service (ARS) Raleigh soybean breeding program, while the remaining eight were bred within the University of Georgia (UGA) soybean breeding program. The 52 trios were composed of a total of 76 independent lines. Genotypic data for each line was either procured from SoyBase (http://soybase.org) [47] or generated from seeds that were obtained from originating institutions.

Helium software [48] was used to create a pedigree tree of 232 lines and 387 relationships among these lines (S1 Fig). Six progeny [N7001, N90-7202, N90-7241, N93-110-6, N91-7254, and N93-1264] have PI 416937 as a direct parent. All genomic regions of PI 416937 inherited in other lines within this analysis originated from these six lines. N7001 had the strongest influence on our analysis with 12 direct progeny and 37 indirect progeny used in the pedigree analysis. Indirect progeny were defined as descendants of direct progeny. N90-7202 had the next strongest influence with six direct progeny and seven indirect progeny. N90-7241 had only two direct progeny used in the analysis but 11 indirect progeny. N91-7254 had one progeny and a single indirect progeny. N93-110-6 had one progeny while N93-1264 had zero progeny used in the analysis. While these progenitors narrow the scope of regions that can accumulate a large number of tests within the pedigree analysis, they do not inflate the significance of the regions found to be under selection since each trio is an independent test of a region.

#### Development of RIL populations

Five F_5_-derived recombinant inbred line (RIL) populations were developed with the intention of developing lines for germplasm enhancement or cultivar release. These RIL populations were used to evaluate PI 416937 alleles under selection for their effects on yield and investigate potential discrepancies in genomic regions associated with yield performance versus segregation distortion. These populations were inbred using the modified SSD method. Four of the RIL populations (RIL-1, 2, 3, 4) were comprised of 84 lines each while the fifth RIL population (RIL-5) had 150 lines (S1 Table). Each RIL population has PI 416937 in its pedigree. The term “trio” was used in this context to refer to an individual RIL and both parental lines.

### SNP genotyping

Twenty seeds from each line were planted in styrofoam cups in a UGA greenhouse. After 3 weeks, tissue from 15–20 plants of each line was bulked within 50-ml Falcon tubes (Fisher Scientific, Waltham, MA, US) and then lyophilized and ground into fine powder using a GenoGrinder (SPEX US, Metuchen, NJ, US). DNA was extracted by following the protocol from Keim et al. [49], with some modifications to improve purity of DNA. Key modifications included adding Edwards extraction buffer, NaCl, polyvinypyrrolidone, and Proteinase K to the CTAB 2ME buffer while performing a second 24:1 chloroform: isoamyl alcohol step to remove proteins and polysaccharides. An additional 75% ethanol wash was also performed.

For lines included in the pedigree analysis, SNP genotype data of 10 lines were obtained from SoyBase (http://soybase.org) [47] while the remaining 66 lines were genotyped either at Michigan State University or USDA-ARS, (Beltsville, MD) using SoySNP50K Infinium BeadChips [50]. The SNP loci that did not have a corresponding position in Gmax2.0 reference genome were excluded and a final set of 41,934 SNPs was utilized for analysis. The genotypic data for additional lines used for examining population structure were obtained from SoyBase as well (S2 Table). The five RIL populations were genotyped using SoySNP6K Infinium BeadChips at USDA-ARS (Beltsville, MD). The SoySNP6K Infinium BeadChip is an informative subset of the SoySNP50K Infinium BeadChip that makes genotyping a large number of individuals such as a RIL population more cost effective [51]. Physical positions of SNPs, originally based on reference genome Glyma.Wm82.a1 (Gmax1.01) [52] were converted to version Gmax2.0 for the analysis.

### Pedigree analysis utilizing genome-wide SNP data

The pedigrees of these PI 416937-derived lines were traced to the earliest discoverable antecedent lines (S1 Fig and S3 Table). Helium software was used to display the pedigree information [48]. Using SoySNP6K Infinium BeadChip data, the genomic contribution of PI 416937 versus major southern North American ancestors [53] to each high yielding PI 416937-derived progeny was measured. A chi-square test of given probabilities was performed in R, utilizing the chisq. test function within the ‘stats’ package [54] to examine how many lines deviated from expected percentage of genomic contribution from PI 416937. For each PI 416937-derived line, the observed percentage of genomic contribution from PI 416937 was based on molecular markers from the SoySNP50K Infinium BeadChip which allowed us to trace inheritance of alleles back to PI 416937 or other ancestral genotypes. This procedure for tracing inheritance is explained in more detail in the procedure for the pedigree analysis. The expected percentage of genomic contribution from PI 416937 was obtained from pedigree information.

To provide context of the genetic material used in this analysis compared to a representative panel of soybean germplasm, cladograms were created to examine population structure of PI 416937-derived lines compared to North American ancestral lines, as well as available public/private soybean germplasm/cultivars released before 2016 (S2 and S3 Figs). These materials included PI 416937, 95 PI 416937-derived lines, 32 selected southern lines, 38 North American ancestral lines, 464 public varieties, and 70 private varieties (S2 Table). Nineteen of the 95 PI 416937-derived lines were not included in the pedigree analysis because they did not contribute to a complete trio with genotypic data. These 19 lines were retained for the population structure analysis to give a more complete context of where PI 416937-derived lines reside compared to ancestral lines and modern varieties. The 32 southern lines were selected because they were the non-PI 416937 derived parental lines from the trios as well as common antecedents in southern pedigrees which were identified while tracing the pedigree history of the trios. The available public and private breeding lines were divided into groups based on decade of release, ranging from 1940’s to 2000’s. Cladograms were created from SoySNP50K Infinium BeadChip data via the neighbor joining clustering method in Tassel 5 [55] and plotted using ggtree [56] implemented in R [54]. This population structure analysis also assisted in clustering MG’s for a comparison between haplotypes under selection from PI 416937 and genomic regions of low diversity in modern soybean cultivars.

The pedigree analysis was performed on the PI 416937-derived lines and the five RIL populations. In this section, the PI 416937-derived lines and RILs are referred to as progeny. The first step was to identify which alleles were inherited from each parent by sequentially matching alleles of each progeny line to each parental line. To be consistent with the analysis of the high yielding PI 416937-derived lines, no genetic maps were made for the RIL populations. The goal was to calculate a match extension score, which determined the parent that had contributed a particular region of the inbred progeny haplotype. Each matched allele was worth a point. If a locus in either the parent or progeny line was heterozygous, this was called as half of a point. Missing values were worth zero points. This matching continued until the matching was broken by an opposite allele being present in the parent. Then for a given matched segment, a score was calculated based upon the quality of the match and the match with the higher score was called the parent of origin. The parental match had to be at least two markers longer in one parent to be considered definitively one parent versus the other. In theory, a genomic region called for one parent could be from a shorter consecutive match because the longer match to the other parent had an excess of heterozygous loci or missing data points. This strategy was similar to the haplotype matching strategy implemented in Vaughn and Li [53]. If a given region had the same score in the two parents or the match was not two markers longer in one parent, the region could not definitively be called for either parent and it was called as ambiguous. Once the parental regions were identified, the origin of each region was identified with relation to PI 416937 as well as to the predominant ancestral lines of North American southern elite material according to Vaughn and Li [53]. North American southern ancestors were chosen versus all North American ancestors because the genotypes used in the analysis were predominantly comprised of southern germplasm and this also resulted in less ambiguity in identifying ancestor sources for the genomic regions. In this way, genomic origins of these progeny lines could be traced back to their parental sources and then to their original ancestral sources. The analysis focused on regions in which one parent contained an allele from PI 416937 at a particular locus and the other parent did not. Given a sufficient number of such scenarios, the probability that a locus was neutral could be evaluated statistically utilizing a two-sided exact binomial test. Even if a region could not be definitively assigned to a southern ancestor, it was still considered a test if the allele could be determined to not be from PI 416937 (assuming the alternative allele was from PI 416937).

To obtain *P*-values for alleles under selection, a two-sided exact binomial test [57] was performed in R, utilizing the binom. test function within the ‘stats’ package [54]. If we assume there is a 50 percent chance of inheritance of an allele from a parental genotype and a binomial distribution of outcomes when no selection pressure is imposed, a two-sided exact binomial test was appropriate to asses statistical deviation from a null hypothesis of no selection pressure. The number of successes was entered as the number of times that the PI 416937 allele was inherited. The number of trials was entered as the number of trios in which the PI 416937 allele was tested in the parents. With no selection, a success rate of 0.5 was expected. A haplotype previously associated with seed yield from PI 416937 referred to as Yld1 [58] was utilized to set the threshold for significant evidence of selection. Using 66 F_4:6_ RILs derived from N07-14221 × ‘Clifford’ (PI 596414) that were segregating for the PI 416937 allele at the Yld1 locus, Eickholt [58] had previously shown that the PI 416937 allele resulted in a significant (*P* < 0.05) increase in seed yield of 76 kg ha^-1^. This was an average over three locations in 2015. Within our pedigree analysis, the Yld1 haplotype from PI 416937 showed a strong signal for selection relative to other PI 416937-derived haplotypes, providing additional support for utilizing the *P*-value of this genomic region as a statistical threshold. As for the RIL populations, genomic regions appeared to be under significantly greater selection pressure so they were determined to be significant based on a multiple test corrected threshold. The significance threshold was set at an alpha of 0.05 and a multiple test correction was performed based upon linkage disequilibrium (LD) between markers. To adjust the significance threshold for LD, the number of markers with an R-squared less than 0.8 was calculated and these markers were labeled as tag SNPs. Tag SNPs were identified using the tagger function in Haploview [59–60]. The *P*-value threshold of 0.05 was then divided by the number of tag SNPs to obtain the significance threshold. The number of tag SNPs ranged from 423 to 616 depending upon the population.

For both the PI 416937-derived line and RIL pedigree analyses, regions under selection were defined as any run of consecutive markers surpassing our chosen statistical significance threshold. There were several situations in which markers showed varying levels of significance within a run of consecutive markers. The markers with the highest levels of significance within these runs of consecutive markers were referred to as peak regions. Markers in our results that were not tested in at least 10 trios were excluded.

### Yield trials and analysis of RILs

Five RIL populations with PI 416937 present in their pedigrees were yield-tested to validate the genomic regions identified as under selection from the PI 416937-derived pedigree analysis. Yield evaluations of RIL-1 were conducted in 2014 and 2016 at two locations in Georgia (Athens and Plains). RIL-2 was evaluated in the same years but only in Athens because of lack of seed. Yield evaluations for RIL-3 and RIL-4 were conducted in 2015 and 2016 at the same locations as RIL-1. Each population consisted of 84 F_5:6_ RILs, divided by maturity into two subsets of 42 RILs each for yield testing. Two elite cultivars were included as checks in each subset. These tests were conducted in a randomized complete block design with two replications per location in 2014 and 2015 and three replications per location in 2016. For both locations, lines were planted in two-row plots, 4.9 m long and 76 cm apart. The plots were end-trimmed to 3.7 m at R5-6 stage and harvested for yield. Yield data was normalized on a 13% moisture basis. Maturity was recorded as days to maturity from September 1^st^.

Yield evaluations of RIL-5 took place across five environments in 2014 and 2015 in Georgia and Louisiana. These tests were also conducted in a randomized complete block design with two replications per environment. This population consisted of the 150 F_5_-derived RILs which were separated into three subsets of 50 RILs each based upon maturity. Two elite check cultivars were included twice in each subset. For Georgia environments (Athens and Plains), plots were the same as RIL1-4. In Bossier City, LA, RILs were planted in two-row plots that were 4.9 m long and 102 cm apart and both rows were harvested for yield. Maturity notes for all five RIL populations were recorded in Athens, GA on all replications in each year.

These RIL populations were genotyped with the SoySNP6K Infinium BeadChip and ancestral genomic contribution was determined using the same methodology as for the PI 416937-derived lines. PI 416937 regions segregating within these RIL populations were identified. Several PI 416937 regions which were found to be under selection in the pedigree analysis of PI 416937-derived lines appeared to be segregating in the RIL populations, so these RIL populations served as a potential source of validation for the effects of these regions on yield. For each RIL population, mixed models were used to estimate yield across environments with the PI 416937-derived region under selection as a fixed effect and environment, genomic region × environment, and subset within environment as random effects. The Tukey HSD multiple means comparison test was performed on each segregating region for seed yield.

A similar analysis was performed for maturity to examine if there were significant differences in maturity associated with genomic regions from PI 416937 within the RIL populations. For maturity, the mixed model had to be simplified by removing the interaction term in order to detect statistical differences. Statistical analyses for all previously mentioned mixed model analyses were performed using JMP^®^ Pro 13.0.0 [61].

## Results

### PI 416937 genomic contribution within high yielding derived lines

A chi-square test was performed to examine which lines were significantly different in terms of percentage of PI 416937 and southern ancestors estimated by markers versus what was expected by pedigree (Table 1). Regions which were ambiguous were not included in the analysis so the percentage of the genome inherited from PI 416937 and southern ancestors was normalized for each line.

**Table 1:** Trios used in PI 416937 pedigree analysis and chi-square test to examine deviation of expected versus observed genomic contribution of PI 416937 and southern ancestors in each high yielding PI 416937-derived line. Ordered by % PI 416937 by markers.

Name^a^	MG	% PI 416937 by pedigree	% PI 416937 by markers	% Southern ancestor by pedigree	% Southern ancestor by markers	% Ambiguous by markers	Female parent	Male parent	Year(s) entered in USDA Uniform Test
N05-7375***	VI	25.0	6.8	75.0	84.5	8.7	N7002^b^	N98-7265	2009, 2010, 2011
G08-3279RR	VIII	12.5	8.0	87.5	80.3	11.6	Woodruff^b^	G03-952RR	2011, 2012, 2013, 2014
N06-7564	VII	12.5	8.1	87.5	80.2	11.6	NC-Roy	N8001^b^	2008, 2009, 2010
N07-14221	V	12.5	10.7	87.5	83.5	5.7	N7002^b^	Clifford	2012
G08-3282RR	VIII	12.5	11.1	87.5	76.1	12.7	Woodruff^b^	G03-952RR	2011, 2012
G10-3896RR	VIII	12.5	11.9	87.5	82.2	5.9	G03-825RR	G00-3213^b^	2013
N05-7452	VII	12.5	12.2	87.5	78.2	9.5	N7002^b^	5601T	2007, 2008, 2009, 2010, 2011
N05-7353***	VI	25.0	12.2	75.0	83.0	4.8	N7002^b^	N98-7265	2009, 2010, 2011
N07-15546	VI	12.5	12.4	87.5	79.4	8.1	N7002^b^	PI 221717	2012
N06-7535	VII	12.5	12.5	87.5	77.3	10.1	NC-Roy	N8001^b^	2009, 2010
N05-7396**	VII	25.0	13.0	75.0	76.3	10.6	N7002^b^	N98-7265	2007, 2008 2009, 2010
N8002**	VIII	25.0	13.5	75.0	80.7	5.7	N7002^b^	N98-7265	2007, 2008 2009, 2010, 2011, 2012, 2013, 2014, 2015
N01-11118*	VII	25.0	13.8	75.0	73.6	12.6	NTCPR94-5157	N96-7031^b^	2005
N07-15529	VII	12.5	14.0	87.5	73.0	12.9	N7002^b^	PI 221717	2014, 2015
N01-11777**	VII	25.0	14.5	75.0	78.9	6.5	Graham	N96-7031^b^	2004, 2005, 2006, 2007, 2008, 2009
G08-2869RR	VIII	12.5	14.9	87.5	74.6	10.5	Woodruff^b^	G03-364RR	2011, 2012
G10-3833RR	VII	12.5	15.3	87.5	75.0	9.7	G03-825RR	G00-3213^b^	2013, 2014
N06-7280*	VI	25.0	15.5	75.0	76.6	7.9	N98-7265	N7002^b^	2009, 2010
N8001	VIII	25.0	16.7	75.0	73.2	10.1	N7001^b^	Cook	2000, 2001, 2002, 2003, 2004, 2005, 2006, 2007
N07-14182	VI	12.5	16.8	87.5	73.9	9.2	N7002^b^	Clifford	2011, 2012
G00-3083	VIII	25.0	17.0	75.0	76.3	6.7	N7001^b^	Benning	2003
N05-7380	VII	25.0	18.0	75.0	73.7	8.2	N7002^b^	N98-7265	2012
N01-11136	VII	25.0	18.9	75.0	70.7	10.3	NTCPR94-5157	N96-7031^b^	2004, 2005, 2006, 2007, 2008, 2009
N01-11491	VII	25.0	19.7	75.0	64.6	15.6	NTCPR94-5157	N96-7031^b^	2005, 2006, 2007
N01-11771	VII	25.0	21.1	75.0	71.3	7.5	Graham	N96-7031^b^	2006, 2007, 2008, 2009
N05-7281	VII	25.0	21.3	75.0	71.7	6.9	N96-6809^b^	N98-7265	2007, 2008, 2009, 2010, 2011
N01-11424	VIII	25.0	21.4	75.0	69.0	9.5	NTCPR94-5157	N96-6767^b^	2006, 2007, 2008
N01-11884	VII	25.0	22.6	75.0	70.1	7.2	Graham	N96-6767^b^	2006, 2007
Woodruff	VII	25.0	23.2	75.0	70.3	6.4	N7001^b^	Boggs	2003, 2004, 2005, 2006
N05-7462	VII	25.0	23.7	75.0	69.6	6.6	5601T	N96-6809^b^	2007, 2008, 2009, 2010, 2011
N01-11791	VII	25.0	23.8	75.0	70.6	5.6	Graham	N96-7031^b^	2005
N01-11832	VIII	25.0	25.4	75.0	69.0	5.5	Graham	N96-7031^b^	2005
TCWN23-507	VI	25.0	25.6	75.0	60.7	13.7	N77-114	N96-6809^b^	2007
N06-7187	VIII	25.0	25.6	75.0	69.6	4.7	N98-7265	N93-110-6^b^	2009, 2010, 2011
N05-316	VI	25.0	25.6	75.0	65.2	9.1	NC-Roy	N96-6752^b^	2013, 2014, 2015
N7002	VII	25.0	25.9	75.0	68.6	5.4	N7001^b^	Cook	2000, 2001, 2003, 2004, 2005, 2006, 2007
G07-3557RR***	VIII	12.5	26.2	87.5	64.1	9.6	G00-3213^b^	P97M50	2010, 2011, 2012
N09-12414***	VII	12.5	27.5	87.5	60.6	11.8	N7002^b^	Misuzu Daizu	2011, 2012
N09-12441***	VII	12.5	29.8	87.5	58.3	11.8	N7002^b^	Misuzu Daizu	2013
N93-1264**	V	50.0	30.9	50.0	59.0	10.0	Brim	PI 416937	1998
N09-12455***	VII	12.5	33.7	87.5	52.9	13.3	N7002^b^	Misuzu Daizu	2013, 2014
N99-8141*	V	25.0	34.3	75.0	60.5	5.1	N7001^b^	Graham	2002, 2003
N04-8947	VII	50.0	40.2	50.0	55.3	4.4	N96-6894^b^	N97-9812^b^	2008, 2009, 2010
N05-7229	VII	50.0	41.8	50.0	42.4	15.7	N96-6809^b^	N96-7031^b^	2007
N93-110-6	VI	50.0	42.0	50.0	55.3	2.6	Young	PI 416937^b^	Devi et al., 2014^c^
N05-7260	VII	50.0	43.6	50.0	51.1	5.2	N96-6809^b^	N96-7031^b^	2007, 2008, 2009, 2010
N90-7202	VII	50.0	46.7	50.0	49.3	4.0	N77-114	PI 416937^b^	1994
N7001	VII	50.0	48.7	50.0	47.6	3.7	N77-114	PI 416937^b^	1994, 1995, 1996, 1997
N96-6751	VII	50.0	49.7	50.0	42.6	7.7	N90-7202^b^	N7001^b^	1998
N96-6752	VIII	50.0	49.9	50.0	45.6	4.4	N90-7202^b^	N7001^b^	1999, 2000, 2001, 2002, 2003, 2004, 2005
N96-6809	VII	50.0	50.5	50.0	44.6	4.8	N90-7202^b^	N7001^b^	1998, 1999, 2000, 2001, 2002
N96-6755	VI	50.0	51.0	50.0	43.3	5.6	N90-7202^b^	N7001^b^	2001, 2002, 2003

*, **, and *** denote significant difference in estimated inheritance by pedigree versus measured inheritance by marker according to chi-square test for given probabilities at an alpha of 0.05, 0.01, and 0.001, respectively.

^a^ Prefix G and N in the name denote the lines developed at the University of Georgia and at USDA Raleigh, respectively. Woodruff was developed at the University of Georgia while TCWN23-507 was developed at USDA Raleigh.

^b^ Parent with PI 416937 in pedigree.

^c^ Line not evaluated in a USDA Uniform Test, but was bred for superior seed yield under drought-prone condition.

By pedigree, percentage of PI 416937 genome ranged from 12.5 to 50.0% in the high yielding progeny (Table 1). Using marker data, percentage of PI 416937 genome ranged from 6.8 to 51.0% across all trios. N09-12455 contained 2.7-fold more PI 416937 genome by marker than predicted by pedigree (33.7% actual versus 12.5% predicted). N93-1264 contained 1.6-fold less PI 416937 genome by marker than predicted by pedigree (30.9% actual versus 50.0% predicted). These were the largest discrepancies observed in predicted versus measured. N96-6755 contained the largest portion of the PI 416937 genome with 51.0% based upon marker data which was not different (*P* < 0.05) from the 50% that was predicted. N05-7375 contained the smallest portion of the PI 416937 genome with 6.8% based upon marker data. This was significantly different (*P* < 0.05) from the 25% that was predicted.

Thirteen of the 52 high yielding lines derived from PI 416937 had significantly different ratios of PI 416937 to southern ancestor from what was predicted (*P* < 0.05). Six lines contained significantly more PI 416937 genome and seven contained significantly less PI 416937 genome than predicted (*P* < 0.05). There appeared to be no selective advantage for inheriting a larger portion of the PI 416937 genome.

### Discovery of genomic regions under both positive and negative selection

**Fig 1:**
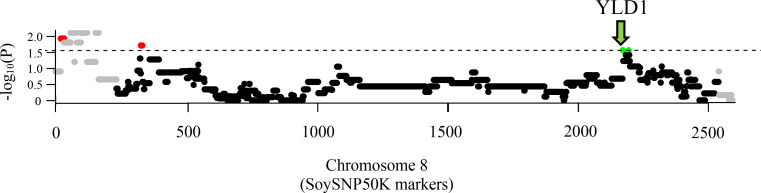
PI 416937 whole-genome based pedigree analysis of chromosome 8. The top section indicates genomic regions across chromosome 8 under significant positive (green) versus negative selection (red). The statistical threshold was set at a–log_10_ *P*-value of 1.56 (Yld1). The x-axis displays marker positions from SoySNP50K Infinium BeadChips. Gray indicates a locus had less than 10 trios testing a PI 416937 allele against an alternative allele. Black indicates a locus had 10 trios or more but fell below our significance threshold.

Although there was no selective advantage for PI 416937 in terms of overall genome contribution, it was hypothesized that there was commonality between the particular genomic regions from PI 416937, some beneficial, some deleterious, inherited to the high yielding progeny. Using 52 trios, genomic regions from PI 416937 were identified under both positive and negative selection. The structure of the pedigree analysis allowed for direct definition of a null model and evaluation of *P*-values. The statistical significance of a PI 416937 region with previous evidence of an association with yield was set as an empirical threshold. This region (Yld1) was identified as our eighth most significant region in terms of positive selection as it was tested in 41 trios and inherited in 28 high yielding progeny (*P* = 2.75 × 10^−2^) (Fig 1, additional files for other chromosomes included in S1 File). Any markers or haplotypes that met this level of statistical significance or greater were determined to be regions with evidence of selection for or against. In total, nine genomic regions under positive selection and 17 genomic regions under negative selection from PI 416937 were identified across seven chromosomes (Table 2 and S4 Fig). The full results from the PI 416937 pedigree analysis are presented in S2 File.

**Table 2:** Summary of PI 416937 pedigree analysis.

Genomic region	Chr	Direction of selection	Physical start position (bp)	Physical stop position (bp)	SNPs	Trios tested	No. of times inherited	*P*-value
1	5	Negative	488551	523725	3	36	10	0.011
			558763	-	1	41	11	0.004
			562322	593685	3	39	10	0.003
			595812	-	1	41	11	0.004
2	5	Negative	1496131	-	1	38	11	0.014
3	8	Negative	1017668	1373179	13	11	1	0.012
4	8	Negative	5750622	5968621	6	19	4	0.019
5	8	Positive	41792467	41796167	2	41	28	0.028
6	8	Positive	42070881	-	1	41	28	0.028
7	9	Negative	45849012	45885099	3	10	1	0.022
			45913326	45941083	2	11	1	0.012
8	9	Negative	46044100	46082968	3	11	1	0.012
9	12	Negative	14014843	14384675	7	20	4	0.012
10	12	Negative	17662053	20410513	34	28	7	0.013
			20474981	21673448	15	27	7	0.019
11	13	Positive	26986028	27085556	14	26	21	0.003
12	13	Negative	27971359	-	1	20	3	0.003
			27979190	27991927	3	19	3	0.004
13	13	Negative	28203902	28286301	19	22	5	0.017
14	13	Negative	28346050	28351526	2	20	3	0.003
15	13	Negative	28475417	29111990	83	22	5	0.017
16	13	Negative	29128801	29533624	48	22	5	0.017
17	13	Negative	30683322	30765585	5	10	0	0.002
18	13	Positive	36166900	36175098	2	32	23	0.020
19	13	Positive	37465322	37527009	18	33	24	0.014
			37529648	37550786	2	35	26	0.006
			37551787	37553062	3	41	29	0.012
20	16	Negative	6871009	6896309	6	12	1	0.006
			6914854	6948002	6	15	2	0.007
21	17	Positive	796471	1309414	26	17	14	0.013
22	17	Positive	2202411	-	1	26	20	0.009
23	17	Positive	2246668	2408798	13	26	20	0.009
			2409261	2418484	3	26	21	0.003
			2419489	2438284	7	26	20	0.009
			2452744	2453238	2	10	9	0.022
24	17	Positive	2510699	3496006	76	26	21	0.003
25	17	Negative	38511430	38757540	26	13	2	0.023
26	19	Negative	1743312	3274996	111	13	2	0.023

Regions under positive selection ranged from a single marker to 76 consecutive markers in length. Regions were found on Chrs 8 (2 regions), 13 (3 regions), and 17 (4 regions), respectively (Table 2 and S4 Fig). The physical distance of the largest region under positive selection was 985,307 bp on Chr 17. There were three other genomic regions across Chrs 13 and 17 with the greatest evidence of positive selection (*P =* 2.49 × 10^−3^). The first region was a 99,528 bp region on Chr 13. The second region was a 9223 bp region on Chr 17 located within a larger significant region of 206,570 bp. The third genomic region with the greatest evidence of positive selection was in an interval of 985,307 bp and also on Chr 17.

Regions identified under negative selection ranged from a single marker to 137 consecutive markers in length. Regions were found on Chrs 5 (2 regions), 8 (2 regions), 9 (2 regions), 12 (2 regions), 13 (6 regions), 16 (1 region), 17 (1 region), and 19 (1 region), respectively (Table 2 and S4 Fig). The physical distance of the largest region under negative selection was 4,011,395 bp on Chr 12. A peak region was identified with higher significance within this larger region, which was 2,748,460 bp long. The genomic region with the greatest evidence of negative selection (*P* = 1.95 × 10^−3^) was 82,263 bp long on Chr 13.

### Evaluation of PI 416937-derived regions under selection using RIL populations

Using five RIL populations that had PI 416937 in their pedigrees, we tested for a yield advantage associated with any of the genomic regions under selection. A standard QTL mapping approach was not employed because of the greater statistical precision derived from pre-planned orthogonal comparisons [62] resulting from the pedigree analysis of genomic regions derived from PI 416937.

Seven regions under positive selection identified from the pedigree analysis of PI 416937-derived lines were segregating in at least one of these RIL populations. Regions 5 and 6 on Chr 8 were segregating in three RIL populations (RIL-1, 3, and 5); region 19 on Chr 13 in four RIL populations (RIL-1, 2, 4, and 5); and regions 21, 22, 23, and 24 on Chr 17 in three RIL populations (RIL-1, 2, and 5). Yield analysis was performed within individual populations across environments and the only significant yield effect was that the PI 416937 haplotype for region 19 had a positive yield effect of 27 kg ha^-1^ (*P* < 0.05) in RIL-4 and matured 0.8 days later. This PI 416937 haplotype resulted in a larger positive yield effect in RIL-2 (41 kg ha^-1^) and RIL-5 (76 kg ha^-1^) though these effects were not significant (*P* < 0.05). There was also a negligible difference in days to maturity between haplotypes in RIL-2 and RIL-5 as RILs with the PI 416937 haplotype matured 0.4 days earlier and 0.1 days later. The PI 416937 haplotype had a negative effect of 105 kg ha^-1^ versus the alternative haplotype in RIL-1 but it was not significant (*P* < 0.05). Two of the seven segregating regions fluctuated between having a positive and negative effect and the remaining five had negative effects across segregating populations, although the effects were not significant (*P* < 0.05).

The same pedigree analysis performed on the PI 416937-derived lines was also performed within each of the five RIL populations to determine if there were any regions under positive or negative selection within these RIL populations. These RIL populations were developed for breeding purposes and underwent modified SSD for three generations followed by single plant and then single plant-row selection via visual evaluation before being grown in advanced yield trials. Thus, regions found to be under significant positive or negative selection would be regions subject to segregation distortion. These regions did not arise from extensive yield trials as with regions from the PI 416937 pedigree analysis performed above. Genomic regions under selection pressure in these early rounds of selection for these five RIL populations were compared to regions in the PI 416937-derived line pedigree analysis for potential overlap. If so, this would be evidence that these regions were selected based upon obvious vigor that is evident visually or regions from PI 416937 related to the fitness of the progeny.

**Fig 2:**
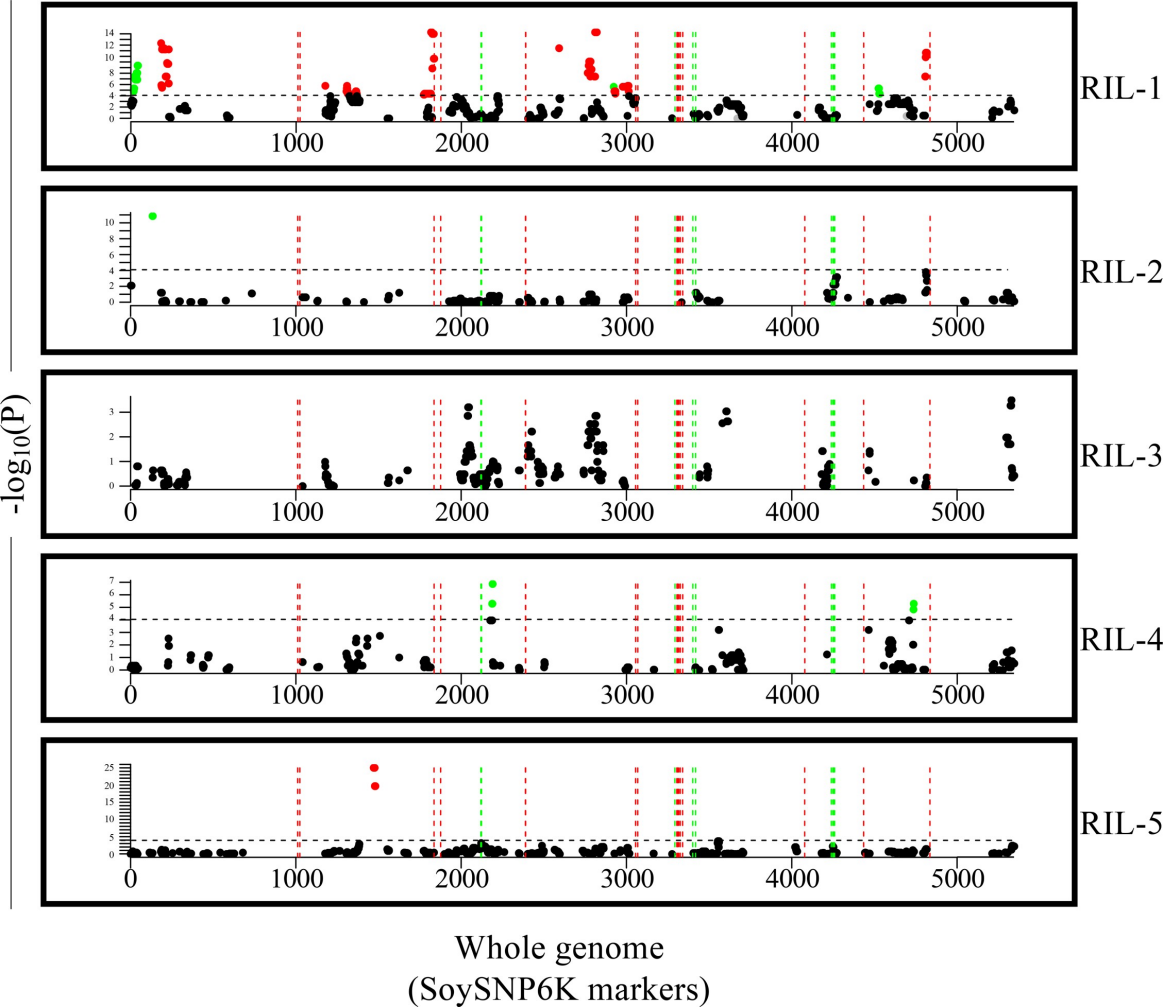
Genomic regions from PI 416937 under positive (green) and negative (red) selection within RIL populations at whole-genome level. The x-axis displays marker positions from SoySNP6K Infinium BeadChips. The statistical threshold was set for each population based on an LD adjusted multiple test correction. Gray indicates a locus had less than 10 trios testing a PI 416937 allele against an alternative allele. Black indicates a locus had 10 trios or more but fell below our significance threshold. Vertical dashed lines indicate regions identified under breeding selection from the original pedigree analysis performed on high yielding lines derived from PI 416937.

Eighteen regions under selection from RIL-1 were identified across 10 Chrs (Fig 2 and S4 Table). Three regions were under positive selection on Chrs 1 (1 regions), 11 (1 region), and 18 (1 region). Fifteen regions were found under negative selection on Chrs 1 (1 region), 2 (1 region), 5 (1 region), 6 (2 regions), 7 (3 regions), 10 (1 region), 11 (3 regions), 12 (2 regions), and 19 (1 region). RIL-2 had a single region under positive selection on Chr 1 (Fig 2 and S4 Table). RIL-3 had no regions under selection (Fig 2). RIL-4 had 2 regions under positive selection (Fig 2 and S4 Table). One region was on Chr 8 while the other was on Chr 18. RIL-5 had a single region under negative selection on Chr 6 (Fig 2 and S4 Table).

The region with the most significant evidence of positive selection (*P* = 1.39 × 10^−11^) was a 45,711 bp region in RIL-2 located on Chr 1. This level of significance far surpassed the most significant region under positive selection in the PI 416937 pedigree analysis (*P* = 2.49 × 10^−3^). The most significant region under negative selection in the RIL pedigree analysis was a 2,227,287 bp region located on Chr 6 and discovered within the RIL-5 population. This region was a peak within a large 4,143,902 bp region. An even greater significance discrepancy was seen for regions under negative selection when comparing to the PI 416937 pedigree analysis (*P* = 9.19 × 10^−26^ vs. 1.95 × 10^−3^). The full results from the RIL pedigree analysis are presented in S3 File.

## Discussion

In this study, we utilized extensive pedigree and genotypic information to identify genomic regions from PI 416937 with evidence of positive and negative selection. Five RIL populations were then used in an attempt to validate these regions of interest in replicated yield trials, but minimal significant yield associations were discovered. Genomic regions from PI 416937 were also under strong selection within the RIL populations but appeared to be population specific and did not overlap with results from the PI 416937 pedigree analysis.

Considering the prevalence of PI 416937 in the pedigrees of high yielding lines within the USDA Uniform Tests, it was possible that there was favorable selection for the PI 416937 genome as a whole, but we found no consistent trend for greater inheritance of PI 416937 genome than expected by pedigree amongst PI 416937-derived lines. Considering PI 416937 is a plant introduction that was originally received by the U.S. National Plant Germplasm System in 1974 [63], it is unlikely to compete with more modern elite germplasm in terms of seed yield per se [64] and the likelihood is high of haplotypes across the genome that are neutral or detrimental to yield potential. Researchers have also found unfavorable agronomic traits associated with PI 416937 such as susceptibility to Soybean Cyst Nematode (*Heterodera glycines*) race 3,5, and 14 [65], making it unlikely for breeders to select for PI 416937 on a whole genome level. The pedigree analysis provided additional resolution to identify more specific haplotypes from PI 416937 that had evidence of selection during breeding efforts.

Once genomic regions from PI 416937 under favorable selection were identified, a comparison was made to determine if these genomic regions overlapped with QTL previously found for water use efficiency [25], aluminum tolerance [21], root morphology [20], drought tolerance [66], and canopy wilting [15]. There was no overlap discovered. Advanced yield trials conducted by public breeders tend to be managed more intensively to reduce stressors such as drought. Many of the QTL mapped from PI 416937 examined tolerance to drought related conditions so it may not be a surprise that QTL mapped from crosses involving PI 416937 do not heavily overlap with regions found to be associated with seed yield. Also, this study is looking to identify regions that are under selection across diverse environments in predominantly North Carolina and Georgia as well as across diverse genetic backgrounds from these two breeding programs.

Only one of the PI 416937 haplotypes segregating within tested RIL populations showed a detectable influence on yield (*P* < 0.05). Other than genomic regions having been identified due to type I error, one possible explanation is that haplotype effects are confounded by genetic background effects when tested within RIL populations, providing further evidence of the difficulty of discovering significant seed yield QTL that transcend genetic background and environmental influence. Concibido et al. [7] reported some success of introgressing a yield QTL from *Glycine soja* (PI 407305) into soybean line, HS-I. When the QTL was introgressed into other elite backgrounds, inconsistencies arose in the reported yield effects. The QTL appeared to show limited adaptability across all genetic backgrounds. It is difficult to capture the true impact of an individual region on seed yield for several reasons. Seed yield is a highly quantitative trait which is heavily impacted by the environment and prone to phenotypic errors (e.g., combine error, un-accounted for field effects). Though this study was seeking to discover regions that could overcome genetic and environmental heterogeneity, it is also possible that PI 416937 haplotypes were competing against comparable or superior haplotypes. Another factor potentially contributing to a lack of statistical effect on yield associated with these PI 416937 haplotypes was G × E. Forty-four of the 52 PI 416937-derived lines were developed within the USDA Raleigh soybean breeding program and thus went through early stage development and yield testing in predominantly North Carolina environments, while the RIL populations used for yield testing were grown predominantly in Georgia environments.

The Yld1 region was a finely defined region (3.7 kb) in the PI 416937 pedigree analysis. Considering that the Yld1 region had been so narrowly defined, was supported by yield evaluations in a previous study, and showed strong evidence of breeder selection evidenced by being the eighth highest genomic region under positive selection across the entire genome, we felt this region was worthy of further exploration. Within this interval, there was a single gene model present for *Glyma*.*08g299800* which is a paralog to ATG24090.1, a chitinase A found in Arabidopsis. *Glyma*.*08g299800* is located from 41,795,912 to 41,796,546 bp (http://soybase.org) [47], which partially overlaps with the Yld1 region located from 41,792,467 to 41,796,167 bp. Chitinases are commonly associated with plant defense against fungal pathogens or insects as chitin is a common component of fungal cell walls and insect exoskeletons [67]. There are several QTL for various different traits that have been mapped to this region, one of which is ‘sclero 9–2’, a QTL related to fungal resistance [68]. ‘Sclero 9–2’ was a QTL associated with resistance to *Sclerotinia sclerotiorum* and was mapped from a cross of PI 391589B × IA2053. The favorable allele for this QTL was inherited from IA2053 which was the moderately susceptible parent in the cross. As a result of the climate conditions of the southeastern USA, it is reasonable to assume that fungal pressure is a constant concern. In Georgia from 2005–2013, an average of $2.7 million was spent annually on plant disease control from biotic stressors, primarily Asian soybean rust (*Phakopsora pachrhizi*) (UGA CAES, 2017) [69]. If the PI 416937 haplotype at Yld1 is providing moderate resistance to fungal pathogens, it is understandable why it would show an association with seed yield in southern U.S. breeding lines, although this pressure varies dramatically by year, which would lead to substantial G × E. Interestingly, Eickholt [58] reported the presence of a QTL × environment interaction associated with Yld1 across three locations, also suggesting the PI 416937 haplotype may impart some level of fungal disease resistance. The single location in which a significant increase in yield was not detected was also the only environment in which a systemic foliar fungicide was applied, possibly mitigating the beneficial effects of Yld1. The association of Yld1 with fungal pathogen resistance needs to be verified in further experiments.

**Fig 3:**
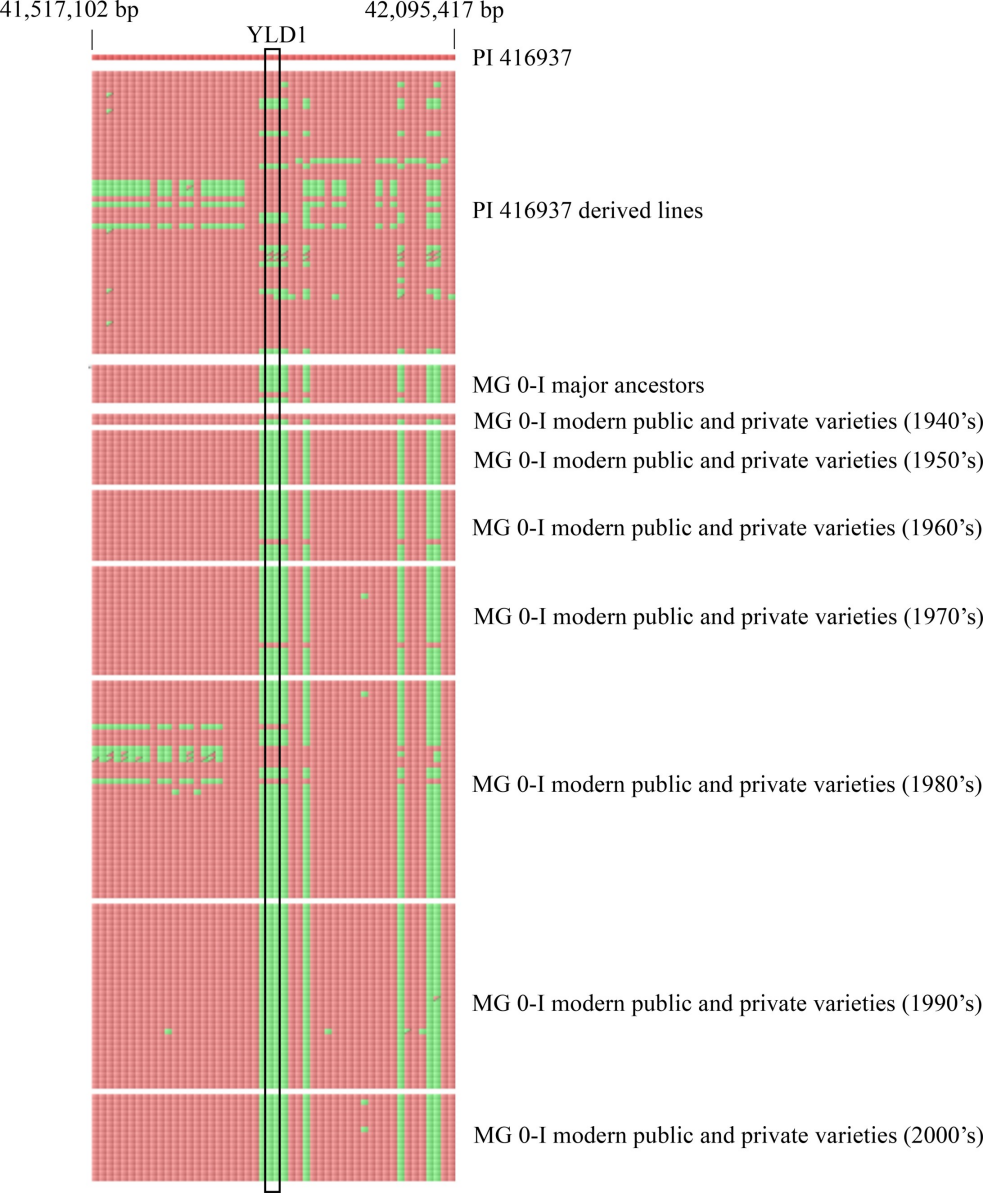
Haplotype diversity of the Yld1 locus on chromosome 8 among ancestral lines and modern varieties from MG 0-I. The top line indicates the PI 416937 haplotype. The second section displays haplotypes for all 52 high yielding PI 416937-derived lines used in our pedigree analysis. The third section displays haplotypes for the major ancestors of MG 0-I according to Vaughn and Li [53]. The bottom section displays haplotypes for modern public and private varieties bred for MG 0-I by decade of release. Red blocks are alleles identical to PI 416937 while green blocks are the alternative alleles for each locus. The Yld1 region is highlighted with a black outline. Visualization was performed using Flapjack–graphical genotype visualization [70].

Though Yld1 did not show a statistical impact on yield within our yield evaluations of several bi-parental RIL populations, it is often difficult to detect yield effects of single locus in this manner considering aforementioned confounding factors and other pieces of evidence. The results would point to Yld1 as a possible candidate for introgression of beneficial diversity, especially into regions of low diversity within modern soybean cultivars. Vaughn and Li [53] observed strong population structure in modern soybean cultivars that was heavily influenced by MG. They showed that North American soybean cultivars tended to group into three major groups based on genetic similarity. These groups span maturity ranges MG0-I, MG III-IV, and MG V+. Within these different groups, Vaughn and Li [53] then identified the founding ancestors and assessed regions of reduced diversity caused by similarity in the founding ancestors or possibly early selection. The PI 416937 pedigree analysis results were compared to those results to observe if any regions corresponded to regions with limited diversity in modern North American soybean varieties. The only region showing overlap was the Yld1 locus which overlapped with a low diversity region that Vaughn and Li [53] identified on Chr 8. The region of low diversity was discovered in the MG 0-I population and located between 41,517,102 and 42,095,417 bp (Gmax2.0) (Fig 3). A single haplotype was found in a majority of the founding ancestors for this population and therefore had limited diversity prior to decades of breeding selection. The region quickly lost this diversity during early breeding stages. From these results, this region can be interpreted as having fairly limited diversity among the North American ancestor lines and quickly lost that diversity within the MG 0-I population. Gizlice et al. [2] also determined that 80% of the northern genetic base can be accounted for by 10 ancestral lines by pedigree.

We used a panel of 135 modern soybean breeding lines (MG 0-I), which had been released each decade from the 1940s to the 2000s, to visualize the haplotype diversity for this region in the past through more modern breeding material. The major haplotype which is prevalent within the MG 0-I ancestor lines is the predominant haplotype shared within the MG 0-I breeding material each decade. PI 548572 ‘Harly’ (1940s), PI 548550 ‘Disoy’ (1960s), PI 548536 ‘Coles’ (1970s), and PI 548640 ‘OAC Scorpio’ (1980s) were the only four lines of the 135 which contained the PI 416937 haplotype for this region. Even though the markers were a genotypic match to PI 416937, it could not be determined if this region was completely identical at all SNP loci unless sequencing was performed on all lines. Regardless, the PI 416937 region was sparsely present. This finding suggests there is an opportunity for targeted introgression of beneficial diversity into a region of low diversity for MG 0-I breeding materials. This region did not contain any noted maturity QTL from the literature, so it is unlikely that this region has become fixed as a result of fixing maturity related genes. There may be other environmental factors for MG 0-I breeders that have led to the fixation of this region for this particular haplotype. It may also be the case that the MG 0-I region that appears endemic to this locus has lacked competition from other potentially superior haplotypes.

Vaughn and Li [53] did not report the aforementioned genomic region as low diversity in MG III-IV or V+. In this study, when investigating the haplotype diversity within this region for MG III-IV, the ancestral lines as well as modern cultivars appeared to be dominated by the same haplotype which was prevalent in MG 0-I though not to quite as severe an extent (S5 Fig). This dominant haplotype was also common among MG V+ ancestral lines and modern cultivars, but there did appear to be more haplotype diversity in these southern U.S. cultivars (S6 Fig). The PI 416937 haplotype was not present among the ancestral lines of MG III-IV nor MG V+ but was found in nine of 198 MG III-IV cultivars (S5 Fig) and five of 114 MG V+ cultivars (S6 Fig). Though more haplotype diversity was present in MG III-IV and MG V+ relative to MG0-I, the rarity of the PI 416937 haplotype and seeming dominance of other haplotypes indicates potential for these maturity groups to benefit from introgression of the PI 416937 haplotype as well.

Genomic regions such as Yld1 showed evidence of selection across high yielding PI 416937-derived lines, but it is possible that regions may increase in frequency due to segregation distortion, so a similar pedigree analysis was performed within the RIL populations. Each RIL population shared between 88 to 97 percent of tested PI 416937 alleles across the other RIL populations so there was replication in which PI 416937 alleles were being tested across populations. There was no overlap between regions under selection across RIL populations (Fig 2 and S4 Table). Thus, these regions appeared to be population and possibly environment specific. McMullen et al. [71] examined segregation distortion extensively across and within the 25 families of the maize nested associated mapping population. Though significant distortion was detected, genomic regions experiencing distortion were often population specific and the alleles under favorable selection could also vary depending upon the population. Environmental stressors may also arise during early stage testing which can influence these signals of selection. Potential environmental stressors can range from biotic stressors such as pathogens or pests to abiotic stressors such as drought or flooding. Breeders also tend to select on favorable agronomic traits such as standability. There was no overlap of regions under selection in the RIL pedigree analysis and QTL mapping results from studies involving PI 416937 to indicate a likely source of environmental influence for signals of selection.

There was also no overlap between the RIL pedigree analysis and the PI 416937 pedigree analysis (Fig 2 and S4 Table). We examined whether a discrepancy between marker density in the SoySNP50K and SoySNP6K Infinium BeadChips may be explaining the lack of overlap in results. We concluded this is unlikely because 18 of 26 regions under selection in the PI 416937 pedigree analysis contained markers from the 6K BeadChip within the region under selection. The remaining 8 regions had a flanking 6K BeadChip marker no more than 42 Kb and on average, 19 Kb, away. A limiting factor which largely explains the lack of overlap with the PI 416937 pedigree analysis is that only seven (5, 6, 19, 21, 22, 23, 24) of the 26 regions identified in the PI 416937 pedigree analysis were segregating for a PI 416937 haplotype within the RIL populations. Though we could not capture all of the PI 416937 genome among the parents of these RIL populations, we were able to examine potential overlap for a sampling of these regions.

The significance of selection for many of these regions far surpassed the level of selection within the PI 416937 pedigree analysis. Regions identified in the RIL pedigree analyses may not be responsible for significant changes in seed yield as much as they are responsible for fitness or visual cues of vigor that breeders observe when selecting lines to be placed in advanced replicated yield trials. Though not all PI 416937-derived regions with evidence of selection were segregating within the RIL populations, this result increases our confidence that regions under selection within the PI 416937 pedigree analysis were not a result of segregation distortion, but rather selection during extensive yield evaluations. Regions from PI 416937 which decreased fitness would be expected to last only a single generation and would have been removed in the progenitor trio. These regions would have had one test in the PI 416937 pedigree analysis and thus would never have been detected as significantly under negative selection. As for regions from PI 416937 under positive selection in the RIL pedigree analysis, these would arise in a single cross but when PI 416937 was crossed with other materials, these regions would no longer be under such strong selection unless the alternative alleles in every parent of every cross decreased fitness, which is highly unlikely. The pedigree analysis implemented for PI 416937 identifies signals of selection resulting from the culmination of breeding efforts from early stage screening to replicated yield testing across genetic backgrounds and environments which differentiates from the RIL pedigree analysis which identifies population/environment specific signals associated with segregation distortion or early stage vigor.

## Conclusion

Concerns have been expressed that the current selection pressures and continuous elite-by-elite crosses will lead to a ‘breeding plateau’ for genetic gains as well as greater susceptibility to disease and insect pressure [72]. As the breeding process has narrowed the genetic diversity of elite material, there is increasing pressure to discover exotic or wild alleles that are beneficial and can be incorporated selectively into elite breeding material while avoiding other alleles which are less agronomically favorable. Breeders are hesitant to break up favorable linkage blocks [40] and sacrifice yield and vigor for increased diversity from more exotic germplasm [73]. The strategies outlined here combine detailed pedigree knowledge and high-density genotyping to enable discovery of beneficial alleles across genetic backgrounds and environments. This study focused on identifying beneficial haplotypes from a PI amongst a genomic background which may be less agronomically favorable and these alleles are prime candidates to challenge regions of low diversity in modern soybean cultivars. Especially for genomic regions lacking diversity in the founding ancestors of North American soybean cultivars, new alleles have potential to increase yield gains. Even genomic regions which have had a history of diversity should be challenged with new alleles in an effort to achieve continuous gains. Though the genomic regions discovered could not be validated using the methods at our disposal, the methodology displayed in this study opens the door for new ways to approach finding and exploiting beneficial diversity to overcome future plateauing in genetic gain for soybean breeders associated with continuous elite by elite crossing for breeding population development. Cregan [74] predicted a future in which tens of thousands of soybean cultivars would be genotypically characterized and a future question facing soybean genomicists will be how to mine this data for the improvement of soybean. We believe we have proposed an effective strategy for utilizing genomic data to improve soybean via identification of potentially beneficial diversity.

## Supporting information

S1 File A collection of files depicting results from the PI 416937 pedigree analysis for each individual chromosome. The top section indicates genomic regions across chromosome 8 under significant positive (green) versus negative selection (red). The statistical threshold was set at a–log_10_ *P*-value of 1.56 (Yld1). Gray indicates a locus had less than 10 tests. Black indicates a locus had 10 tests or more but fell below our significance threshold. The bottom portion of the figure displays the chromosomal inheritance for each trio broken up by unique crosses. The top two lines for each cross are the parents while the bottom lines are the high yielding PI 416937-derived progeny from each cross. For the parents, red indicates a chromosomal region inherited from PI 416937. Black indicates a chromosomal region inherited from a major southern ancestor [53]. For the progeny, orange indicates chromosomal inheritance from the top parent and blue indicates inheritance from the bottom parent. Gray for both parents and progeny indicates chromosomal inheritance was ambiguous.
(DOCX)

S2 File Full results for the PI 416937 pedigree analysis. (XLSX)

S3 File Full results for the RIL pedigree analysis. (XLSX)

S1 Fig Pedigree tree of PI 416937-derived lines along with the ancestors traced back to earliest decipherable ancestors. Each genotype is represented by a square with lines connecting parents to progeny. Purple indicates that genotypes were derived from PI 416937 and included in the pedigree analysis. Blue corresponds to that genotypes derived from PI 416937 that were not included in the pedigree analysis. Turquoise indicates PI 416937. Green corresponds to genotypes that were not derived from PI 416937, but included in the pedigree analysis. Yellow indicates that genotypes were not derived from PI 416937 and not included in the pedigree analysis. For breeding lines with greater than 6 progeny, squares vary in size based upon how many direct progeny are derived from a particular line. Genotypes were coded in S1 Fig as numbers which were defined in S3 Table.
(TIF)

S2 Fig Cladogram showing population structure of high yielding PI 416937-derived lines relative to soybean ancestors and modern varieties colored by MG. (TIF)

S3 Fig Cladogram showing population structure of high yielding PI 416937-derived lines relative to ancestors and modern varieties colored by descriptors. (TIF)

S4 Fig **Genomic regions from PI 416937 under positive (green) and negative (red) selection across the whole genome.** The x-axis displays marker positions from SoySNP50K Infinium BeadChips. The statistical threshold was set at a–log_10_ *P*-value of 1.56 (Yld1). Gray indicates a locus had less than 10 trios testing a PI 416937 allele against an alternative allele. Black indicates a locus had 10 trios or more but fell below our significance threshold.
(TIF)

S5 Fig Haplotype diversity of the Yld1 locus on chromosome 8 among ancestral lines and modern varieties from MG III-IV. The top line indicates the PI 416937 haplotype. The second, third, and fourth sections display haplotypes for all 52 high yielding PI 416937-derived lines used in our pedigree analysis, the major ancestors of MG III-IV according to Vaughn and Li (2016), and modern public and private cultivars bred for MG III-IV by decade of release, respectively. Red blocks are alleles identical to PI 416937 while green blocks are the alternative alleles for each locus. The Yld1 locus is highlighted with a black outline. Visualization was performed using Flapjack–graphical genotype visualization [70].
(TIF)

S6 Fig Haplotype diversity of the Yld1 locus on chromosome 8 among ancestral lines and modern varieties from MG V+. The top line indicates the PI 416937 haplotype. The second, third, and fourth sections display haplotypes for all 52 high yielding PI 416937-derived lines used in our pedigree analysis, the major ancestors of MG V+ according to Vaughn and Li (2016), and modern public and private cultivars bred for MG V+ by decade of release, respectively. Red blocks are alleles identical to PI 416937 while green blocks are the alternative alleles for each locus. The Yld1 locus is highlighted with a black outline. Visualization was performed using Flapjack–graphical genotype visualization [70].
(TIF)

S1 Table Description of RIL populations. (DOCX)

S2 Table List of genotypes used in cladograms. (DOCX)

S3 Table List of genotypes included in S1 Fig. (DOCX)

S4 Table Results of whole-genome based RIL pedigree analysis. (DOCX)

## Abbreviations

MG: maturity group
RIL: recombinant inbred line
SNP: single nucleotide polymorphism
QTL: quantitative trait locus
LD: linkage disequilibrium

## References

[1] Kovach MJ, McCouch SR. Leveraging natural diversity: back through the bottleneck. Curr Opin Plant Biol. 2008; 11: 193–200. 10.1016/j.pbi.2007.12.006 18313975

[2] Gizlice Z, Carter TE, Burton JW. Genetic base for North American public soybean cultivars released between 1947 and 1988. Crop Sci. 1994; 34: 1143–1151.

[3] Panthee DR. Varietal improvement in soybean In: Singh G., editor. The soybean: botany, production and uses. CAB International, Oxfordshire, UK 2010 pp. 92–112.

[4] Hittalmani S, Parco A, Mew TV, Zeigler RS, Huang N. Fine mapping and DNA marker-assisted pyramiding of the three major genes for blast resistance in rice. Theor Appl Genet. 2000; 100: 1121–1128.

[5] Singh S, Sidhu JS, Huang N, Vikal Y, Li Z, Brar DS, et al Pyramiding three bacterial blight resistance genes (*xa5*, *xa13 and Xa21*) using marker-assisted selection into indica rice cultivar PR106. Theor Appl Genet. 2001; 102: 1011–1015.

[6] Castro AJ, Capettini F, Corey AE, Filichkina T, Hayes PM, Kleinhofs A, et al Mapping and pyramiding of qualitative and quantitative resistance to stripe rust in barley. Theor Appl Genet. 2003; 107: 922–930. 10.1007/s00122-003-1329-6 12845434

[7] Concibido VC, La Vallee B, Mclaird P, Pineda N, Meyer J, Hummel L, et al Introgression of a quantitative trait locus for yield from *Glycine soja* into commercial soybean cultivars. Theor Appl Genet. 2003; 106: 575–582. 10.1007/s00122-002-1071-5 12595984

[8] Ude GN, Kenworthy WJ, Costa JM, Cregan PB, Alvernaz J. Genetic diversity of soybean cultivars from China, Japan, North America, and North American ancestral lines determined by amplified fragment length polymorphism. Crop Sci. 2003; 43: 1858–1867.

[9] Boerma HR, Hussey RS, Phillips DV, Wood ED. Soybean variety G00-3209. 2012 Google Patents.

[10] Boerma HR, Hussey RS, Phillips DV, Wood ED, Rowan GB, Finnerty SL. Registration of ‘Benning’ soybean. Crop Sci. 1997; 37: 1982.

[11] Paris RL, Bell PP. Uniform soybean tests, southern states 2003. USDA-ARS, Stoneville, MS 2003.

[12] Paris RL, Bell PP. Uniform soybean tests, southern states 2004. USDA-ARS, Stoneville, MS 2004.

[13] Paris RL, Shelton GW, Bell PP. Uniform soybean tests, southern states 2005. USDA-ARS, Stoneville, MS 2005.

[14] King CA, Purcell LC, Brye KR. Differential wilting among soybean genotypes in response to water deficit. Crop Sci. 2009; 49: 290–298.

[15] Abdel-Haleem H, Carter TE, Purcell LC, King CA, Ries LL, Chen PY, et al Mapping of quantitative trait loci for canopy-wilting trait in soybean (*Glycine max* L. Merr). Theor Appl Genet. 2012; 125: 837–846. 10.1007/s00122-012-1876-9 22566068

[16] Hwang S, King CA, Ray JD, Cregan PB, Chen P, Carter TE, et al Confirmation of delayed canopy wilting QTLs from multiple soybean mapping populations. Theor Appl Genet. 2015; 128: 2047–2065. 10.1007/s00122-015-2566-1 26163767

[17] Shin JH, Vaughn JN, Abdel-Haleem H, Chavarro C, Abernathy B, Kim KD, et al Transcriptomic changes due to water deficit define a general soybean response and accession-specific pathways for drought avoidance. BMC Plant Biol. 2015; 15: 26 10.1186/s12870-015-0422-8 25644024 PMC4322458

[18] Pantalone VR, Rebetzke GJ, Burton JW, Carter TE. Phenotypic evaluation of root traits in soybean and applicability to plant breeding. Crop Sci. 1996; 36: 456–459.

[19] Pantalone VR., Rebetzke GJ, Burton JW, Carter TE, Israel DW. Soybean PI 416937 root system contributes to biomass accumulation in reciprocal grafts. Agron J. 1999; 91: 840–844.

[20] Abdel-Haleem H, Lee GJ, Boerma HR. Identification of QTL for increased fibrous roots in soybean. Theor Appl Genet. 2011; 122: 935–946. 10.1007/s00122-010-1500-9 21165732

[21] Bianchi-Hall CM, Carter TE, Bailey MA, Mian MAR, Rufty TW, Ashley DA, et al Aluminum tolerance associated with quantitative trait loci derived from soybean PI 416937 in hydroponics. Crop Sci. 2000; 40: 538–545.

[22] Villagarcia MR, Carter TE, Rufty TW, Niewoehner AS, Jennette MW, Arrellano C. Genotypic rankings for aluminum tolerance of soybean roots grown in hydroponics and sand culture. Crop Sci. 2001; 41: 1499–1507.

[23] Sloane RJ, Patterson RP, Carter TE. Field drought tolerance of a soybean plant introduction. Crop Sci. 1990; 30: 118–123.

[24] Hudak CM RP Patterson. Vegetative growth analysis of a drought-resistant soybean plant introduction. Crop Sci. 1995; 35: 464–471.

[25] Mian MAR, Bailey MA, Ashley DA, Wells R, Carter TE, Parrott WA, et al Molecular makers associated with water use efficiency and leaf ash in soybean. Crop Sci. 1996; 36: 1252–1257.

[26] Hufstetler EV, Boerma HR, Carter TE, Earl HJ. Genotypic variation for three physiological traits affecting drought tolerance in soybean. Crop Sci. 2007; 47: 25–35.

[27] Carter TE, Burton JW, Fountain MO, Rzewnicki PE, Villagarcia MR, Bowman DT. Registration of ‘N7002’ soybean. J Plant Reg. 2007; 1: 93–94.

[28] Carter TE, Burton JW, Fountain MO, Rzewnicki PE, Villagarcia MR, Bowman DT. Registration of ‘N8001’ soybean. J Plant Reg. 2008; 2: 22–23.

[29] Carter TE, Todd SM, Gillen AM. Registration of ‘USDA-N8002’ soybean cultivar with high yield and abiotic stress resistance traits. J Plant Reg. 2016; 10: 238–245.

[30] Gillen AM, Shelton GW. Bell PP. Uniform soybean tests, southern states 2007. USDA-ARS, Stoneville, MS 2007.

[31] Gillen AM, Shelton GW. Bell PP. Uniform soybean tests, southern states 2008. USDA-ARS, Stoneville, MS 2008.

[32] Gillen AM, Shelton GW. Bell PP. Uniform soybean tests, southern states 2009. USDA-ARS, Stoneville, MS 2009.

[33] Gillen AM, Shelton GW. Bell PP. Uniform soybean tests, southern states 2010. USDA-ARS, Stoneville, MS 2010.

[34] Gillen AM, Shelton GW. Bell PP. Uniform soybean tests, southern states 2011. USDA-ARS, Stoneville, MS 2011.

[35] Paris RL, Shelton GW, Bell PP. Uniform soybean tests, southern states 2000. USDA-ARS, Stoneville, MS 2000.

[36] Paris RL, Bell PP. Uniform soybean tests, southern states 2001. USDA-ARS, Stoneville, MS 2001.

[37] Paris RL, Bell PP. Uniform soybean tests, southern states 2002. USDA-ARS, Stoneville, MS 2002

[38] Lorenzen LL., Boutin S, Young N, Specht JE, Shoemaker RC. Soybean pedigree analysis using map-based molecular markers: I. Tracking RFLP markers in cultivars. 1995; 35: 1326–1336.

[39] Sebastian SA, Tingey SV, Hanafey MK. Method to identify genetic markers that are linked to agronomically important genes. Google Patents. 1995.

[40] Grainger CM, Rajcan I. Characterization of the genetic changes in a multi-generational pedigree of an elite Canadian soybean cultivar. Theor Appl Genet. 2014; 127: 211–229. 10.1007/s00122-013-2211-9 24141573

[41] Clevenger J, Chu Y, Chavarro C, Agarwai G, Bertoili DJ, Leal-Bertioli SCM, et al Genome-wide SNP genotyping resolves signatures of selection and tetrasomic recombination in peanut. Mol Plant. 2017; 10: 309–322. 10.1016/j.molp.2016.11.015 27993622 PMC5315502

[42] Bink MC, Te Pas MF, Harders FL, Janss LL. A transmission/disequilibrium test approach to screen for quantitative trait loci in tow selected lines of large white pigs. Genet Res. 2000; 75: 115–121. 10.1017/s0016672399004061 10740927

[43] Jannink J, Bink MC, Jansen RC. Using complex plant pedigrees to map valuable genes. Trends Plant Sci. 2001; 6: 337–342. 10.1016/s1360-1385(01)02017-9 11495765

[44] Shoemaker RC, Guffy RD, Lorenzen LL, Specht JE. Molecular genetic mapping of soybean: Map utilization. Crop Sci. 1992; 32: 1091–1098.

[45] Brim CA. A modified pedigree method of selection in soybeans. Crop Sci. 1966; 6: 220.

[46] Devi JM, Sinclair TR, Chen P, Carter TE. 2014. Evaluation of elite southern maturity soybean breeding lines for drought-tolerant traits. Agron J. 2014; 106: 1947–1954.

[47] SoyBase. SoyBase: Soybean breeder’s toolbox. https://soybase.org/snps/download.php (Accessed April 2015).

[48] Shaw PD, Graham M, Kennedy J, Milne I, Marshall DF. Helium: Visualization of large scale plant pedigrees. BMC Bioinformatics. 2014; 15: 259 10.1186/1471-2105-15-259 25085009 PMC4133633

[49] Keim P, Shoemaker RC, Olson TC. A rapid protocol for isolating soybean DNA. Soybean Genet News. 1988; 15: 150–152.

[50] Song Q, Hyten DL, Jia G, Quigley CV, Fickus EW, Nelson RL, et al Development and evaluation of SoySNP50K, a high-density genotyping array for soybean. PLoS ONE. 2013; 8:e54985 10.1371/journal.pone.0054985 23372807 PMC3555945

[51] Song Q, Jia G, Quigley CV, Fickus EW, Hyten DL, Cregan PB. Soybean BARC-soySNP6K Beadchip-a tool for soybean genetics research. Poster presented at: plant and animal genome XXII conference, San Diego, CA, USA. January 10–15, 2014; Abstract no. P306.

[52] Schmutz J, Cannon SB, Schlueter J, Ma J, Mitros T, Nelson W, et al Genome sequence of the palaeopolyploid soybean. Nature. 2010; 463: 178–183. 10.1038/nature08670 20075913

[53] Vaughn JN, Li Z. Genomic signatures of North American soybean improvement inform diversity enrichment strategies and clarify the impact of hybridization. G3. 2016; 6: 2693–2705. 10.1534/g3.116.029215 27402364 PMC5015928

[54] R core team. R: A language and environment for statistical computing. R Foundation for Statistical Computing, Vienna, Austria URL http://www.R-project.org/. 2013.

[55] Bradbury PJ, Zhang Z, Kroon DE, Casstevens TM, Ramdoss Y, Buckler ES. TASSEL: software for association mapping of complex traits in diverse samples. Bioinformatics. 2007; 23: 2633–2635. 10.1093/bioinformatics/btm308 17586829

[56] Yu G, Smith D, Zhu H, Guan Y, Lam TT. ggtree: an R package for visualization and annotation of phylogenetic trees with their covariates and other associated data. Methods Ecol Evol. 2017; 8: 28–36.

[57] Clopper CJ, Pearson ES. The use of confidence or fiducial limits illustrated in the case of the binomial. Biometrika. 1934; 26: 404–413.

[58] Eickholt DPJ. Into the wild: Identification, introgression and validation of genetic diversity for use in applied soybean breeding (under the direction of Thomas E. Carter, Jr.). PhD Dissertation. 2016.

[59] De Bakker PIW, Yelensky R, Pe’er I, Gabriel SB, Daly MJ, Altshuler D. Efficiency and power in genetic association studies. Nat Genet. 2005; 37: 1217–1223. 10.1038/ng1669 16244653

[60] Barrett JC, Fry B, Maller J, Daly MJ. Haploview: analysis and visualization of LD and haplotype maps. Bioinformatics. 2005; 21: 263–265. 10.1093/bioinformatics/bth457 15297300

[61] SAS Institute. JMP version 13.0.0. SAS Inst., Cary, NC 2016.

[62] Wang, Lin. Planned versus unplanned contrasts: exactly why planned contrasts tend to have more power against type II error. Paper presented at: annual meeting of the mid-south education research association, New Orleans, LA, USA. November 9–12, 1993.

[63] NPGS. U.S. National Plant Germplasm System. https://npgsweb.ars-grin.gov/gringlobal/accession detail.aspx?id = 1314868 (Accessed April 2020).

[64] Hill JL, Peregrine EK, Sprau GL, Cremeens CR, Nelson RL, Kenty MM, et al Evaluation of the USDA soybean germplasm collection: maturity groups VI-VIII (FC 03.659-PI 567.235B). Techn. Bull. U.S.D.A. 2001; 1894.

[65] Young LD. Soybean germplasm evaluated for resistance to races 3, 5, 14 of soybean cyst nematode. Crop Sci. 1990; 30: 735–736.

[66] Carpentieri-Pipolo V, Pipolo AE, Abdel-Haleem H, Boerma HR, Sinclair TR. Identification of QTLs associated with limited leaf hydraulic conductance in soybean. Euphytica. 2012; 186: 679–686.

[67] Sharma N, Sharma KP, Gaur RK, Gupta VK. Role of chitinases in plant defense. Asian J of Biochem. 2011; 6: 29–37.

[68] Guo X, Wang D, Gordon SG, Helliwell E, Smith T, Berry SA, et al Genetic mapping of QTLs underlying partial resistance to *Sclerotinia scerotiorum* in soybean PI 391589A and PI 391589B. Crop Sci. 2008; 48: 1129–1139.

[69] UGA CAES. 2005–2013 Georgia Disease Loss Estimates. Georgia Plant Disease Loss Estimates. UGA CAES Plant Pathology Department. http://www.caes.uga.edu/departments/plant-pathology/extension/educational-materials/georgia-plant-disease-loss-estimates.html (accessed April 2017).

[70] Milne I, Shaw P, Stephen G, Bayer M, Cardle L, Thomas WTB, et al Flapjack–graphical genotype visualization. Bioinformatics. 2010; 26: 3133–3134. 10.1093/bioinformatics/btq580 20956241 PMC2995120

[71] McMullen MD, Kresovich S, Villeda HS, Bradbury P, Li H, Sun Q, et al Genetic properties of the maize nested association mapping population. Science. 2009; 325: 737–740. 10.1126/science.1174320 19661427

[72] Hyten DL, Song Q, Zhu Y, Choi IY, Nelson RL, Costa JM, et al Impacts of genetic bottlenecks on soybean genome diversity. Proc Natl Acad Sci. 2006; 103: 16666–16671. 10.1073/pnas.0604379103 17068128 PMC1624862

[73] Moose SP, Mumm RH. Molecular plant breeding as the foundation for 21st century crop improvement. Plant Phys. 2008; 147: 969–977. 10.1104/pp.108.118232 PMC2442525 18612074

[74] Cregan PB. Soybean molecular diversity In: Stacy Gary, editor. Genetics and Genomics of Soybean. Springer Science & Business Media, LLC New York; 2008 pp. 17–30.

