# Supplementary figures and images for: Characterizing the impact of an exotic soybean line on elite cultivar development
Source: PLoS One. 2020 Jul 10;15(7):e0235434. doi: 10.1371/journal.pone.0235434 (PMC7351202; doi:10.1371/journal.pone.0235434)

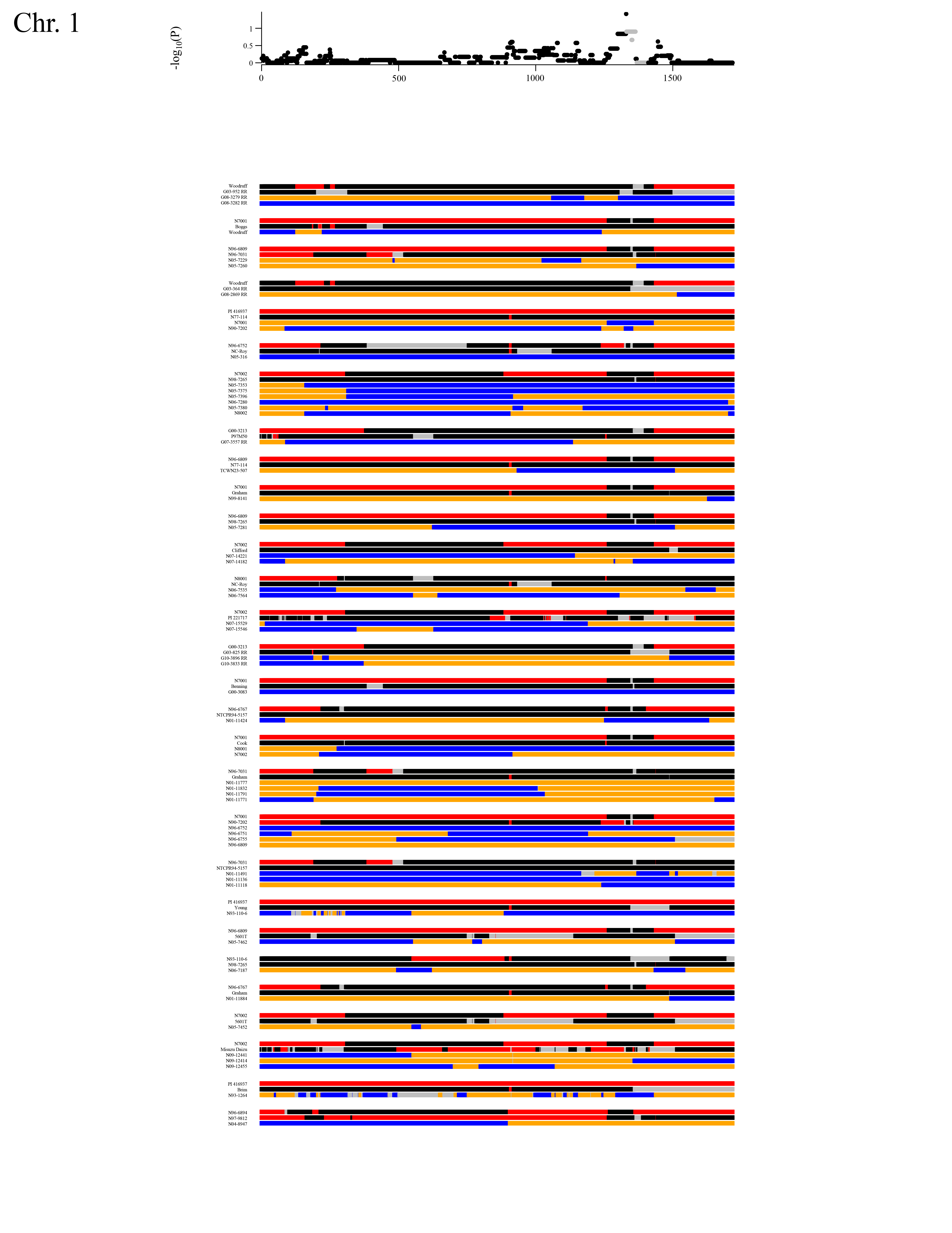

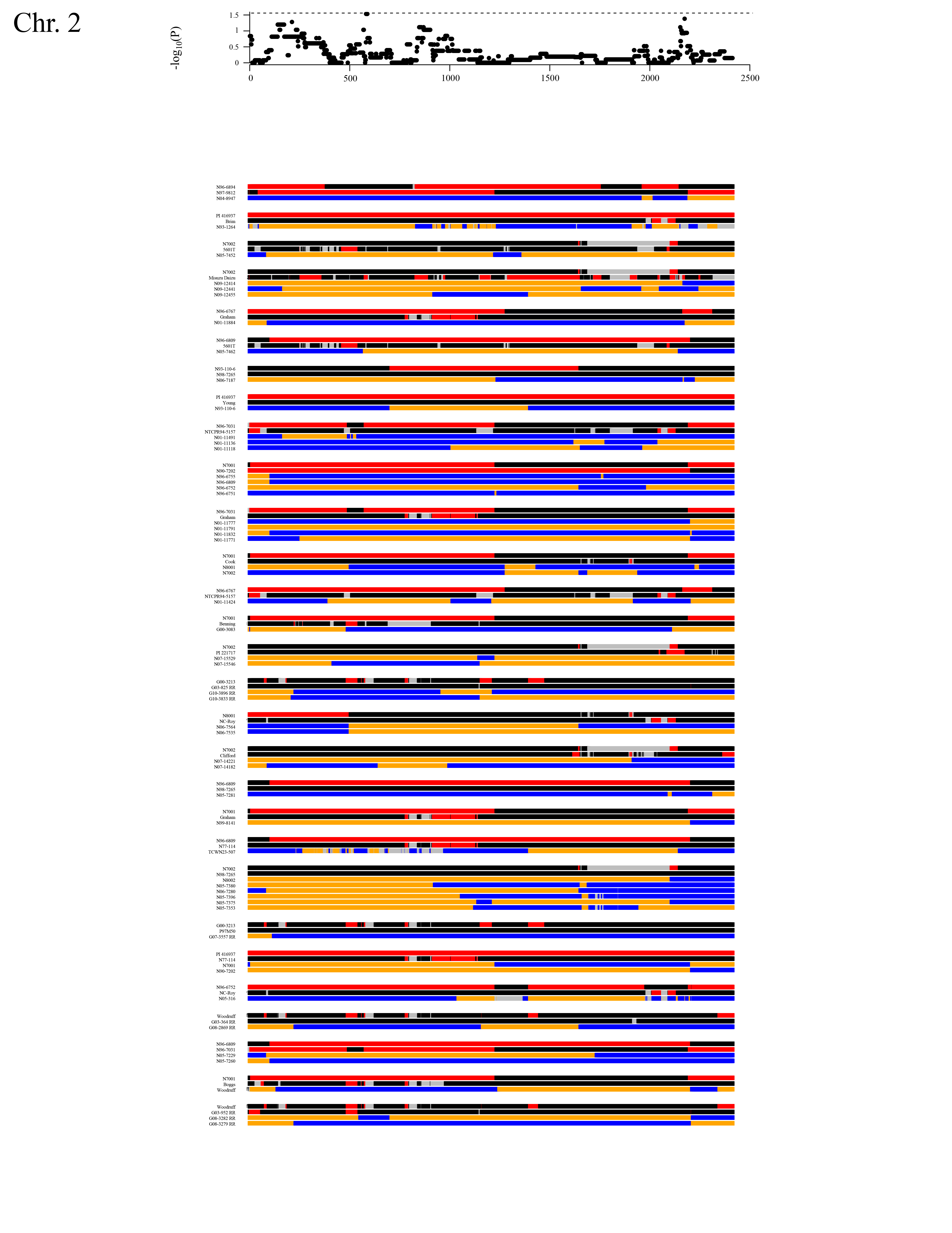

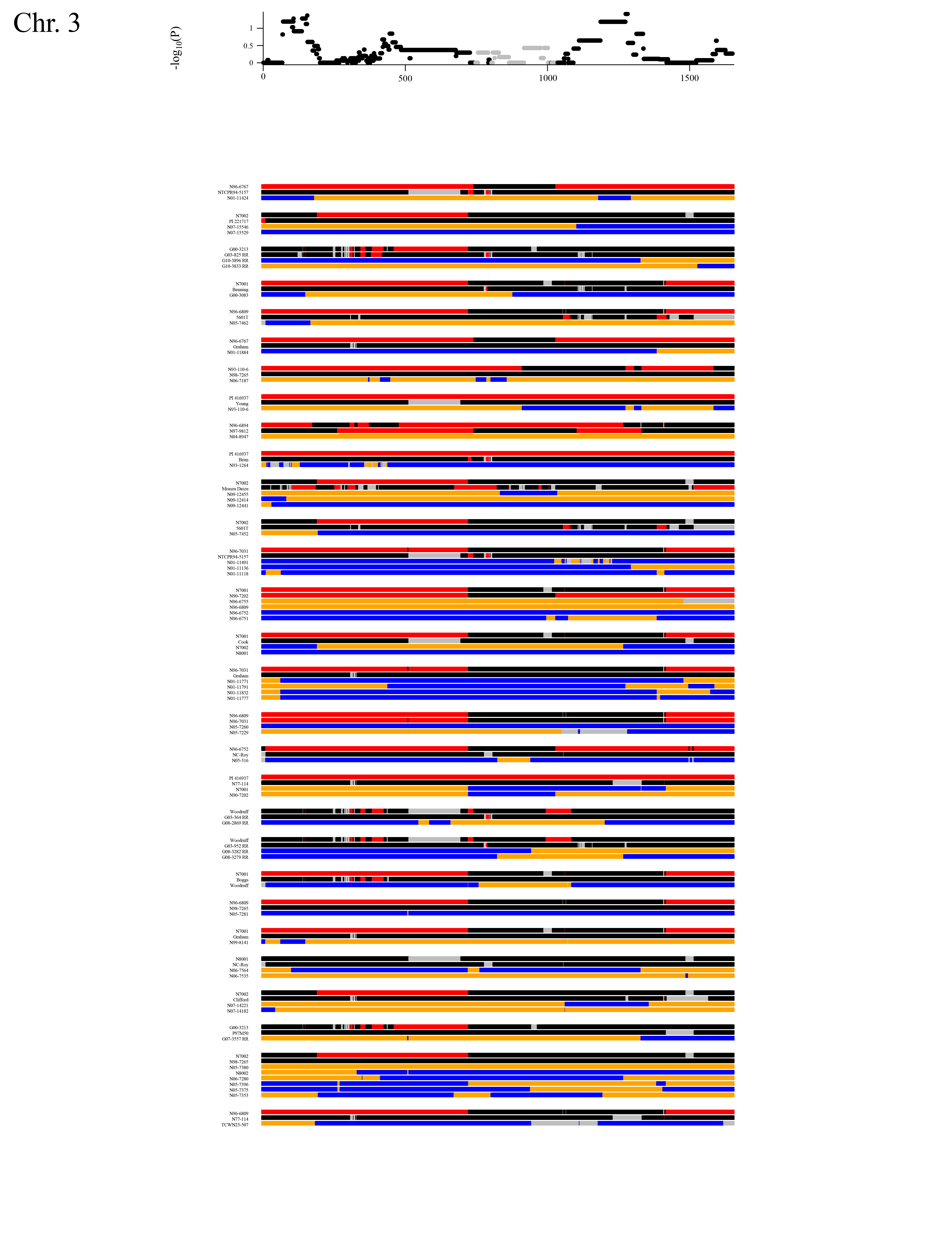

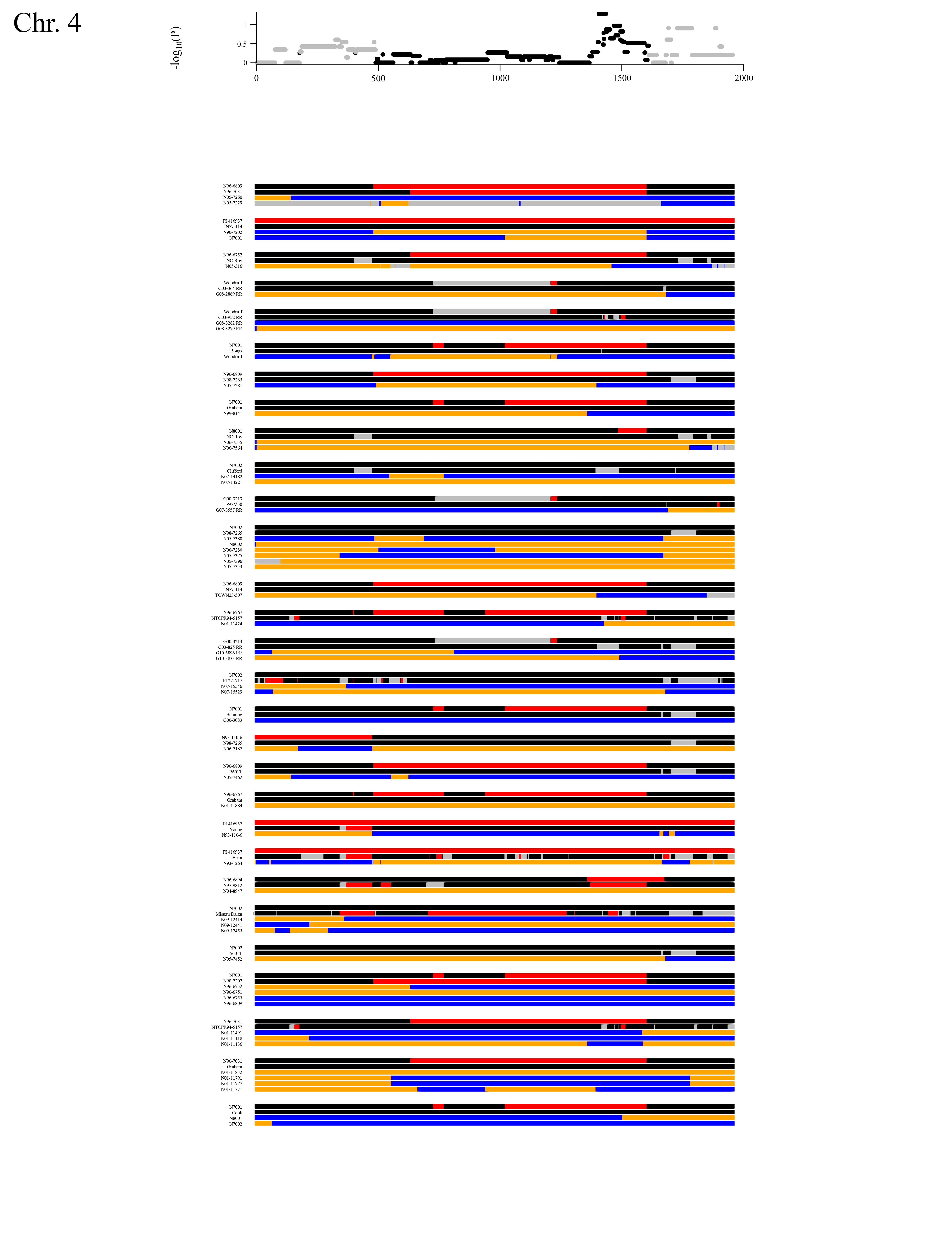

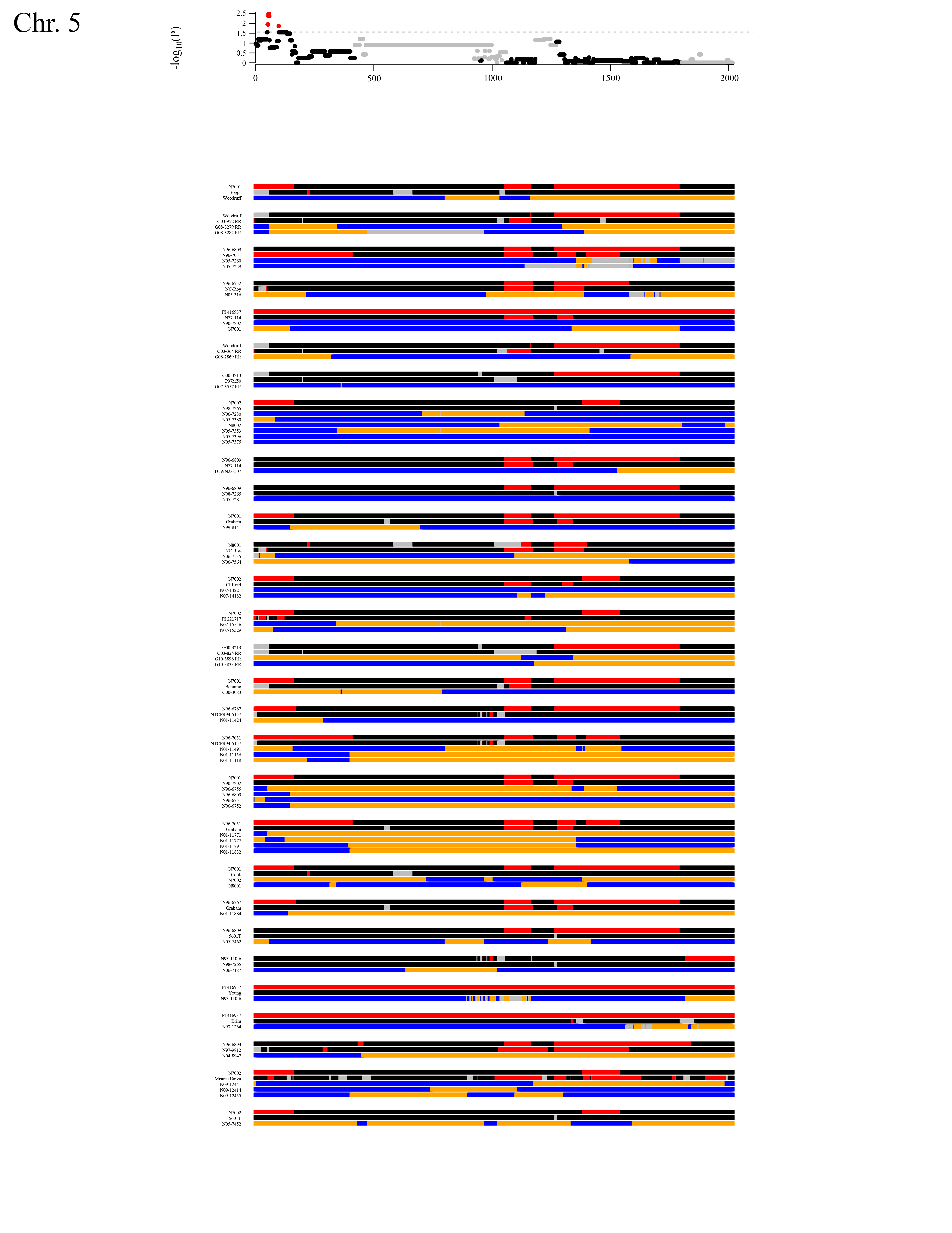

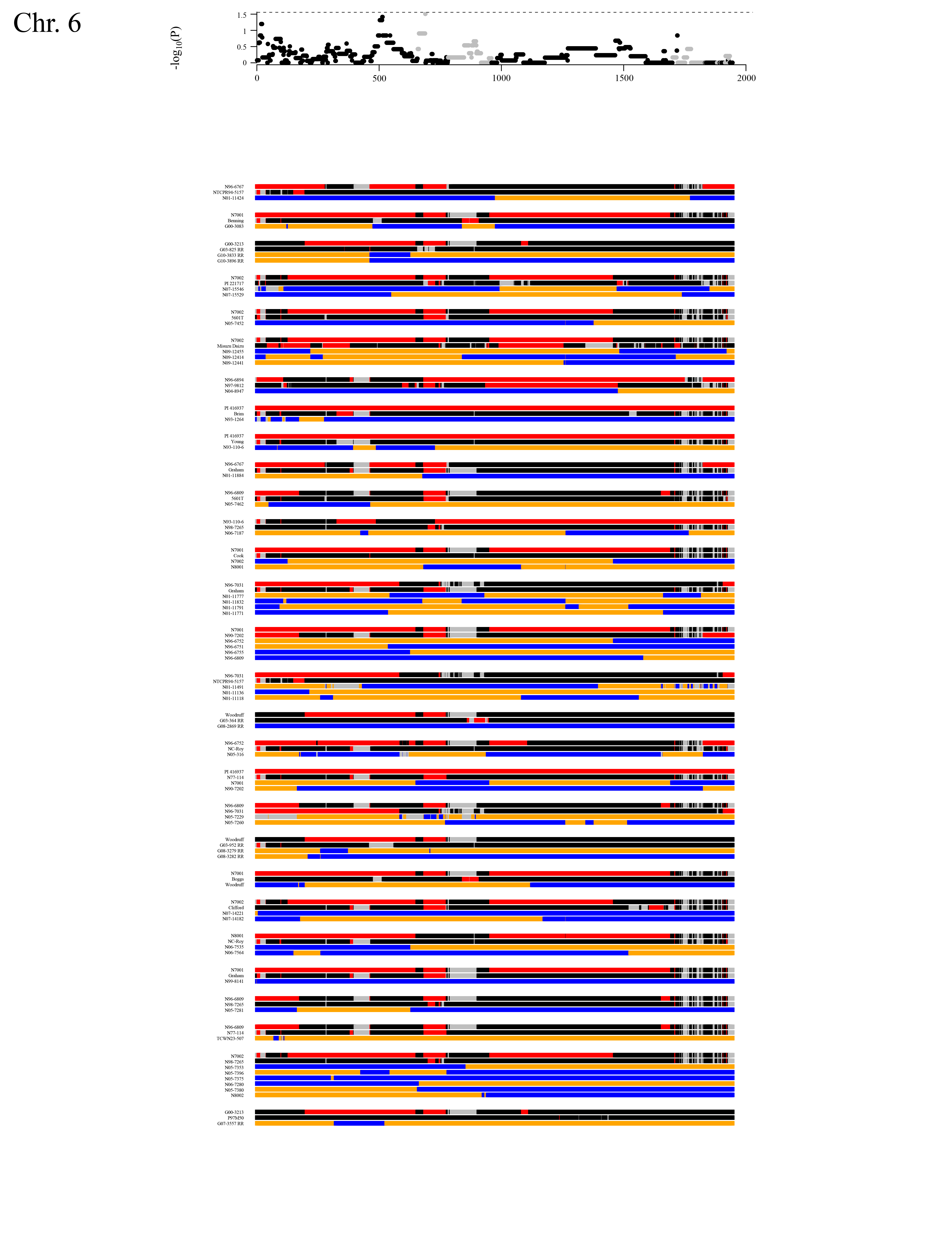

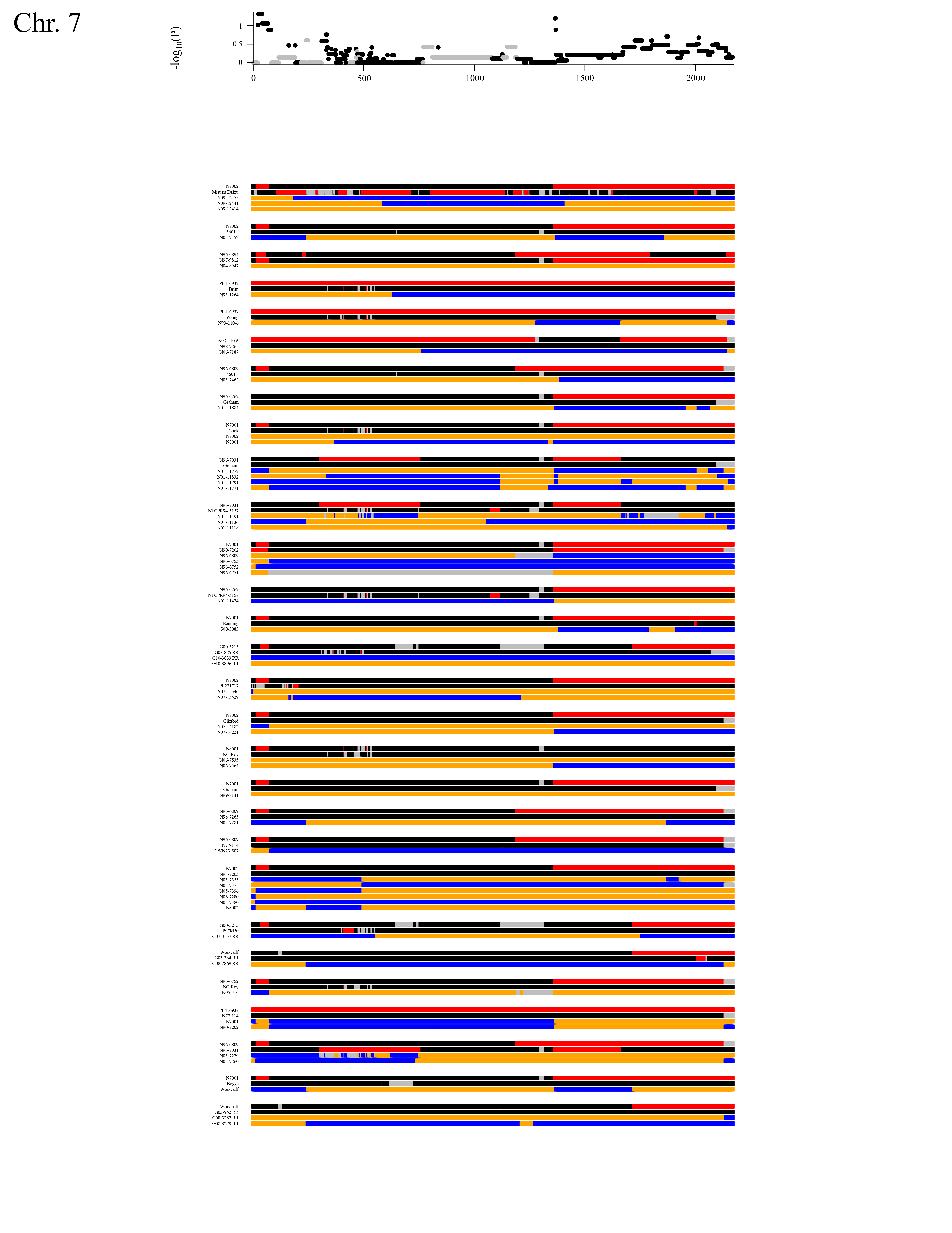

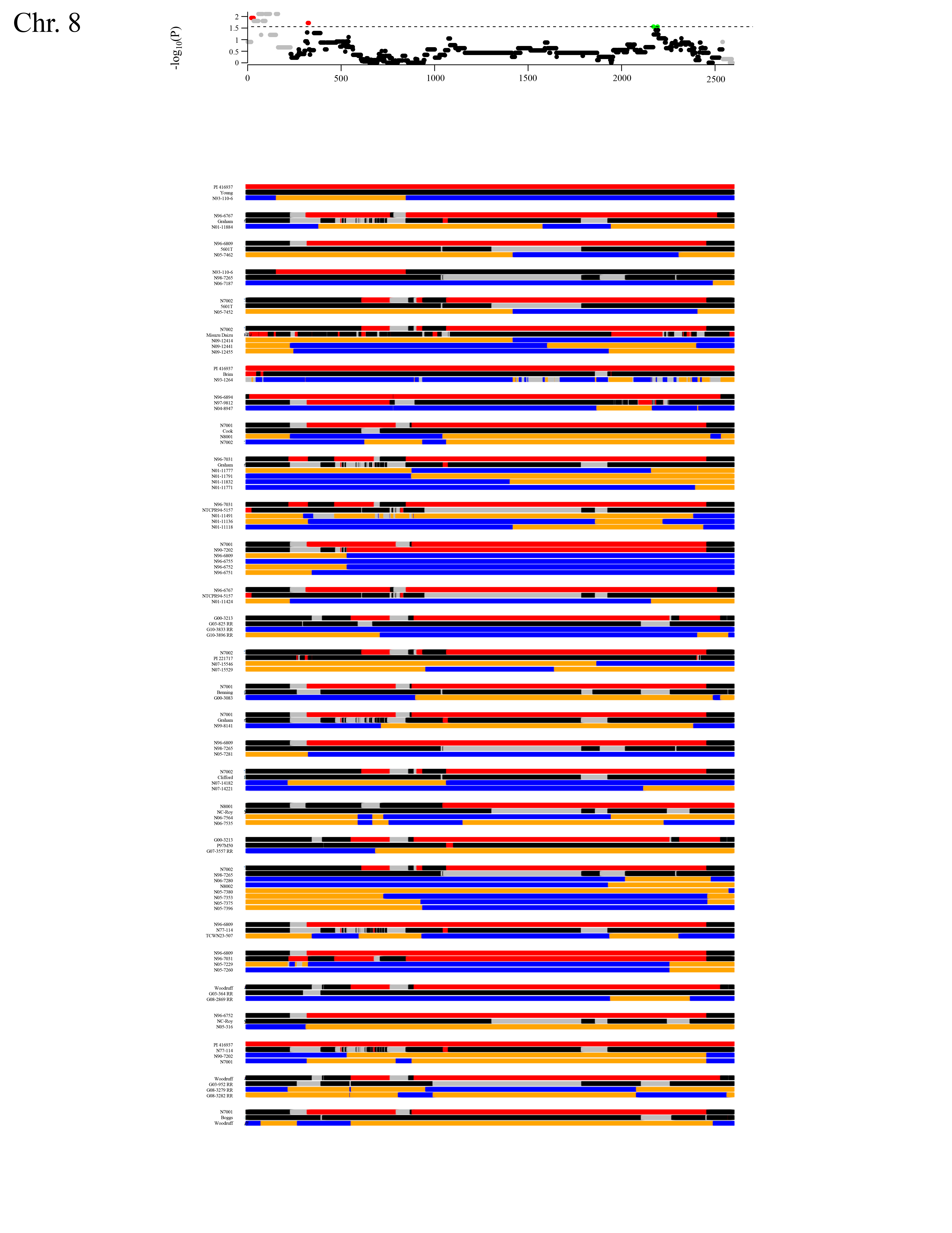

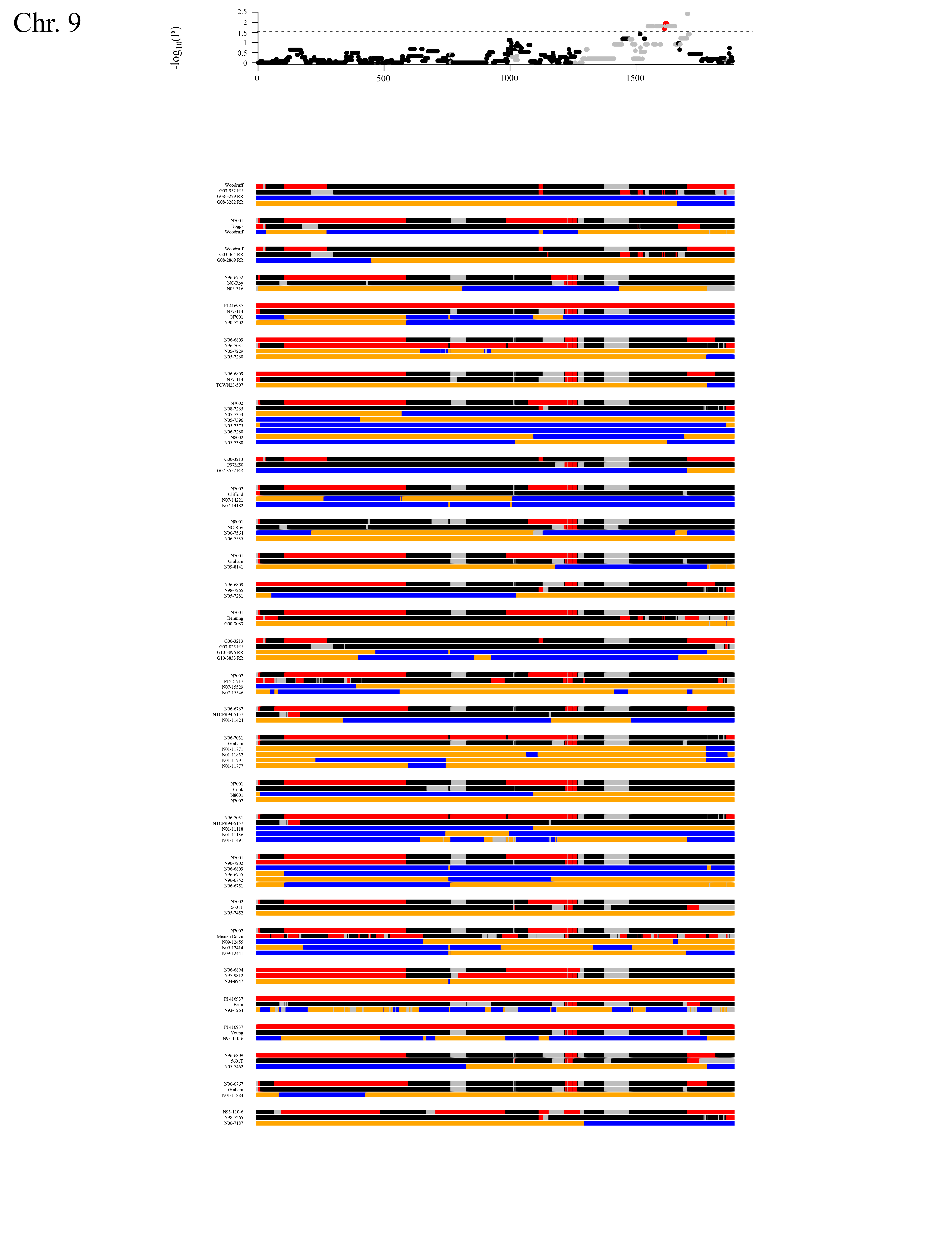

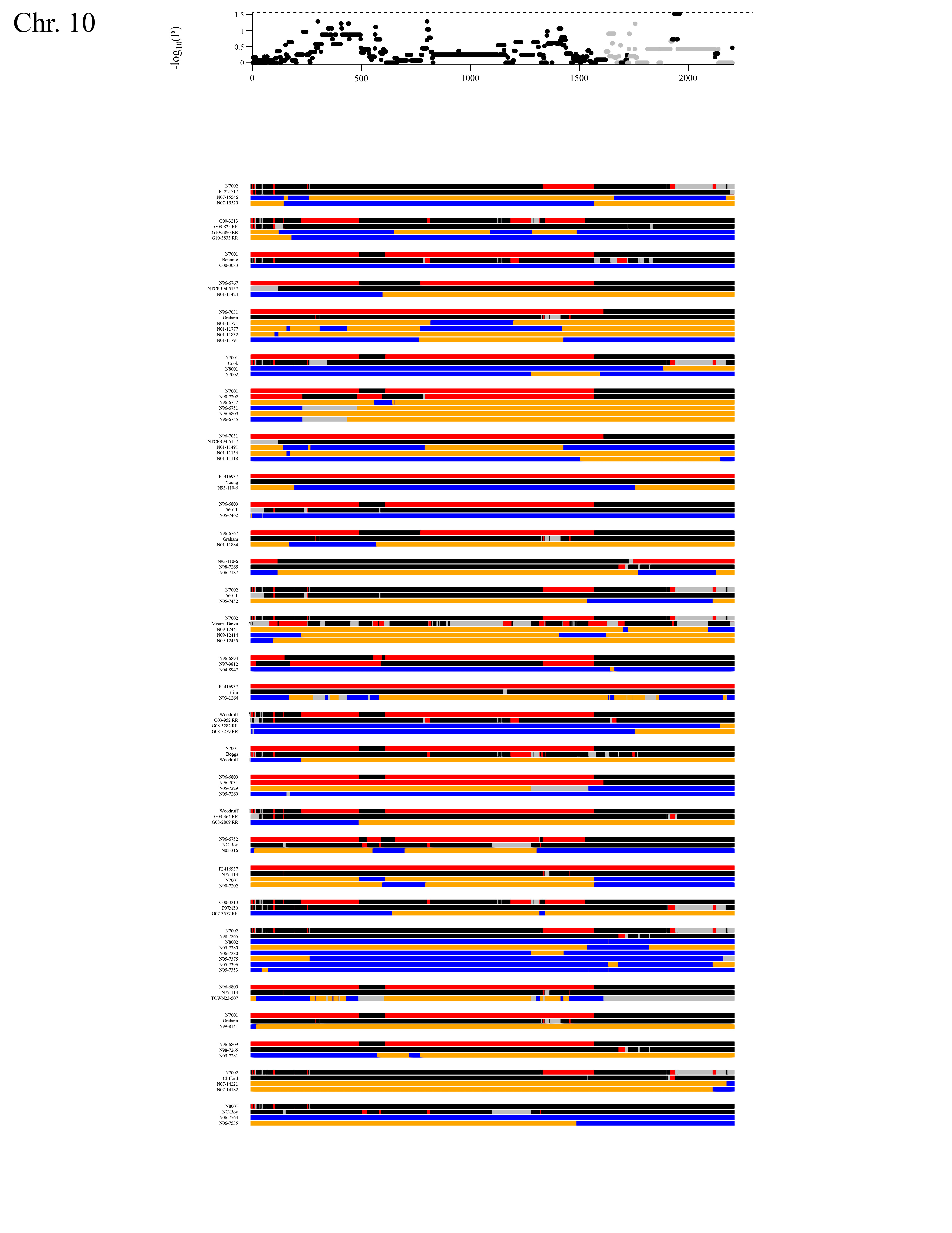

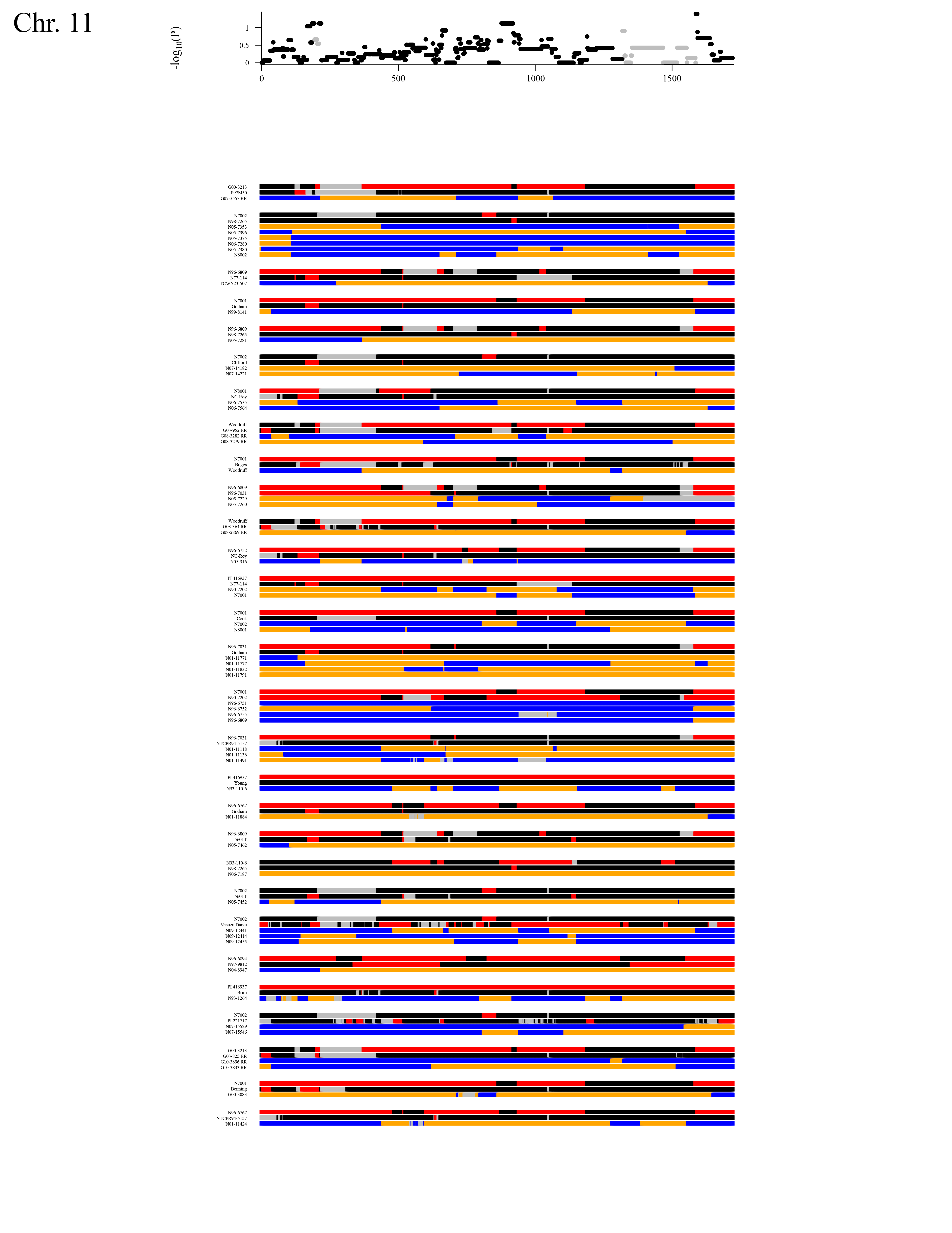

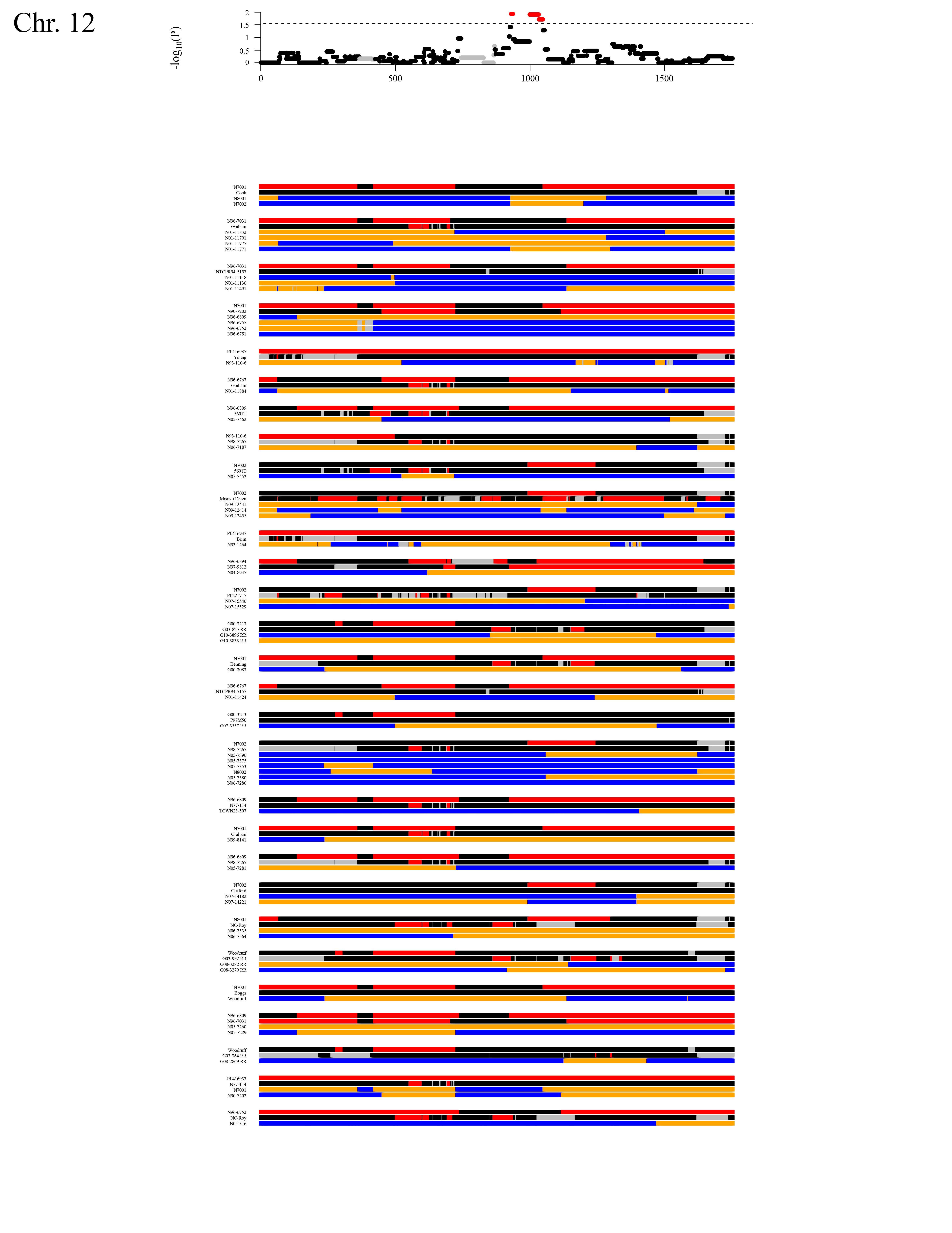

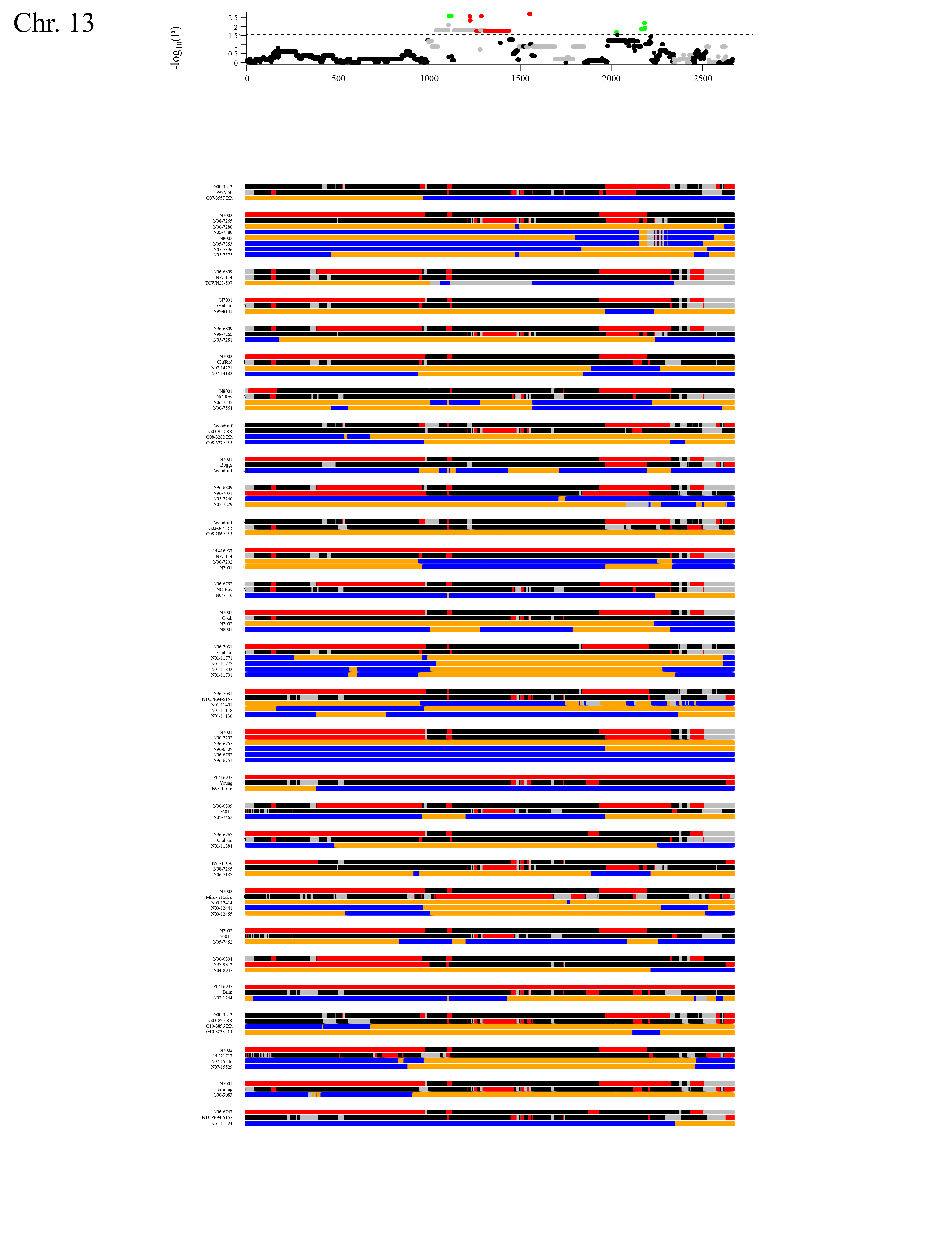

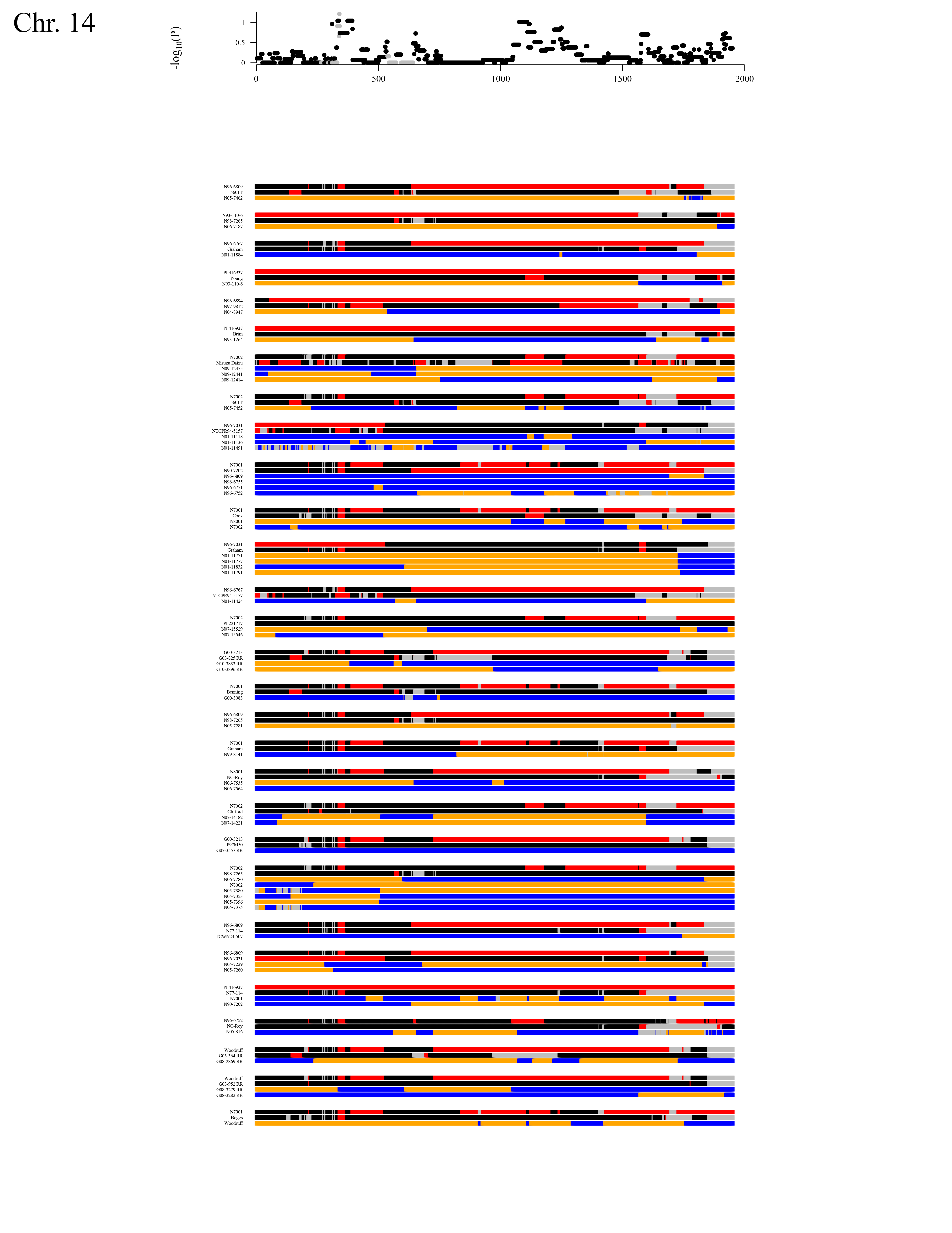

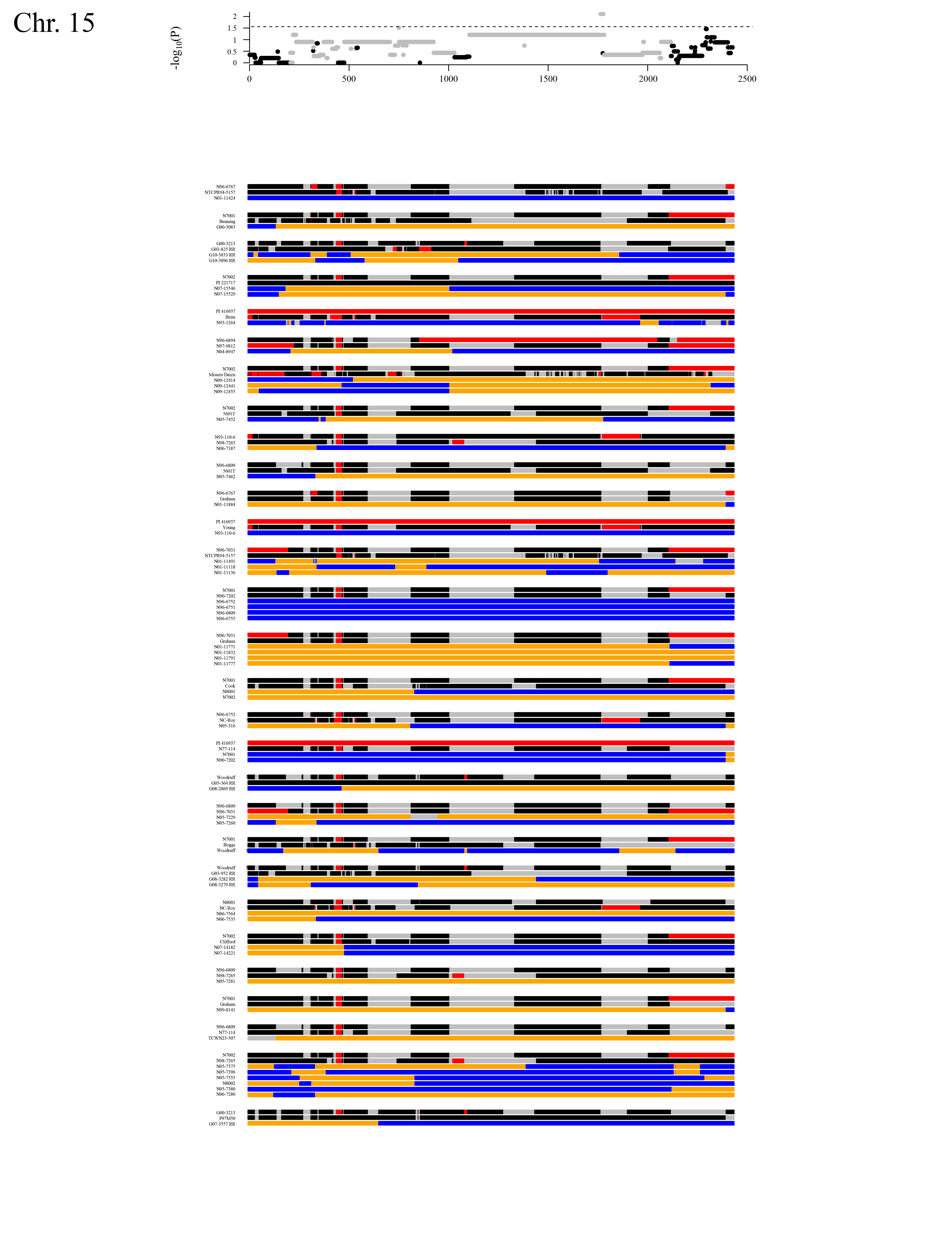

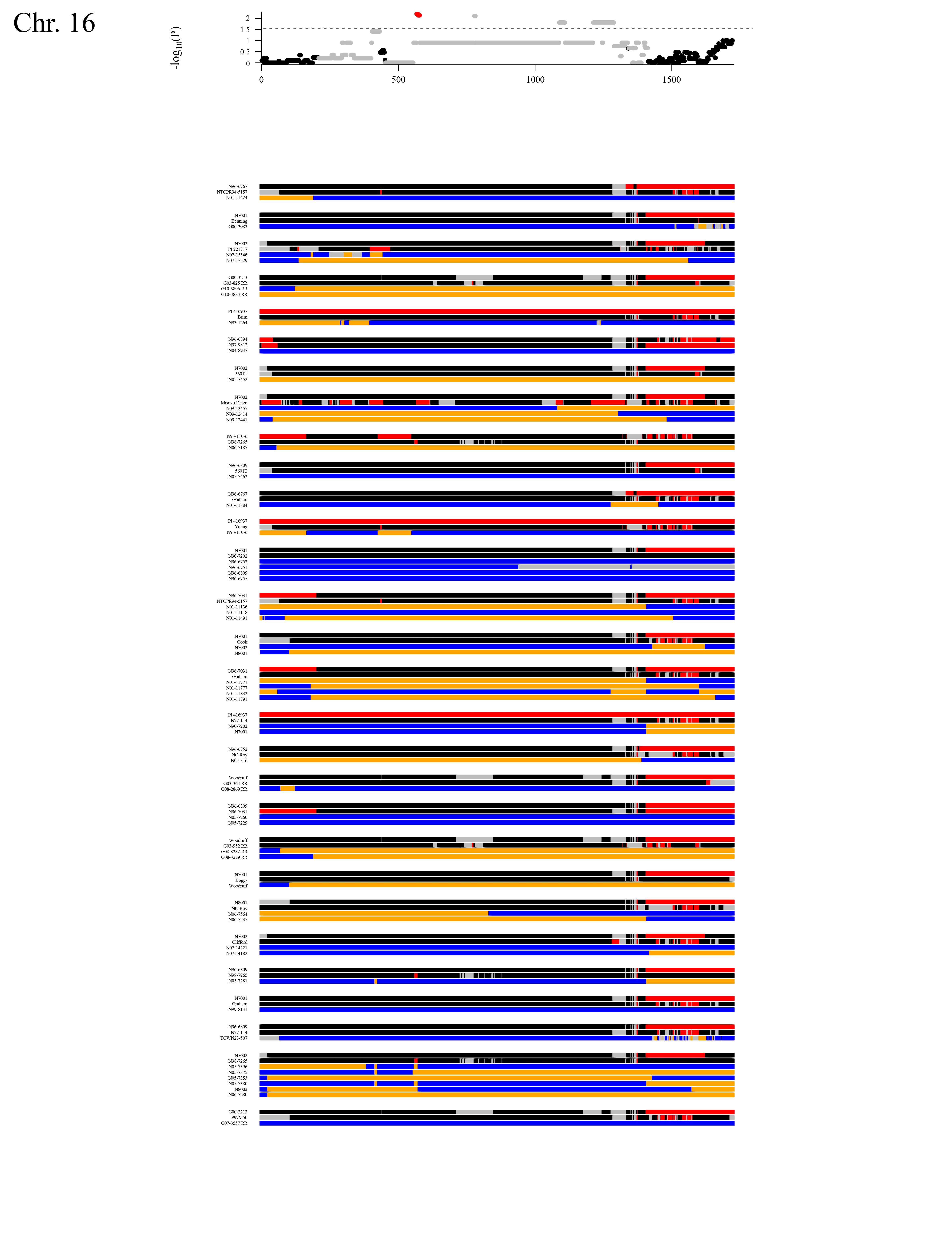

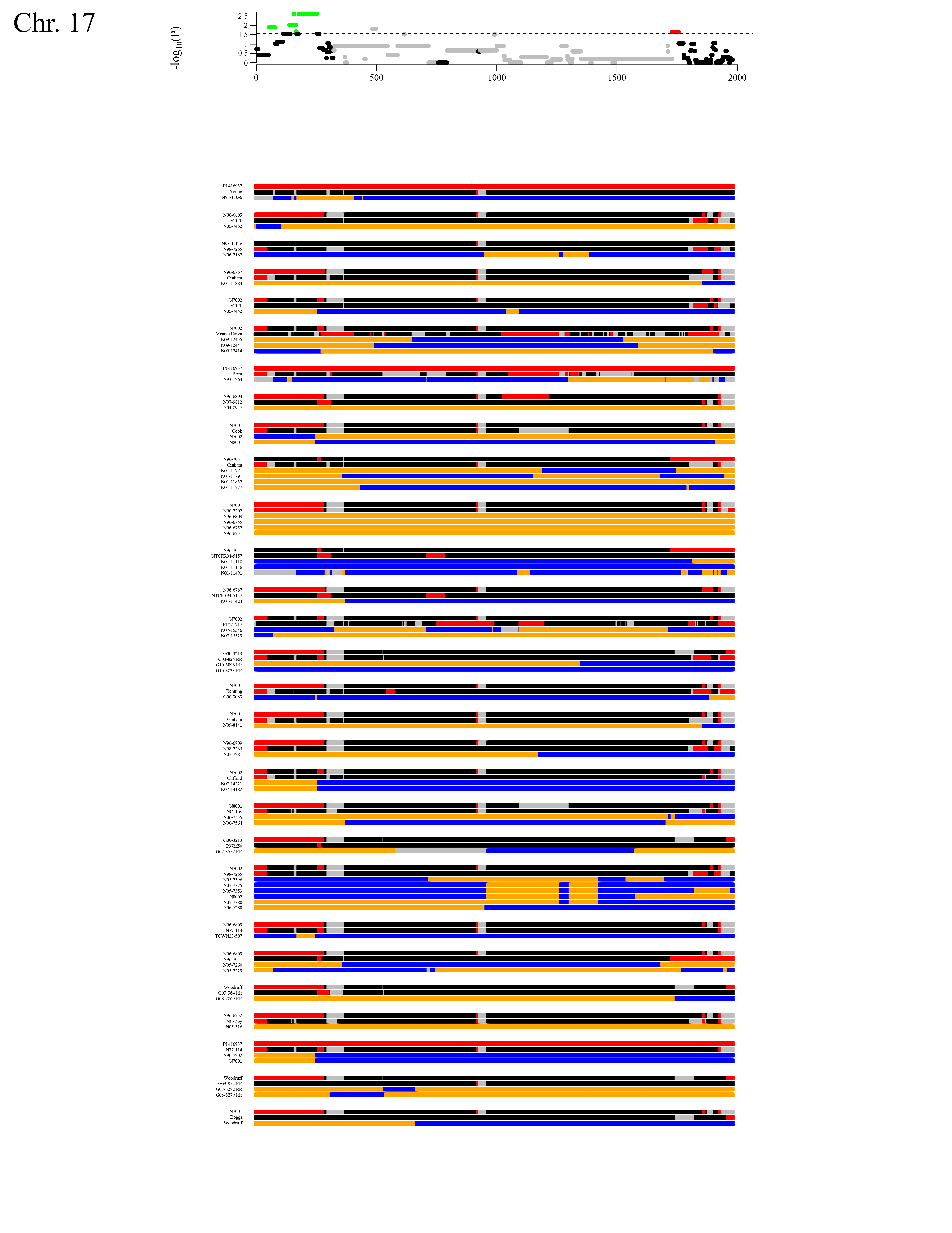

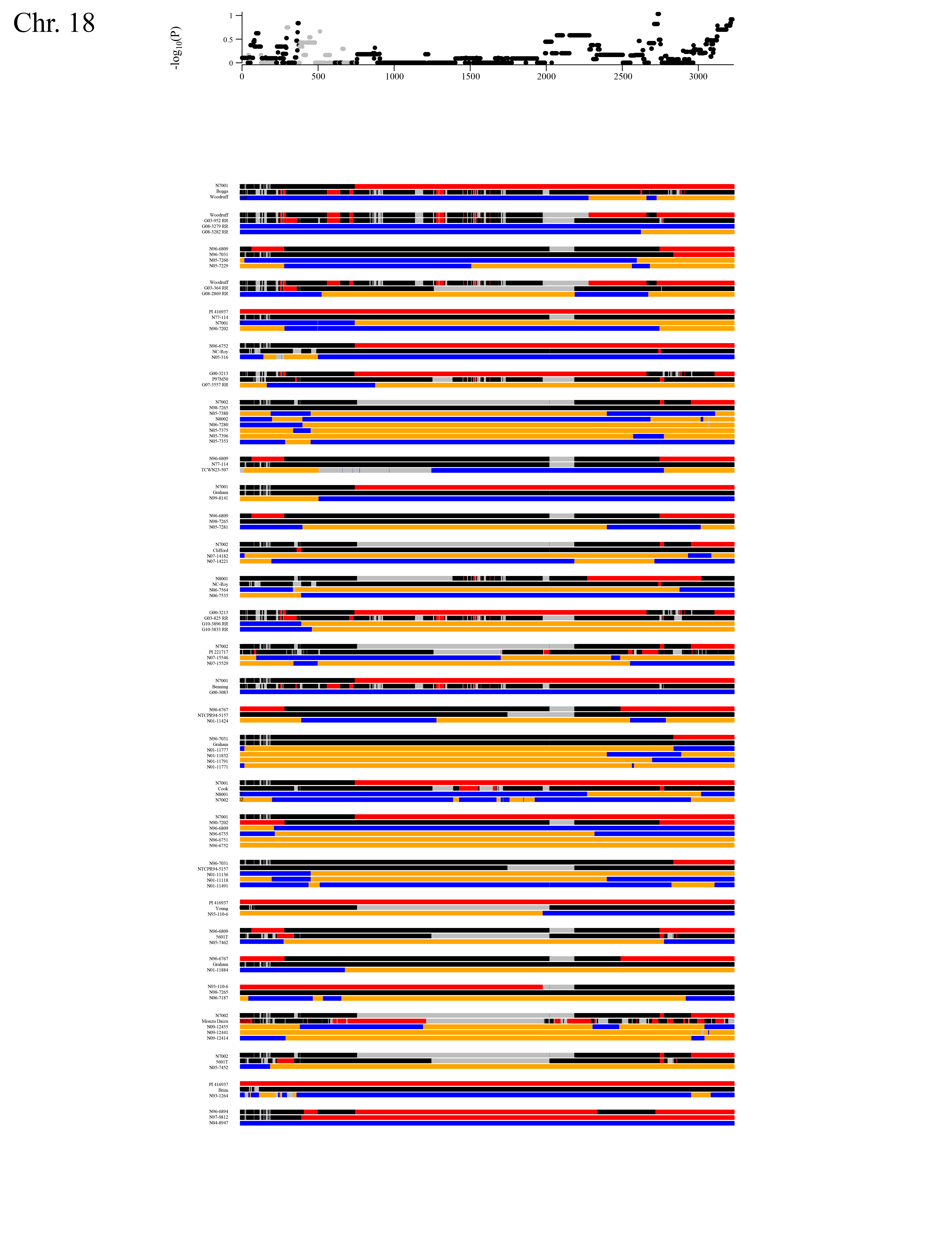

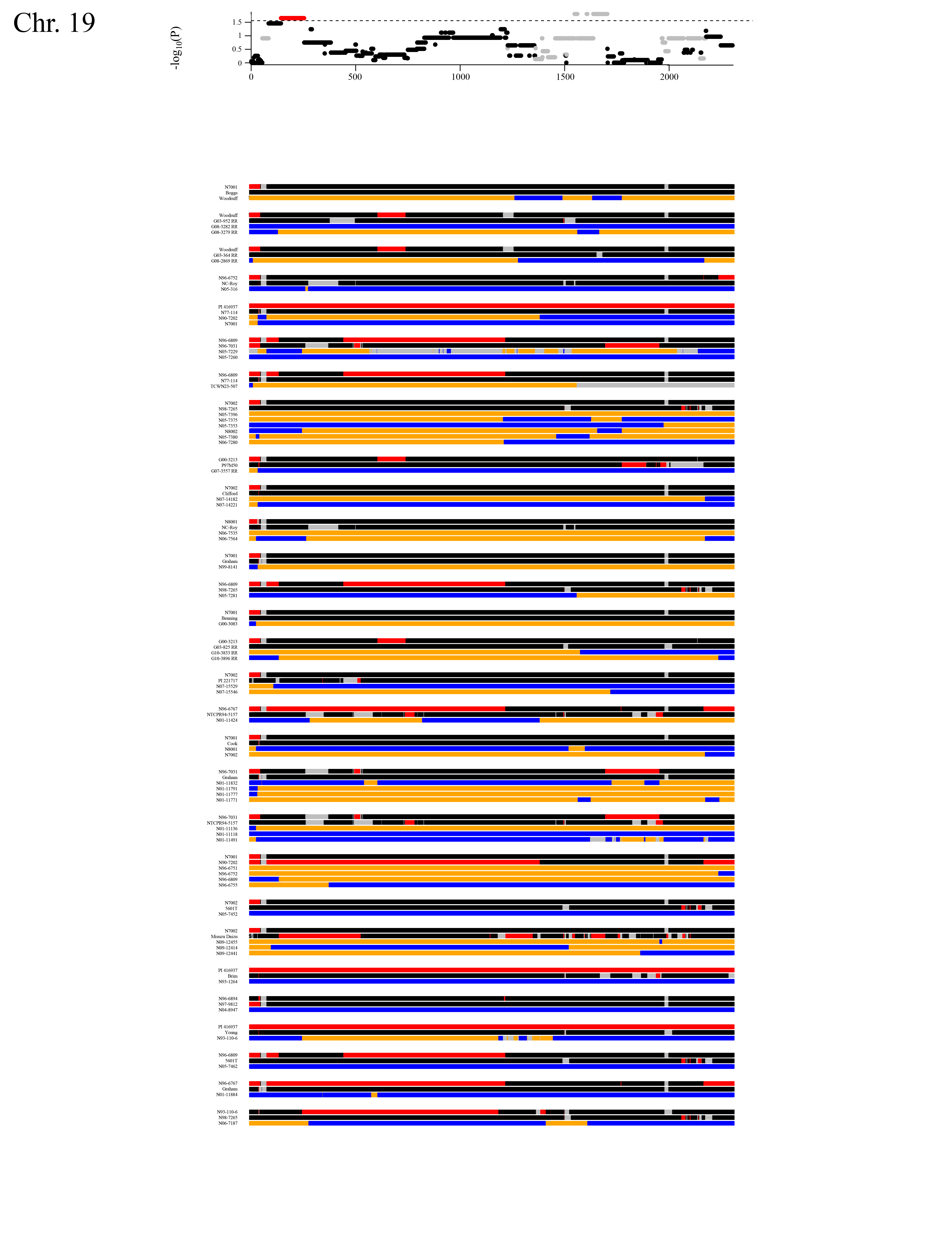

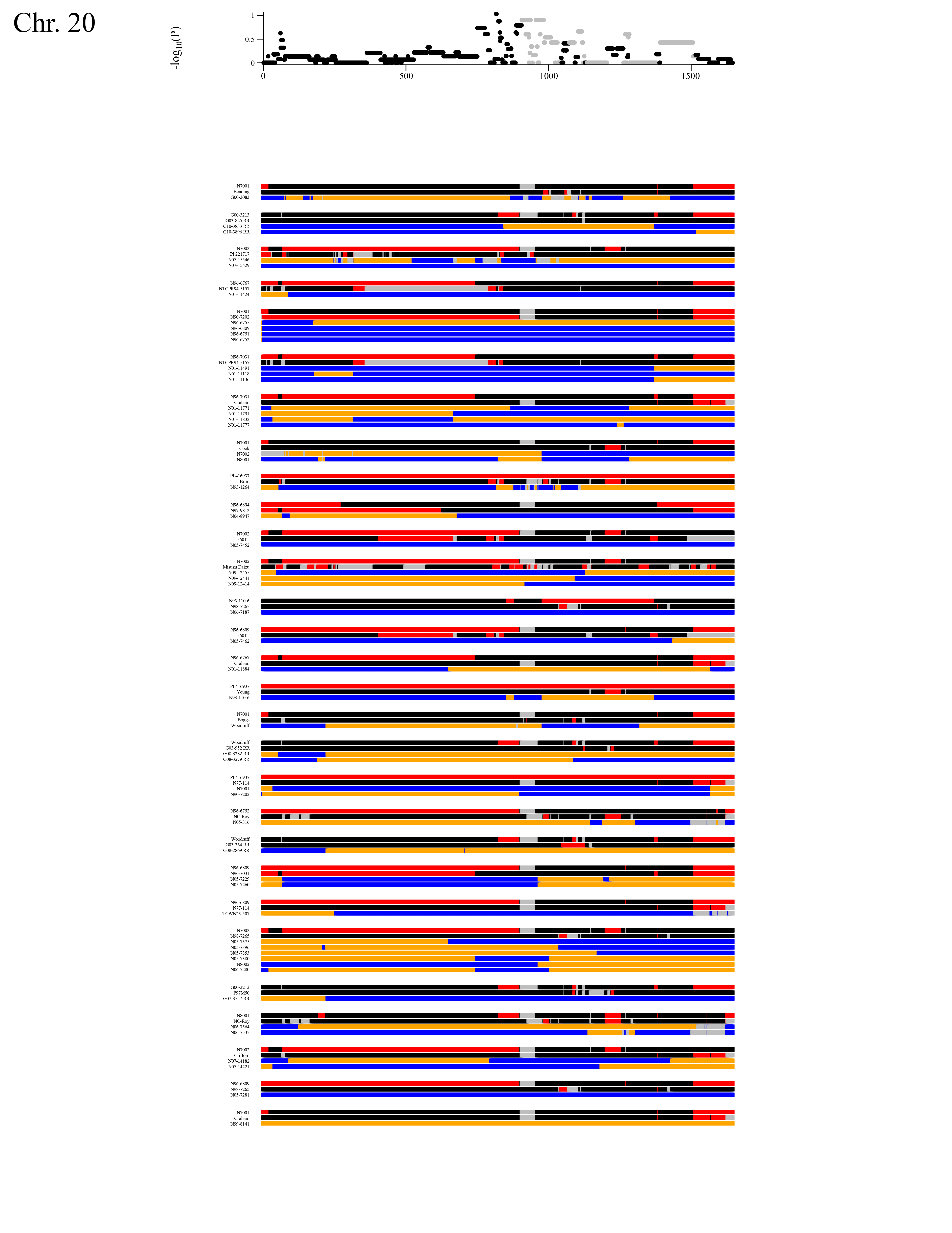

Supplement: S1 File — The top section indicates genomic regions across chromosome 8 under significant positive (green) versus negative selection (red). The statistical threshold was set at a–log10 P-value of 1.56 (Yld1). Gray indicates a locus had less than 10 tests. Black indicates a locus had 10 tests or more but fell below our significance threshold. The bottom portion of the figure displays the chromosomal inheritance for each trio broken up by unique crosses. The top two lines for each cross are the parents while the bottom lines are the high yielding PI 416937-derived progeny from each cross. For the parents, red indicates a chromosomal region inherited from PI 416937. Black indicates a chromosomal region inherited from a major southern ancestor [53]. For the progeny, orange indicates chromosomal inheritance from the top parent and blue indicates inheritance from the bottom parent. Gray for both parents and progeny indicates chromosomal inheritance was ambiguous. (DOCX) [file pone.0235434.s001.docx]

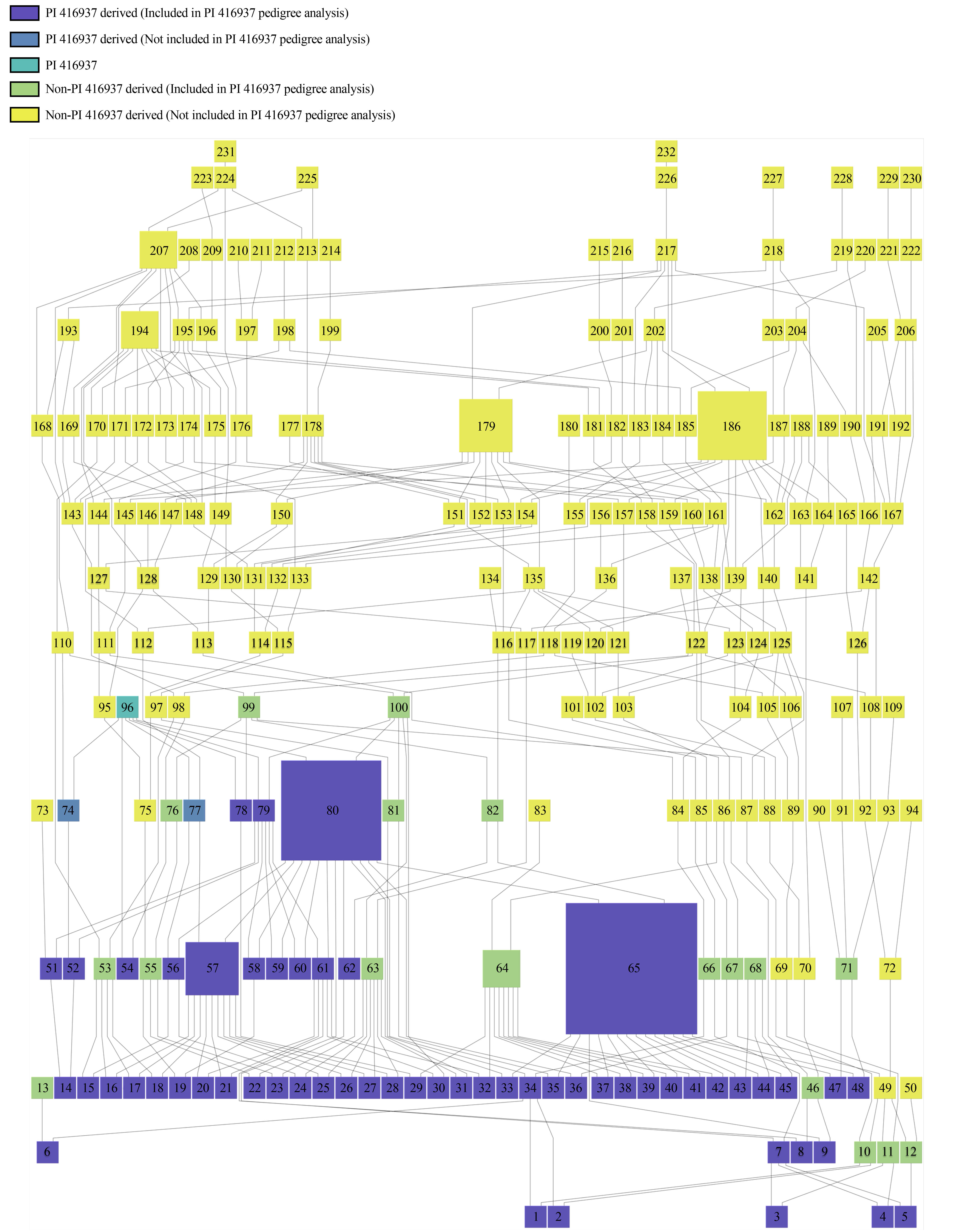

Supplement: S1 Fig — Each genotype is represented by a square with lines connecting parents to progeny. Purple indicates that genotypes were derived from PI 416937 and included in the pedigree analysis. Blue corresponds to that genotypes derived from PI 416937 that were not included in the pedigree analysis. Turquoise indicates PI 416937. Green corresponds to genotypes that were not derived from PI 416937, but included in the pedigree analysis. Yellow indicates that genotypes were not derived from PI 416937 and not included in the pedigree analysis. For breeding lines with greater than 6 progeny, squares vary in size based upon how many direct progeny are derived from a particular line. Genotypes were coded in S1 Fig as numbers which were defined in S3 Table. (TIF) [file pone.0235434.s004.tif]

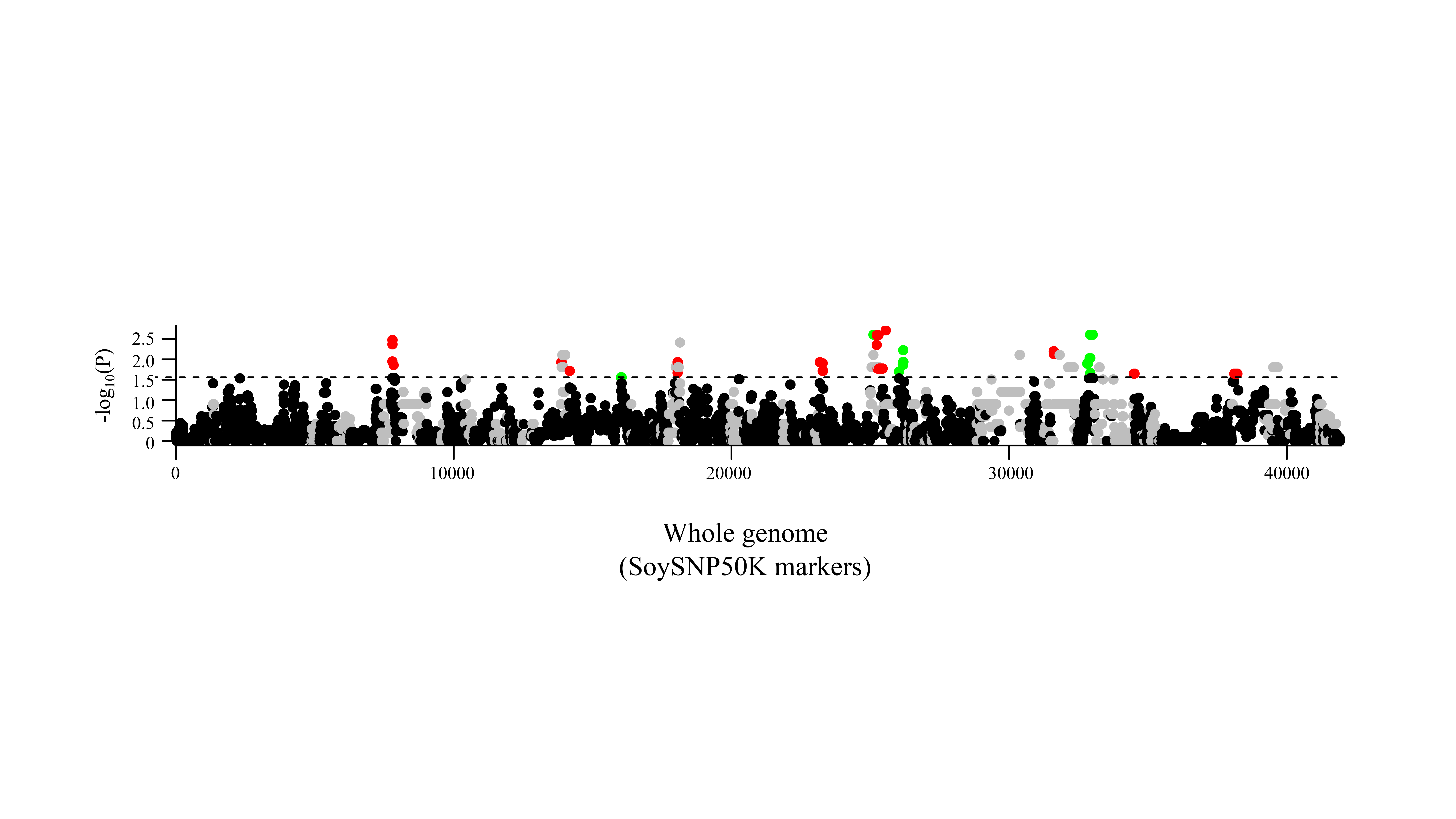

Supplement: S4 Fig — Genomic regions from PI 416937 under positive (green) and negative (red) selection across the whole genome. The x-axis displays marker positions from SoySNP50K Infinium BeadChips. The statistical threshold was set at a–log10 P-value of 1.56 (Yld1). Gray indicates a locus had less than 10 trios testing a PI 416937 allele against an alternative allele. Black indicates a locus had 10 trios or more but fell below our significance threshold. (TIF) [file pone.0235434.s007.tif]

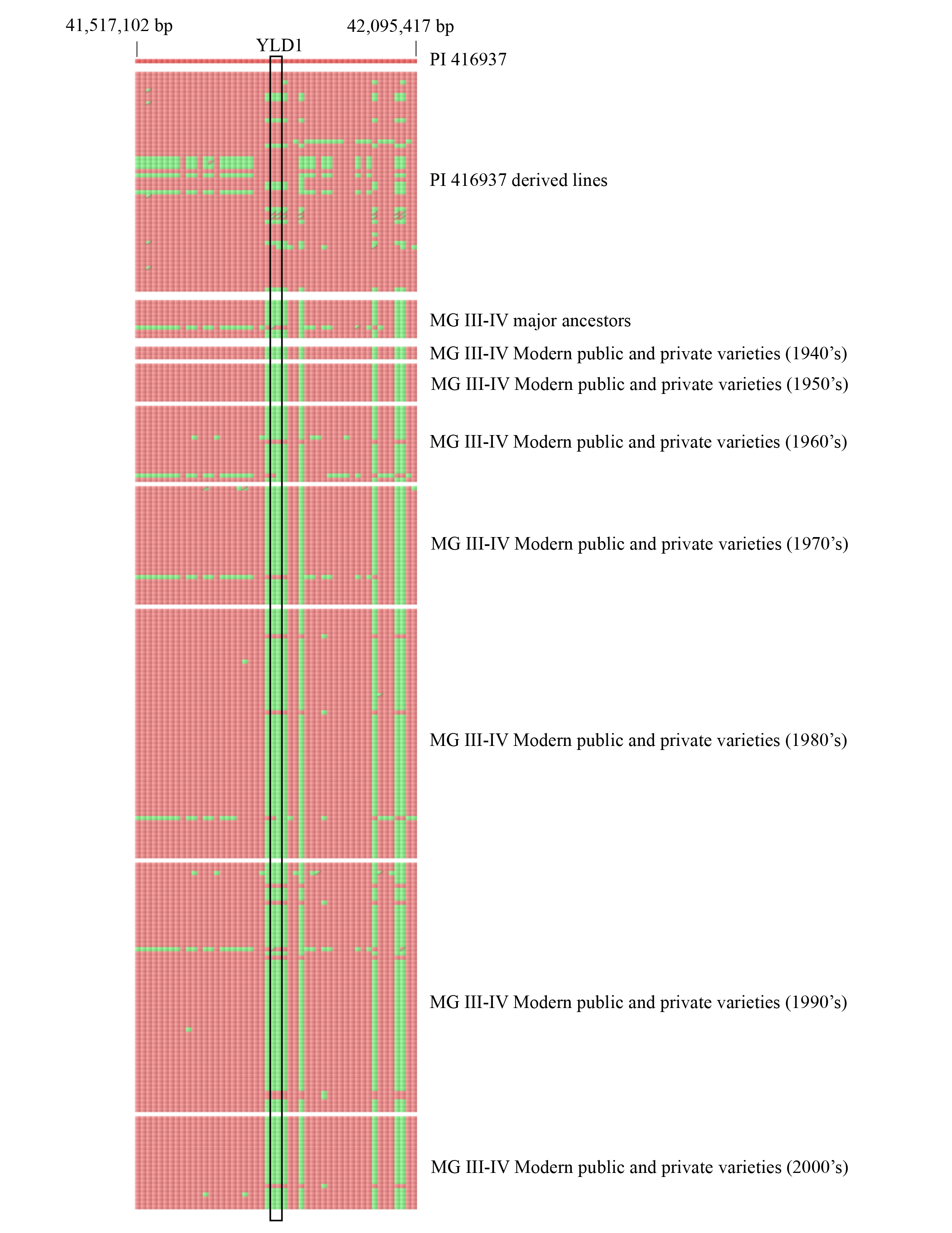

Supplement: S5 Fig — The top line indicates the PI 416937 haplotype. The second, third, and fourth sections display haplotypes for all 52 high yielding PI 416937-derived lines used in our pedigree analysis, the major ancestors of MG III-IV according to Vaughn and Li (2016), and modern public and private cultivars bred for MG III-IV by decade of release, respectively. Red blocks are alleles identical to PI 416937 while green blocks are the alternative alleles for each locus. The Yld1 locus is highlighted with a black outline. Visualization was performed using Flapjack–graphical genotype visualization [70]. (TIF) [file pone.0235434.s008.tif]

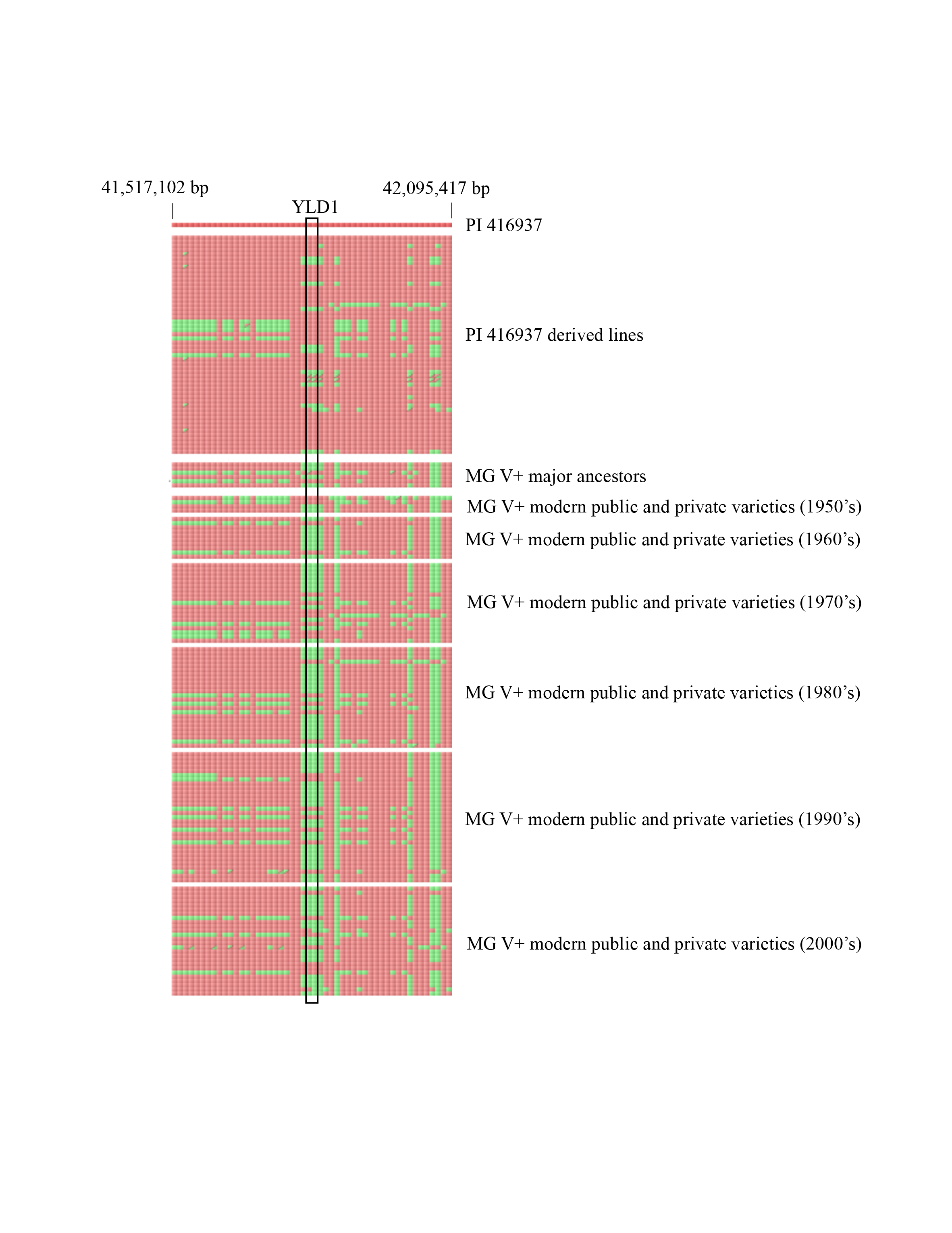

Supplement: S6 Fig — The top line indicates the PI 416937 haplotype. The second, third, and fourth sections display haplotypes for all 52 high yielding PI 416937-derived lines used in our pedigree analysis, the major ancestors of MG V+ according to Vaughn and Li (2016), and modern public and private cultivars bred for MG V+ by decade of release, respectively. Red blocks are alleles identical to PI 416937 while green blocks are the alternative alleles for each locus. The Yld1 locus is highlighted with a black outline. Visualization was performed using Flapjack–graphical genotype visualization [70]. (TIF) [file pone.0235434.s009.tif]
