# Supplementary material for: Characterizing the impact of an exotic soybean line on elite cultivar development
Source: PLoS One. 2020 Jul 10;15(7):e0235434. doi: 10.1371/journal.pone.0235434 (PMC7351202; doi:10.1371/journal.pone.0235434)
Supplement: S1 Table — (DOCX) [file pone.0235434.s010.docx]

S1 Table. Description of RIL populations.

| Population | N | Pedigree |
| --- | --- | --- |
| RIL-1 | 84 | AU02-3104 × G00-3213R2^a^ |
| RIL-2 | 84 | G93-2225 × G09PR-54329R2^a^ |
| RIL-3 | 84 | G10PR-56248R2^a^ × G10PR-56389R2^a^ |
| RIL-4 | 84 | G10PR-10 × G10PR-56389R2^a^ |
| RIL-5 | 150 | G00-3213^a^ × LG04-6000^b^ |

^a^ Parent with PI 416937 in pedigree.

^b^ Nelson RL, Johnson EOC. Registration of the high-yielding soybean germplasm line LG04-6000. J Plant Reg. 2012; 6: 1-4.
