# Supplementary material for: Characterizing the impact of an exotic soybean line on elite cultivar development
Source: PLoS One. 2020 Jul 10;15(7):e0235434. doi: 10.1371/journal.pone.0235434 (PMC7351202; doi:10.1371/journal.pone.0235434)
Supplement: S2 Table — (DOCX) [file pone.0235434.s011.docx]

S2 Table. List of genotypes used in cladograms.

| Name | Alias | PI number | Description | MG | PI 416937 pedigree analysis (Y/N) |
| --- | --- | --- | --- | --- | --- |
| 5601T |  | PI 630984 | Select southern lines | V | Y |
| Benning |  | PI 595645 | Select southern lines | VII | Y |
| Boggs |  | PI 602597 | Select southern lines | VI | Y |
| Brim |  | PI 548986 | Select southern lines | VI | Y |
| Clifford |  | PI 596414 | Select southern lines | V | Y |
| Cook |  | PI 553045 | Select southern lines | VIII | Y |
| G03-364RR |  |  | Select southern lines | VII | Y |
| G03-825RR |  |  | Select southern lines | VIII | Y |
| G03-952RR |  |  | Select southern lines | VIII | Y |
| Graham |  | PI 594922 | Select southern lines | V | Y |
| Misuzu Daizu |  | PI 423912 | Select southern lines | V | Y |
| N77-114 |  |  | Select southern lines | VI | Y |
| N98-7265 |  |  | Select southern lines | V | Y |
| NC-Roy |  | PI 617045 | Select southern lines | VI | Y |
| NTCPR94-5157 |  |  | Select southern lines | VI | Y |
| P97M50 |  |  | Select southern lines | VII | Y |
| Young |  | PI 508266 | Select southern lines | VI | Y |
| G00-3083 |  |  | PI 416937-derived lines | VIII | Y |
| G00-3213 |  |  | PI 416937-derived lines | VII | Y |
| G07-3557RR |  |  | PI 416937-derived lines | VIII | Y |
| G08-2869RR |  |  | PI 416937-derived lines | VIII | Y |
| G08-3279RR |  |  | PI 416937-derived lines | VIII | Y |
| G08-3282RR |  |  | PI 416937-derived lines | VIII | Y |
| G10-3833RR |  |  | PI 416937-derived lines | VII | Y |
| G10-3896RR |  |  | PI 416937-derived lines | VIII | Y |
| N01-11118 |  |  | PI 416937-derived lines | VII | Y |
| N01-11136 |  |  | PI 416937-derived lines | VII | Y |
| N01-11424 |  |  | PI 416937-derived lines | VIII | Y |
| N01-11491 |  |  | PI 416937-derived lines | VII | Y |
| N01-11771 |  |  | PI 416937-derived lines | VII | Y |
| N01-11777 |  |  | PI 416937-derived lines | VII | Y |
| N01-11791 |  |  | PI 416937-derived lines | VII | Y |
| N01-11832 |  |  | PI 416937-derived lines | VIII | Y |
| N01-11884 |  |  | PI 416937-derived lines | VII | Y |
| N04-8947 |  |  | PI 416937-derived lines | VII | Y |
| N05-316 |  |  | PI 416937-derived lines | VI | Y |
| N05-7229 |  |  | PI 416937-derived lines | VII | Y |
| N05-7260 |  |  | PI 416937-derived lines | VII | Y |
| N05-7281 |  |  | PI 416937-derived lines | VII | Y |
| N05-7353 |  |  | PI 416937-derived lines | VI | Y |
| N05-7375 |  |  | PI 416937-derived lines | VI | Y |
| N05-7380 |  |  | PI 416937-derived lines | VII | Y |
| N05-7396 |  |  | PI 416937-derived lines | VII | Y |
| N05-7452 |  |  | PI 416937-derived lines | VII | Y |
| N05-7462 |  |  | PI 416937-derived lines | VII | Y |
| N06-7187 |  |  | PI 416937-derived lines | VIII | Y |
| N06-7280 |  |  | PI 416937-derived lines | VI | Y |
| N06-7535 |  |  | PI 416937-derived lines | VII | Y |
| N06-7564 |  |  | PI 416937-derived lines | VII | Y |
| N07-14182 |  |  | PI 416937-derived lines | VI | Y |
| N07-14221 |  |  | PI 416937-derived lines | V | Y |
| N07-15529 |  |  | PI 416937-derived lines | VII | Y |
| N07-15546 |  |  | PI 416937-derived lines | VI | Y |
| N09-12414 |  |  | PI 416937-derived lines | VII | Y |
| N09-12441 |  |  | PI 416937-derived lines | VII | Y |
| N09-12455 |  |  | PI 416937-derived lines | VII | Y |
| N7001 | N90-7199 | PI 615694 | PI 416937-derived lines | VII | Y |
| N7002 | N97-9658 | PI 647085 | PI 416937-derived lines | VII | Y |
| N8001 | N97-9612 | PI 647086 | PI 416937-derived lines | VII | Y |
| N8002 | N05-7432 | PI 676972 | PI 416937-derived lines | VII | Y |
| N90-7202 |  |  | PI 416937-derived lines | VII | Y |
| N93-110-6 |  |  | PI 416937-derived lines | VI | Y |
| N93-1264 |  |  | PI 416937-derived lines | V | Y |
| N96-6751 |  |  | PI 416937-derived lines | VII | Y |
| N96-6752 |  |  | PI 416937-derived lines | VIII | Y |
| N96-6755 |  |  | PI 416937-derived lines | VI | Y |
| N96-6767 |  |  | PI 416937-derived lines | VIII | Y |
| N96-6809 |  |  | PI 416937-derived lines | VII | Y |
| N96-6894 |  |  | PI 416937-derived lines | VIII | Y |
| N96-7031 |  |  | PI 416937-derived lines | VIII | Y |
| N97-9812 |  |  | PI 416937-derived lines | VI | Y |
| N99-8141 |  |  | PI 416937-derived lines | V | Y |
| TCWN23-507 |  |  | PI 416937-derived lines | VI | Y |
| Woodruff | G00-3209 |  | PI 416937-derived lines | VII | Y |
| 7499 |  | PI 611112 | Public variety (2000's) | IV | N |
| 5002T |  | PI 634193 | Public variety (2000's) | V | N |
| A-100 |  | PI 548668 | Private variety (1960's) | I | N |
| A.K. (Harrow) |  | PI 548298 | N.A. soybean ancestor | III | N |
| A1214 |  | PI 556776 | Private variety (1980's) | I | N |
| A1525 |  | PI 556779 | Private variety (1980's) | I | N |
| A1662 |  | PI 550740 | Private variety (1990's) | I | N |
| A1937 |  | PI 556637 | Private variety (1980's) | I | N |
| A2187 |  | PI 556783 | Private variety (1980's) | II | N |
| A2234 |  | PI 556850 | Private variety (1980's) | II | N |
| A2242 |  | PI 561201 | Private variety (1990's) | II | N |
| A2427 |  | PI 540452 | Private variety (1990's) | II | N |
| A2506 |  | PI 561717 | Private variety (1990's) | II | N |
| A2522 |  | PI 556729 | Private variety (1980's) | II | N |
| A2543 |  | PI 556929 | Private variety (1990's) | II | N |
| A2943 |  | PI 556689 | Private variety (1980's) | III | N |
| A3127 |  | PI 556511 | Private variety (1970's) | III | N |
| A3205 |  | PI 556816 | Private variety (1980's) | III | N |
| A3307 |  | PI 556781 | Private variety (1980's) | III | N |
| A3313 |  | PI 591561 | Private variety (1990's) | III | N |
| A3322 |  | PI 556928 | Private variety (1980's) | III | N |
| A3415 |  | PI 556859 | Private variety (1980's) | III | N |
| A3427 |  | PI 556778 | Private variety (1980's) | III | N |
| A3510 |  | PI 568245 | Private variety (1990's) | III | N |
| A3659 |  | PI 556572 | Private variety (1980's) | III | N |
| A3733 |  | PI 556814 | Private variety (1980's) | III | N |
| A3803 |  | PI 556780 | Private variety (1980's) | III | N |
| A3935 |  | PI 556857 | Private variety (1980's) | III | N |
| A3966 |  | PI 556687 | Private variety (1980's) | III | N |
| A4415 |  | PI 568254 | Private variety (1990's) | IV | N |
| A4715 |  | PI 539936 | Private variety (1990's) | IV | N |
| A5560 |  | PI 561218 | Private variety (1990's) | V | N |
| A5848 |  | PI 594386 | Private variety (1990's) | V | N |
| A6785 |  | PI 527704 | Private variety (1980's) | VI | N |
| Acme |  | PI 548498 | Public variety (1950's) | 0 | N |
| Ada |  | PI 548499 | Public variety (1970's) | 0 | N |
| Adams |  | PI 548502 | Public variety (1940's) | III | N |
| Adelphia |  | PI 548503 | Public variety (1960's) | III | N |
| Agassiz |  | PI 562372 | Public variety (1990's) | 0 | N |
| Alamo |  | PI 548969 | Public variety (1970's) | IX | N |
| Alpha |  | PI 564524 | Public variety (1990's) | I | N |
| Altona |  | PI 548504 | Public variety (1960's) | 0 | N |
| Amcor |  | PI 548505 | Public variety (1970's) | II | N |
| Amcor 89 |  | PI 546375 | Public variety (1980's) | II | N |
| Amsoy |  | PI 548506 | Public variety (1960's) | II | N |
| Amsoy 71 |  | PI 548507 | Public variety (1970's) | II | N |
| Anand |  | PI 614732 | Public variety (2000's) | V | N |
| Anoka |  | PI 548508 | Public variety (1970's) | I | N |
| AP 200 |  | PI 548692 | Private variety (1980's) | II | N |
| AP 26 |  | PI 548691 | Private variety (1970's) | III | N |
| Apex |  | PI 632401 | Public variety (2000's) | III | N |
| Apollo |  | PI 602059 | Public variety (1990's) | II | N |
| Archer |  | PI 546487 | Public variety (1990's) | I | N |
| Arksoy |  | PI 548438 | N.A. soybean ancestor | VI | N |
| Asmara |  | PI 633049 | Public variety (2000's) | VI | N |
| Athow |  | PI 595926 | Public variety (1990's) | III | N |
| Avery |  | PI 518663 | Public variety (1980's) | IV | N |
| B216 |  | PI 548689 | Private variety (1970's) | II | N |
| Bansei |  | PI 548302 | N.A. soybean ancestor | II | N |
| Barnes |  | PI 614831 | Public variety (2000's) | 0 | N |
| Bass |  | PI 548652 | Public variety (1980's) | III | N |
| Bay |  | PI 553043 | Public variety (1970's) | V | N |
| Bedford |  | PI 548974 | Public variety (1970's) | V | N |
| Beeson |  | PI 548510 | Public variety (1960's) | II | N |
| Beeson 80 |  | PI 548511 | Public variety (1970's) | II | N |
| Bell |  | PI 540554 | Public variety (1980's) | I | N |
| Bert |  | PI 557010 | Public variety (1990's) | I | N |
| Bethel |  | PI 548514 | Public variety (1960's) | IV | N |
| Bicentennial |  | PI 548515 | Public variety (1980's) | 0 | N |
| Bienville |  | PI 567788 | Public variety (1950's) | VIII | N |
| Bilomi No.3 |  | PI 240664 | N.A. soybean ancestor | I0 | N |
| Bolivar |  | PI 612146 | Public variety (1990's) | V | N |
| Bonus |  | PI 548517 | Public variety (1970's) | IV | N |
| Bradley |  | PI 556738 | Public variety (1980's) | VI | N |
| Bragg |  | PI 548660 | Public variety (1960's) | VII | N |
| Braxton |  | PI 548659 | Select southern lines | VII | N |
| Brock |  | PI 572241 | Public variety (1990's) | I | N |
| Bronson |  | PI 577798 | Public variety (1990's) | IV | N |
| BSR 101 |  | PI 548519 | Public variety (1980's) | I | N |
| BSR 201 |  | PI 548521 | Public variety (1980's) | II | N |
| BSR 301 |  | PI 548522 | Public variety (1970's) | III | N |
| BSR 302 |  | PI 548525 | Public variety (1980's) | III | N |
| Buckshot 723 |  | PI 543832 | Public variety (1990's) | VII | N |
| Burlison |  | PI 533655 | Public variety (1980's) | II | N |
| Calhoun |  | PI 576440 | Public variety (1990's) | IV | N |
| Calland |  | PI 548527 | Public variety (1960's) | III | N |
| Camp |  | PI 553044 | Public variety (1980's) | V | N |
| Camp-lx2 |  | PI 596540 | Public variety (1990's) | V | N |
| Canatto |  | PI 548648 | Public variety (1980's) | 0 | N |
| Capital |  | PI 548311 | N.A. soybean ancestor | 0 | N |
| Carlin |  | PI 548669 | Private variety (1940's) | IV | N |
| Cartter |  | PI 518675 | Public variety (1980's) | III | N |
| Carver |  | PI 584506 | Public variety (1990's) | VII | N |
| Catoosa |  | PI 618808 | Public variety (2000's) | V | N |
| Caviness |  | PI 615582 | Public variety (2000's) | V | N |
| Celest |  | PI 612608 | Public variety (1970's) | V | N |
| Centennial |  | PI 548975 | Select southern lines | VI | N |
| Century |  | PI 548512 | Public variety (1970's) | II | N |
| Century 84 |  | PI 548529 | Public variety (1980's) | II | N |
| CF 461 |  | PI 590932 | Public variety (1990's) | IV | N |
| CF 492 |  | PI 590931 | Public variety (1990's) | IV | N |
| Chamberlain |  | PI 548635 | Public variety (1980's) | III | N |
| Chapman |  | PI 542710 | Public variety (1990's) | II | N |
| Charleston |  | PI 567902 | Public variety (1990's) | III | N |
| Chico |  | PI 542402 | Public variety (1980's) | 0 | N |
| Chippewa |  | PI 548530 | Public variety (1950's) | I | N |
| Chippewa 64 |  | PI 548531 | Public variety (1960's) | I | N |
| Ciaric |  | PI 570668 | Public variety (1990's) | VI | N |
| Cisne |  | PI 593256 | Public variety (1990's) | IV | N |
| Clark |  | PI 548533 | Public variety (1950's) | IV | N |
| Clark 63 |  | PI 548532 | Public variety (1960's) | IV | N |
| Clay |  | PI 548534 | Public variety (1960's) | 0 | N |
| CN210 |  | PI 518676 | Public variety (1980's) | II | N |
| CN290 |  | PI 518677 | Public variety (1980's) | II | N |
| CNS |  | PI 548445 | N.A. soybean ancestor | VII | N |
| Cobb |  | PI 548664 | Public variety (1970's) | VIII | N |
| Coles |  | PI 548536 | Public variety (1970's) | I | N |
| Colfax |  | PI 573008 | Public variety (1990's) | II | N |
| Columbus |  | PI 548538 | Public variety (1970's) | IV | N |
| Comet |  | PI 548539 | Public variety (1950's) | 0 | N |
| Conrad |  | PI 525453 | Public variety (1980's) | II | N |
| Cordell |  | PI 533605 | Public variety (1980's) | V | N |
| Corsica |  | PI 559931 | Public variety (1990's) | IV | N |
| Corsoy |  | PI 548540 | Public variety (1960's) | II | N |
| Corsoy 79 |  | PI 518669 | Public variety (1970's) | II | N |
| Council |  | PI 587091 | Public variety (1990's) | 0 | N |
| Crawford |  | PI 548541 | Public variety (1970's) | IV | N |
| Crest |  | PI 548544 | Public variety (1950's) | 0 | N |
| Crockett |  | PI 535807 | Public variety (1980's) | VIII | N |
| Croton 3.9 |  | PI 614153 | Public variety (2000's) | III | N |
| Cumberland |  | PI 548542 | Public variety (1970's) | III | N |
| Curtis |  | PI 567790 | Public variety (1950's) | VI | N |
| Custer |  | PI 548546 | Public variety (1960's) | IV | N |
| Cutler |  | PI 548547 | Public variety (1960's) | IV | N |
| Cutler 71 |  | PI 548518 | Public variety (1970's) | IV | N |
| CX291 |  | PI 547094 | Private variety (1990's) | II | N |
| CX298 |  | PI 556888 | Private variety (1980's) | II | N |
| CX326 |  | PI 634757 | Private variety (2000's) | III | N |
| CX329 |  | PI 556931 | Private variety (1990's) | III | N |
| CX335 |  | PI 576160 | Private variety (1990's) | III | N |
| CX345 |  | PI 634758 | Private variety (2000's) | III | N |
| CX394c |  | PI 576161 | Private variety (1990's) | III | N |
| CX411 |  | PI 576166 | Private variety (1990's) | IV | N |
| CX415 |  | PI 634760 | Private variety (2000's) | IV | N |
| CX434 |  | PI 576162 | Private variety (1990's) | IV | N |
| CX458 |  | PI 556889 | Private variety (1980's) | IV | N |
| CX469c |  | PI 556932 | Private variety (1990's) | IV | N |
| Cypress No. 1 |  | PI 548670 | Private variety (1950's) | IV | N |
| Daksoy |  | PI 602896 | Public variety (1990's) | 0 | N |
| Danatto |  | PI 593655 | Public variety (1990's) | 0 | N |
| Darby |  | PI 614154 | Public variety (2000's) | III | N |
| Dare |  | PI 548987 | Public variety (1960's) | V | N |
| Dassel |  | PI 508083 | Public variety (1980's) | 0 | N |
| Dawson |  | PI 542403 | Public variety (1980's) | 0 | N |
| Defiance |  | PI 596407 | Public variety (1990's) | III | N |
| Delmar |  | PI 548548 | Public variety (1960's) | IV | N |
| Delsoy 4210 |  | PI 560206 | Public variety (1990's) | IV | N |
| Delsoy 4500 |  | PI 543793 | Public variety (1980's) | IV | N |
| Delsoy 4710 |  | PI 560207 | Public variety (1990's) | IV | N |
| Delsoy 4900 |  | PI 543794 | Public variety (1980's) | IV | N |
| Delsoy 5500 |  | PI 595765 | Public variety (1990's) | V | N |
| Delsoy 5710 |  | PI 607528 | Public variety (1990's) | V | N |
| Derry |  | PI 601982 | Public variety (1990's) | VI | N |
| Desha |  | PI 633610 | Public variety (2000's) | VI | N |
| DeSoto |  | PI 548549 | Public variety (1970's) | IV | N |
| Dillon |  | PI 592756 | Select southern lines | VI | N |
| Dilworth |  | PI 633608 | Public variety (2000's) | III | N |
| Dimon |  | PI 572244 | Public variety (1990's) | II | N |
| Disoy |  | PI 548550 | Public variety (1960's) | I | N |
| Donegal |  | PI 601983 | Public variety (1990's) | V | N |
| Dortchsoy 31 |  | PI 548695 | Private variety (1990's) | VII | N |
| Dortchsoy 67 |  | PI 548696 | Private variety (1950's) | V | N |
| Douglas |  | PI 548555 | Public variety (1980's) | IV | N |
| Dowling |  | PI 548663 | Public variety (1970's) | VIII | N |
| Dunbar |  | PI 552538 | Public variety (1990's) | III | N |
| Dunfield |  | PI 548318 | N.A. soybean ancestor | III | N |
| Dunn |  | PI 548509 | Public variety (1960's) | I | N |
| Dyer |  | PI 548976 | Public variety (1960's) | V | N |
| Edison |  | PI 542711 | Public variety (1990's) | III | N |
| Egyptian |  | PI 506417 | Public variety (1980's) | IV | N |
| Elf |  | PI 548556 | Public variety (1970's) | III | N |
| Elgin |  | PI 548557 | Public variety (1980's) | II | N |
| Emerald |  | PI 548559 | Public variety (1970's) | IV | N |
| Ennis I |  | PI 548677 | Private variety (1960's) | III | N |
| Epps |  | PI 548977 | Public variety (1980's) | V | N |
| Erie |  | PI 561700 | Public variety (1990's) | II | N |
| Essex |  | PI 548667 | Select southern lines | V | N |
| Evans |  | PI 548560 | Public variety (1970's) | 0 | N |
| Fabulin |  | PI 548671 | Private variety (1950's) | IV | N |
| Faribault |  | PI 583364 | Public variety (1990's) | I | N |
| Fayette |  | PI 518674 | Public variety (1980's) | III | N |
| Felix |  | PI 572245 | Public variety (1990's) | I | N |
| Fiskeby 840-7-3 |  | PI 438477 | N.A. soybean ancestor | 0 | N |
| Fiskeby III |  | PI 438471 | N.A. soybean ancestor | 0 | N |
| Fiskeby V |  | PI 360955A | N.A. soybean ancestor | 0 | N |
| Flambeau |  | PI 548325 | N.A. soybean ancestor | 0 | N |
| Flint |  | PI 595843 | Public variety (1990's) | II | N |
| Flyer |  | PI 534646 | Public variety (1980's) | IV | N |
| Ford |  | PI 548562 | Public variety (1950's) | III | N |
| Forrest |  | PI 548655 | Public variety (1970's) | V | N |
| Foster |  | PI 548970 | Public variety (1980's) | VIII | N |
| Fowler |  | PI 613195 | Public variety (2000's) | V | N |
| Franklin |  | PI 548563 | Public variety (1970's) | IV | N |
| Freeborn |  | PI 592389 | Public variety (1990's) | I | N |
| Freedom |  | PI 636463 | Public variety (2000's) | V | N |
| Fremont |  | PI 548564 | Public variety (1980's) | III | N |
| G00-3234 |  |  | PI 416937-derived lines | VIII | N |
| G00-3322 |  |  | PI 416937-derived lines | VII | N |
| G00-3364 |  |  | PI 416937-derived lines | VIII | N |
| G07-1185RR |  |  | PI 416937-derived lines | VIII | N |
| G07-1363RR |  |  | PI 416937-derived lines | VI | N |
| G07-1450RR |  |  | PI 416937-derived lines | VI | N |
| G07-1460RR |  |  | PI 416937-derived lines | VI | N |
| G07-1463RR |  |  | PI 416937-derived lines | VIII | N |
| G10-3913R2 |  |  | PI 416937-derived lines | VIII | N |
| G10-3954R2 |  |  | PI 416937-derived lines | VII | N |
| G10-3968R2 |  |  | PI 416937-derived lines | VII | N |
| G10PR-224R2 |  |  | PI 416937-derived lines | VII | N |
| G10PR-56264R2 |  |  | PI 416937-derived lines | VIII | N |
| G10PR-56288R2 |  |  | PI 416937-derived lines | VIII | N |
| G10PR-56330R2 |  |  | PI 416937-derived lines | VII | N |
| G10PR-56351R2 |  |  | PI 416937-derived lines | VIII | N |
| G10PR-56406R2 |  |  | PI 416937-derived lines | VIII | N |
| G10PR-56444R2 |  |  | PI 416937-derived lines | VIII | N |
| G11PR-266R2 |  |  | PI 416937-derived lines | VIII | N |
| G11PR-407R2 |  |  | PI 416937-derived lines | VIII | N |
| G11PR-418R2 |  |  | PI 416937-derived lines | VII | N |
| G11PR-56151R2 |  |  | PI 416937-derived lines | VIII | N |
| G11PR-56158R2 |  |  | PI 416937-derived lines | VII | N |
| G11PR-56183R2 |  |  | PI 416937-derived lines | VII | N |
| Gasoy 17 |  | PI 553046 | Public variety (1970's) | VII | N |
| General |  | PI 593463 | Public variety (1970's) | III | N |
| Glacier |  | PI 592523 | Public variety (1990's) | 0 | N |
| Glenwood |  | PI 513382 | Public variety (1980's) | 0 | N |
| Gnome |  | PI 548565 | Public variety (1980's) | II | N |
| Gnome 85 |  | PI 543857 | Public variety (1990's) | II | N |
| Govan |  | PI 548979 | Public variety (1970's) | VII | N |
| GR8836 |  | PI 534647 | Public variety (1980's) | III | N |
| GR8936 |  | PI 534648 | Public variety (1980's) | III | N |
| Grande |  | PI 548567 | Public variety (1970's) | 0 | N |
| Granite |  | PI 592524 | Public variety (1990's) | I | N |
| Grant |  | PI 548568 | Public variety (1950's) | 0 | N |
| Greencastle |  | PI 655521 | Public variety (2000's) | VI | N |
| Gregg |  | PI 510675 | Public variety (1980's) | VII | N |
| H7190 |  | PI 542972 | Private variety (1990's) | VII | N |
| H9190 |  | PI 556805 | Private variety (1980's) | IX | N |
| Haberlandt |  | PI 548456 | N.A. soybean ancestor | VI | N |
| Hack |  | PI 548569 | Public variety (1980's) | II | N |
| Hagood |  | PI 555453 | Select southern lines | VII | N |
| Hamilton |  | PI 540555 | Public variety (1980's) | IV | N |
| Hampton |  | PI 614156 | Private variety (1960's) | VIII | N |
| Harbar |  | PI 561702 | Public variety (1990's) | VI | N |
| Harcor |  | PI 548570 | Public variety (1970's) | II | N |
| Hardee |  | PI 548666 | Public variety (1960's) | VIII | N |
| Hardin |  | PI 548526 | Public variety (1980's) | I | N |
| Hardome |  | PI 279648 | Public variety (1950's) | 0 | N |
| Hark |  | PI 548551 | Public variety (1960's) | I | N |
| Harlon |  | PI 548571 | Public variety (1970's) | I | N |
| Harly |  | PI 548572 | Public variety (1940's) | I | N |
| Haroson |  | PI 548641 | Public variety (1980's) | I | N |
| Harosoy |  | PI 548573 | Public variety (1950's) | II | N |
| Harosoy 63 |  | PI 548575 | Public variety (1960's) | II | N |
| Harovinton |  | PI 572243 | Public variety (1980's) | I | N |
| Harper |  | PI 548558 | Public variety (1980's) | III | N |
| Harper 87 |  | PI 518667 | Public variety (1980's) | III | N |
| Harwood |  | PI 548576 | Public variety (1970's) | II | N |
| Haskell |  | PI 572238 | Select southern lines | VII | N |
| Hawkeye |  | PI 548577 | Public variety (1940's) | II | N |
| Hawkeye 63 |  | PI 548578 | Public variety (1960's) | II | N |
| Hayes |  | PI 542709 | Public variety (1980's) | III | N |
| Hendricks |  | PI 583365 | Public variety (1990's) | 0 | N |
| Henry |  | PI 548579 | Public variety (1960's) | II | N |
| HF93-035 |  | PI 612932 | Public variety (2000's) | III | N |
| HF93-083 |  | PI 612931 | Public variety (2000's) | II | N |
| Hobbit |  | PI 540551 | Public variety (1980's) | III | N |
| Hobbit 87 |  | PI 546373 | Public variety (1980's) | III | N |
| Hodgson |  | PI 548561 | Public variety (1970's) | I | N |
| Hodgson 78 |  | PI 548581 | Public variety (1970's) | I | N |
| Holladay |  | PI 572239 | Select southern lines | V | N |
| Hood |  | PI 548980 | Public variety (1950's) | VI | N |
| Hood 75 |  | PI 559371 | Public variety (1970's) | VI | N |
| Howard |  | PI 548971 | Public variety (1990's) | VII | N |
| Hoyt |  | PI 540552 | Public variety (1980's) | II | N |
| HP-963 |  | PI 548678 | Private variety (1960's) | IV | N |
| HP201 |  | PI 539862 | Public variety (1990's) | I | N |
| HP202 |  | PI 539863 | Public variety (1990's) | I | N |
| HP203 |  | PI 539864 | Public variety (1990's) | I | N |
| HP204 |  | PI 539865 | Public variety (1990's) | I | N |
| HS93-4118 |  | PI 614155 | Public variety (2000's) | IV | N |
| Hutcheson |  | PI 518664 | Select southern lines | V | N |
| Hutton |  | PI 548662 | Public variety (1970's) | VIII | N |
| IL1 |  | PI 542045 | Public variety (1980's) | II | N |
| IL2 |  | PI 542046 | Public variety (1980's) | III | N |
| Illini |  | PI 548348 | N.A. soybean ancestor | III | N |
| Improved Pelican |  | PI 548461 | N.A. soybean ancestor | VIII | N |
| Ina |  | PI 606749 | Public variety (1990's) | IV | N |
| Iroquois |  | PI 593259 | Public variety (1990's) | III | N |
| Jack |  | PI 540556 | Public variety (1980's) | II | N |
| Jackson |  | PI 548657 | N.A. soybean ancestor | VII | N |
| Jeff |  | PI 553040 | Public variety (1980's) | VI | N |
| Jim |  | PI 602897 | Public variety (1990's) | 0 | N |
| Jogun |  | PI 548352 | N.A. soybean ancestor | III | N |
| Johnston |  | PI 508267 | Select southern lines | VIII | N |
| Jupiter |  | PI 548972 | Public variety (1970's) | IX | N |
| Jupiter-R |  | PI 548973 | Public variety (1980's) | IX | N |
| Kahala |  | PI 355067 | Public variety (1960's) | IV | N |
| Kaikoo |  | PI 355068 | Public variety (1960's) | IV | N |
| Kailua |  | PI 355069 | Public variety (1960's) | IV | N |
| Kanrich |  | PI 548552 | Public variety (1950's) | III | N |
| Kanro |  | PI 548356 | N.A. soybean ancestor | II | N |
| Kasota |  | PI 546038 | Public variety (1990's) | I | N |
| Kato |  | PI 542042 | Public variety (1980's) | I | N |
| Keller |  | PI 548583 | Public variety (1980's) | II | N |
| Kent |  | PI 548586 | Public variety (1960's) | IV | N |
| Kenwood |  | PI 537094 | Public variety (1980's) | II | N |
| Kershaw |  | PI 548985 | Public variety (1980's) | VI | N |
| Kim |  | PI 548587 | Public variety (1950's) | III | N |
| Kino |  | PI 567791 | Public variety (1960's) | VI | N |
| Kirby |  | PI 548665 | Public variety (1980's) | VIII | N |
| Korean |  | PI 548360 | N.A. soybean ancestor | II | N |
| Kottman |  | PI 612594 | Public variety (2000's) | III | N |
| KS3494 |  | PI 586980 | Public variety (1990's) | III | N |
| KS4694 |  | PI 586981 | Public variety (1990's) | IV | N |
| KS4895 |  | PI 595081 | Public variety (1990's) | IV | N |
| KS5292 |  | PI 559934 | Public variety (1990's) | V | N |
| Kuell |  | PI 608033 | Public variety (1990's) | VIII | N |
| Kunitz |  | PI 542044 | Public variety (1980's) | III | N |
| Lakota |  | PI 548588 | Public variety (1980's) | I | N |
| Lamar |  | PI 533604 | Public variety (1980's) | VI | N |
| Lambert |  | PI 562373 | Public variety (1990's) | 0 | N |
| LaMoure |  | PI 634813 | Public variety (2000's) | 0 | N |
| Lancaster |  | PI 561860 | Public variety (1990's) | III | N |
| Lawrence |  | PI 518673 | Public variety (1980's) | IV | N |
| LD00-3309 |  | PI 639740 | Public variety (2000's) | IV | N |
| Lee 68 |  | PI 559369 | Public variety (1960's) | VI | N |
| Lee 74 |  | PI 548658 | Public variety (1970's) | VI | N |
| Leflore |  | PI 548981 | Public variety (1980's) | VI | N |
| Leslie |  | PI 557011 | Public variety (1990's) | I | N |
| Lincoln |  | PI 548362 | N.A. soybean ancestor | III | N |
| Lindarin |  | PI 548589 | Public variety (1950's) | II | N |
| Lindarin 63 |  | PI 548590 | Public variety (1960's) | II | N |
| Linford |  | PI 542043 | Public variety (1980's) | III | N |
| Lloyd |  | PI 533602 | Public variety (1980's) | VI | N |
| LN83-2356 |  | PI 533654 | Public variety (1980's) | IV | N |
| LN89-3264 |  | PI 597383 | Public variety (1990's) | II | N |
| LN89-3615 |  | PI 597384 | Public variety (1990's) | IV | N |
| LN90-4524 |  | PI 593257 | Public variety (1990's) | III | N |
| LN92-11008 |  | PI 597385 | Public variety (1990's) | III | N |
| LN92-7369 |  | PI 607385 | Public variety (1990's) | II | N |
| LN97-15076 |  | PI 633983 | Public variety (2000's) | IV | N |
| Loda |  | PI 614088 | Public variety (2000's) | II | N |
| Logan |  | PI 548591 | Public variety (1980's) | III | N |
| Lonoke |  | PI 633609 | Public variety (2000's) | V | N |
| LS201 |  | PI 539866 | Public variety (1990's) | II | N |
| LS301 |  | PI 539867 | Public variety (1990's) | III | N |
| LS90-1920 |  | PI 604100 | Public variety (1990's) | IV | N |
| LS92-1800 |  | PI 607380 | Public variety (1990's) | IV | N |
| LS93-0375 |  | PI 620883 | Public variety (2000's) | IV | N |
| LS94-3207 |  | PI 634335 | Public variety (2000's) | IV | N |
| Lyon |  | PI 576857 | Public variety (1990's) | VI | N |
| Mack |  | PI 559370 | Public variety (1970's) | V | N |
| Macon |  | PI 593258 | Public variety (1990's) | III | N |
| Madison |  | PI 548580 | Public variety (1960's) | II | N |
| Magna |  | PI 548553 | Public variety (1960's) | II | N |
| Majos |  | PI 548697 | Private variety (1990's) | VIII | N |
| Mandarin (Ottawa) |  | PI 548379 | N.A. soybean ancestor | 0 | N |
| Manitoba Brown |  | PI 548382 | N.A. soybean ancestor | 00 | N |
| Maple Amber |  | PI 548592 | Public variety (1980's) | 0 | N |
| Maple Arrow |  | PI 548593 | Public variety (1970's) | 0 | N |
| Maple Donovan |  | PI 548642 | Public variety (1980's) | 0 | N |
| Maple Glen |  | PI 548643 | Public variety (1980's) | 0 | N |
| Maple Isle |  | PI 548595 | Public variety (1980's) | 0 | N |
| Maple Presto |  | PI 548594 | Public variety (1970's) | 0 | N |
| Maple Ridge |  | PI 548596 | Public variety (1980's) | 0 | N |
| Marcus |  | PI 537095 | Public variety (1980's) | II | N |
| Marion |  | PI 548537 | Public variety (1970's) | II | N |
| Marshall |  | PI 548693 | Private variety (1980's) | II | N |
| Maverick |  | PI 598124 | Public variety (1990's) | III | N |
| Maxcy |  | PI 568236 | Select southern lines | VIII | N |
| McCall |  | PI 548582 | Public variety (1970's) | 0 | N |
| Mead |  | PI 548597 | Public variety (1980's) | III | N |
| Mercury |  | PI 583835 | Public variety (1990's) | III | N |
| Merit |  | PI 548545 | Public variety (1950's) | 0 | N |
| Merrimax |  | PI 548651 | Public variety (1980's) | 0 | N |
| Miami |  | PI 548584 | Public variety (1980's) | II | N |
| Miles |  | PI 548598 | Public variety (1970's) | IV | N |
| Minnatto |  | PI 537096 | Public variety (1980's) | 0 | N |
| MN0201 |  | PI 629004 | Public variety (2000's) | 0 | N |
| MN0301 |  | PI 602594 | Public variety (1990's) | 0 | N |
| MN0302 |  | PI 629005 | Public variety (2000's) | 0 | N |
| MN0901 |  | PI 612764 | Public variety (2000's) | 0 | N |
| MN1301 |  | PI 602593 | Public variety (1990's) | I | N |
| MN1302 |  | PI 616498 | Public variety (2000's) | I | N |
| MN1401 |  | PI 608726 | Public variety (2000's) | I | N |
| MN1801 |  | PI 612763 | Public variety (2000's) | I | N |
| Mokapu Summer |  | PI 355070 | Public variety (1960's) | IV | N |
| Monroe |  | PI 548599 | Public variety (1940's) | I | N |
| Moon Cake |  | PI 632905 | Public variety (2000's) | V | N |
| Morgan |  | PI 510670 | Public variety (1980's) | IV | N |
| Morsoy |  | PI 548600 | Public variety (1970's) | 0 | N |
| Motte |  | PI 603953 | Public variety (1990's) | VIII | N |
| Mukden |  | PI 548391 | N.A. soybean ancestor | II | N |
| Musen |  | PI 599333 | Public variety (1990's) | VI | N |
| Mustang |  | PI 595363 | Public variety (1990's) | IV | N |
| N09-13128 |  |  | PI 416937-derived lines | VII | N |
| N09-13317 |  |  | PI 416937-derived lines | VIII | N |
| N09-13663 |  |  | PI 416937-derived lines | VIII | N |
| N09-13671 |  |  | PI 416937-derived lines | VII | N |
| N09-13690 |  |  | PI 416937-derived lines | VII | N |
| N6201 |  | PI 619615 | Public variety (2000's) | VI | N |
| N7101 |  | PI 619616 | Public variety (2000's) | VII | N |
| N7102 |  | PI 619617 | Public variety (2000's) | VII | N |
| N7103 |  | PI 615695 | Public variety (2000's) | VII | N |
| N8101 |  | PI 654355 | Public variety (2000's) | VIII | N |
| Nannonatto |  | PI 631438 | Public variety (2000's) | 0 | N |
| Narow |  | PI 553052 | Public variety (1980's) | V | N |
| Nathan |  | PI 564849 | Public variety (1980's) | V | N |
| Nattawa |  | PI 548649 | Public variety (1980's) | 0 | N |
| Nattosan |  | PI 548650 | Public variety (1980's) | 0 | N |
| NC-Raleigh |  | PI 641156 | Select southern lines | VII | N |
| NCC06-1090 |  |  | PI 416937-derived lines | VI | N |
| NCC06-899 |  |  | PI 416937-derived lines | VII | N |
| NE1900 |  | PI 614833 | Public variety (2000's) | I | N |
| NE2701 |  | PI 634827 | Public variety (2000's) | II | N |
| NE3297 |  | PI 610670 | Public variety (1990's) | III | N |
| NE3399 |  | PI 610671 | Public variety (1990's) | III | N |
| NE3400 |  | PI 614832 | Public variety (2000's) | III | N |
| Nebsoy |  | PI 548566 | Public variety (1970's) | II | N |
| Nemaha |  | PI 595754 | Public variety (1990's) | III | N |
| Newton |  | PI 543855 | Public variety (1990's) | II | N |
| Nile |  | PI 572240 | Public variety (1990's) | IV | N |
| Nitrasoy |  | PI 642732 | Public variety (2000's) | VI | N |
| No.94 |  | PI 071506 | N.A. soybean ancestor | IV | N |
| Norchief |  | PI 548601 | Public variety (1950's) | 0 | N |
| Norman |  | PI 548535 | Public variety (1960's) | 0 | N |
| Nornatto |  | PI 631437 | Public variety (2000's) | 0 | N |
| Norpro |  | PI 603900 | Public variety (1990's) | 0 | N |
| OAC Aries |  | PI 548637 | Public variety (1980's) | 0 | N |
| OAC Dorado |  | PI 567782 | Public variety (1980's) | I | N |
| OAC Eclipse |  | PI 567783 | Public variety (1980's) | 0 | N |
| OAC Frontier |  | PI 567784 | Public variety (1980's) | 0 | N |
| OAC Libra |  | PI 548638 | Public variety (1980's) | 0 | N |
| OAC Musca |  | PI 548644 | Public variety (1980's) | 0 | N |
| OAC Pisces |  | PI 548639 | Public variety (1980's) | 0 | N |
| OAC Scorpio |  | PI 548640 | Public variety (1980's) | 0 | N |
| OAC Shire |  | PI 567785 | Public variety (1990's) | I | N |
| OAC Talbot |  | PI 567786 | Public variety (1990's) | II | N |
| OAC Vision |  | PI 567787 | Public variety (1990's) | 0 | N |
| Oakland |  | PI 548543 | Public variety (1970's) | III | N |
| Odell |  | PI 595753 | Public variety (1990's) | III | N |
| Ogden |  | PI 548477 | N.A. soybean ancestor | VI | N |
| Ohio FG1 |  | PI 584469 | Public variety (1990's) | III | N |
| Ohio FG2 |  | PI 584470 | Public variety (1990's) | III | N |
| OHIO FG3 |  | PI 629008 | Public variety (2000's) | II | N |
| Ohio FG5 |  | PI 642768 | Public variety (2000's) | III | N |
| Oksoy |  | PI 548602 | Public variety (1970's) | IV | N |
| Olympus |  | PI 602060 | Public variety (1990's) | II | N |
| Omaha |  | PI 597382 | Public variety (1990's) | IV | N |
| Osage |  | PI 648270 | Public variety (2000's) | V | N |
| Ottawa |  | PI 548673 | Private variety (1960's) | I | N |
| Owens |  | PI 633567 | Public variety (2000's) | V | N |
| Ozark |  | PI 633970 | Public variety (2000's) | V | N |
| Ozzie |  | PI 542404 | Public variety (1980's) | 0 | N |
| Pace |  | PI 602496 | Public variety (1990's) | V | N |
| Padre |  | PI 518665 | Public variety (1980's) | VII | N |
| Palmetto |  | PI 548480 | N.A. soybean ancestor | VII | N |
| Pana |  | PI 597387 | Public variety (1990's) | III | N |
| Parker |  | PI 562374 | Public variety (1990's) | I | N |
| Patoka |  | PI 548400 | N.A. soybean ancestor | IV | N |
| Pearl |  | PI 583367 | Public variety (1990's) | VII | N |
| Peking |  | PI 548402 | N.A. soybean ancestor | IV | N |
| Pella |  | PI 548523 | Public variety (1970's) | III | N |
| Pella 86 |  | PI 509044 | Public variety (1980's) | III | N |
| Pembina |  | PI 638510 | Public variety (2000's) | 0 | N |
| Pennyrile |  | PI 515961 | Public variety (1980's) | IV | N |
| Perrin |  | PI 536637 | Public variety (1980's) | VIII | N |
| Perry |  | PI 548603 | N.A. soybean ancestor | IV | N |
| Pershing |  | PI 548604 | Public variety (1980's) | IV | N |
| Peterson Jade |  | PI 548694 | Private variety (1970's) | II | N |
| Pharaoh |  | PI 548645 | Public variety (1980's) | IV | N |
| PI 054610 |  | PI 054610 | N.A. soybean ancestor | VI | N |
| PI 080837 |  | PI 080837 | N.A. soybean ancestor | IV | N |
| PI 081041 |  | PI 081041 | N.A. soybean ancestor | III | N |
| PI 088788 |  | PI 088788 | N.A. soybean ancestor | III | N |
| PI 221717 |  | PI 221717 | Select southern lines | VI | N |
| PI 416937 |  | PI 416937 | PI 416937 | VI | Y |
| Piatt |  | PI 574534 | Public variety (1990's) | III | N |
| Pickett |  | PI 548988 | Public variety (1960's) | VI | N |
| Pickett 71 |  | PI 548982 | Public variety (1970's) | VI | N |
| Pixie |  | PI 543856 | Public variety (1980's) | IV | N |
| Platte |  | PI 548605 | Public variety (1980's) | II | N |
| Pomona |  | PI 548606 | Public variety (1970's) | IV | N |
| Portage |  | PI 548607 | Public variety (1960's) | 0 | N |
| Preston |  | PI 548520 | Public variety (1980's) | II | N |
| Pridesoy 57 |  | PI 548680 | Private variety (1950's) | I | N |
| Pritchard |  | PI 612157 | Select southern lines | VIII | N |
| Prize |  | PI 548554 | Public variety (1960's) | II | N |
| Probst |  | PI 587185 | Public variety (1990's) | III | N |
| Prohio |  | PI 643146 | Public variety (2000's) | IV | N |
| Prolina |  | PI 597389 | Public variety (1990's) | VI | N |
| ProSoy |  | PI 638511 | Public variety (2000's) | 0 | N |
| Protana |  | PI 548528 | Public variety (1960's) | II | N |
| Proto |  | PI 542769 | Public variety (1980's) | 0 | N |
| Provar |  | PI 548608 | Public variety (1960's) | II | N |
| Pyramid |  | PI 512039 | Public variety (1980's) | IV | N |
| Ralsoy |  | PI 548484 | N.A. soybean ancestor | VI | N |
| Rampage |  | PI 548609 | Public variety (1960's) | I | N |
| Randolph |  | PI 633424 | Public variety (2000's) | VI | N |
| Ransom |  | PI 548989 | Select southern lines | VII | N |
| RCAT Alliance |  | PI 548646 | Public variety (1980's) | II | N |
| RCAT Angora |  | PI 572242 | Public variety (1990's) | II | N |
| RCAT Persian |  | PI 548647 | Public variety (1980's) | I | N |
| Regal |  | PI 548636 | Public variety (1980's) | IV | N |
| Rend |  | PI 606748 | Public variety (1990's) | IV | N |
| Renville |  | PI 548611 | Public variety (1950's) | I | N |
| Resnik |  | PI 534645 | Public variety (1980's) | III | N |
| Rhodes |  | PI 561400 | Public variety (1990's) | V | N |
| Richland |  | PI 548406 | N.A. soybean ancestor | II | N |
| Ripley |  | PI 536636 | Public variety (1980's) | IV | N |
| Roanoke |  | PI 548485 | N.A. soybean ancestor | VII | N |
| Roe |  | PI 548675 | Private variety (1950's) | IV | N |
| Ross |  | PI 548612 | Public variety (1960's) | III | N |
| S-100 |  | PI 548488 | N.A. soybean ancestor | V | N |
| S1492 |  | PI 548690 | Private variety (1970's) | II | N |
| S99-3181 |  | PI 635039 | Public variety (2000's) | V | N |
| Saline |  | PI 578057 | Public variety (1990's) | III | N |
| Sandusky |  | PI 576145 | Public variety (1990's) | II | N |
| Santee |  | PI 617041 | Public variety (2000's) | VII | N |
| Sargent |  | PI 615585 | Public variety (2000's) | 0 | N |
| Saturn |  | PI 583837 | Public variety (1990's) | III | N |
| Savoy |  | PI 597381 | Public variety (1990's) | II | N |
| SC07-108 RR |  |  | PI 416937-derived lines | VII | N |
| SC09-039 RR |  |  | PI 416937-derived lines | VII | N |
| SC09-052 RR |  |  | PI 416937-derived lines | VII | N |
| SC09-092 RR |  |  | PI 416937-derived lines | VIII | N |
| SC09-102 RR |  |  | PI 416937-derived lines | VIII | N |
| SC09-142 RR |  |  | PI 416937-derived lines | VII | N |
| Scott |  | PI 548613 | Public variety (1950's) | IV | N |
| Semmes |  | PI 548661 | Public variety (1960's) | VII | N |
| Sharkey |  | PI 515960 | Public variety (1980's) | VI | N |
| Shelby |  | PI 548574 | Public variety (1950's) | III | N |
| Sherman |  | PI 548614 | Public variety (1980's) | III | N |
| Shore |  | PI 553049 | Public variety (1970's) | V | N |
| Sibley |  | PI 508084 | Public variety (1980's) | I | N |
| Simpson |  | PI 548615 | Public variety (1980's) | 0 | N |
| Sloan |  | PI 548616 | Public variety (1970's) | II | N |
| Sohoma |  | PI 548990 | Public variety (1970's) | VI | N |
| Soyola |  | PI 614702 | Public variety (2000's) | VI | N |
| Sparks |  | PI 548619 | Public variety (1980's) | IV | N |
| Spencer |  | PI 525454 | Public variety (1980's) | IV | N |
| Sprite |  | PI 536635 | Public variety (1980's) | III | N |
| Sprite 87 |  | PI 546374 | Public variety (1980's) | III | N |
| Spry |  | PI 553051 | Public variety (1990's) | IV | N |
| SRF 100 |  | PI 548681 | Private variety (1970's) | I | N |
| SRF 150 |  | PI 548683 | Private variety (1970's) | I | N |
| SRF 300 |  | PI 548686 | Private variety (1960's) | III | N |
| SRF 307B |  | PI 548684 | Private variety (1970's) | III | N |
| SRF 400 |  | PI 548682 | Private variety (1970's) | IV | N |
| SRF 450 |  | PI 548685 | Private variety (1970's) | IV | N |
| SS201 |  | PI 539860 | Public variety (1980's) | II | N |
| SS202 |  | PI 539861 | Public variety (1980's) | II | N |
| Stafford |  | PI 508269 | Public variety (1980's) | IV | N |
| Stalwart |  | PI 632402 | Public variety (2000's) | III | N |
| Steele |  | PI 548620 | Public variety (1970's) | I | N |
| Stout |  | PI 614807 | Public variety (2000's) | III | N |
| Strain No.18 |  | PI 180501 | N.A. soybean ancestor | 0 | N |
| Stressland |  | PI 593654 | Public variety (1990's) | IV | N |
| Stride |  | PI 599299 | Public variety (1990's) | I | N |
| Strong |  | PI 614808 | Public variety (2000's) | IV | N |
| Sturdy |  | PI 542768 | Public variety (1980's) | II | N |
| Surge |  | PI 599300 | Public variety (1990's) | 0 | N |
| Swift |  | PI 548500 | Public variety (1970's) | 0 | N |
| Tara |  | PI 632418 | Public variety (2000's) | V | N |
| Thorne |  | PI 564718 | Public variety (1990's) | III | N |
| Tiffin |  | PI 612930 | Public variety (2000's) | II | N |
| Titan |  | PI 608438 | Public variety (1990's) | I | N |
| TN 4-86 |  | PI 518668 | Public variety (1980's) | IV | N |
| TN 4-94 |  | PI 598222 | Public variety (1990's) | IV | N |
| TN 5-85 |  | PI 548991 | Public variety (1980's) | V | N |
| TN 5-95 |  | PI 598358 | Public variety (1990's) | V | N |
| TN 6-90 |  | PI 564999 | Public variety (1990's) | VI | N |
| TN03-349 |  |  | PI 416937-derived lines | V | N |
| TN93-99 |  | PI 631122 | Select southern lines | V | N |
| Toano |  | PI 508268 | Public variety (1980's) | V | N |
| Tokyo |  | PI 548493 | N.A. soybean ancestor | VII | N |
| Toyopro |  | PI 592560 | Public variety (1990's) | 0 | N |
| Tracy |  | PI 548983 | Public variety (1970's) | VI | N |
| Tracy-M |  | PI 548984 | Public variety (1970's) | VI | N |
| Traill |  | PI 596541 | Public variety (1990's) | 0 | N |
| Traverse |  | PI 548621 | Public variety (1960's) | 0 | N |
| Troll |  | PI 614806 | Public variety (1990's) | IV | N |
| Tyrone |  | PI 601984 | Public variety (1990's) | VII | N |
| UA 4805 |  | PI 639187 | Public variety (2000's) | IV | N |
| UM3 |  | PI 607835 | Public variety (2000's) | 0 | N |
| Union |  | PI 548622 | Public variety (1970's) | IV | N |
| Vance |  | PI 553048 | Public variety (1980's) | V | N |
| Vansoy |  | PI 548623 | Public variety (1970's) | 0 | N |
| Verde |  | PI 548624 | Public variety (1960's) | III | N |
| Vernal |  | PI 564261 | Public variety (1990's) | VI | N |
| Vertex |  | PI 576146 | Public variety (1990's) | II | N |
| Vickery |  | PI 548617 | Public variety (1970's) | II | N |
| Vinton |  | PI 548618 | Public variety (1970's) | I | N |
| Vinton 81 |  | PI 548625 | Public variety (1980's) | I | N |
| Wabash |  | PI 548626 | Public variety (1940's) | IV | N |
| Walsh |  | PI 615586 | Public variety (2000's) | 0 | N |
| Walters |  | PI 544354 | Public variety (1990's) | V | N |
| Ware |  | PI 548627 | Public variety (1970's) | IV | N |
| Washita |  | PI 618809 | Public variety (2000's) | V | N |
| Wayne |  | PI 548628 | Public variety (1960's) | III | N |
| Weber |  | PI 548524 | Public variety (1970's) | I | N |
| Weber 84 |  | PI 548629 | Public variety (1980's) | I | N |
| Wells |  | PI 548630 | Public variety (1970's) | II | N |
| Wells II |  | PI 548513 | Public variety (1970's) | II | N |
| Wilkin |  | PI 548501 | Public variety (1970's) | 0 | N |
| Will |  | PI 518672 | Public variety (1970's) | III | N |
| Williams |  | PI 548631 | Public variety (1970's) | III | N |
| Williams 79 |  | PI 518670 | Public variety (1970's) | III | N |
| Williams 82 |  | PI 518671 | Public variety (1980's) | III | N |
| Winchester |  | PI 548585 | Public variety (1980's) | III | N |
| Wirth |  | PI 548610 | Public variety (1960's) | I | N |
| Woodworth |  | PI 548632 | Public variety (1970's) | III | N |
| Wye |  | PI 548633 | Public variety (1970's) | IV | N |
| Yale |  | PI 584441 | Public variety (1990's) | III | N |
| Yelnanda |  | PI 548698 | Private variety (1990's) | VIII | N |
| York |  | PI 553038 | Public variety (1960's) | V | N |
| Zane |  | PI 548634 | Public variety (1980's) | III | N |
