# Supplementary material for: Characterizing the impact of an exotic soybean line on elite cultivar development
Source: PLoS One. 2020 Jul 10;15(7):e0235434. doi: 10.1371/journal.pone.0235434 (PMC7351202; doi:10.1371/journal.pone.0235434)
Supplement: S3 Table — (DOCX) [file pone.0235434.s012.docx]

S3 Table. List of genotypes included in S1 Fig.

| Name | Alias | PI number | PI 416937 derived line (Yes/No) | Included in PI 416937 pedigree analysis (Yes/No) | Number identifier |
| --- | --- | --- | --- | --- | --- |
| G10-3833RR |  |  | YES | YES | 1 |
| G10-3896RR |  |  | YES | YES | 2 |
| G08-3279RR |  |  | YES | YES | 3 |
| G08-3282RR |  |  | YES | YES | 4 |
| G08-2869RR |  |  | YES | YES | 5 |
| G07-3557RR |  |  | YES | YES | 6 |
| Woodruff | G00-3209 |  | YES | YES | 7 |
| N05-7462 |  |  | YES | YES | 8 |
| N05-7452 |  |  | YES | YES | 9 |
| G03-825RR |  |  | NO | YES | 10 |
| G03-952RR |  |  | NO | YES | 11 |
| G03-364RR |  |  | NO | YES | 12 |
| P97M50 |  |  | NO | YES | 13 |
| N04-8947 |  |  | YES | YES | 14 |
| N01-11424 |  |  | YES | YES | 15 |
| N01-11491 |  |  | YES | YES | 16 |
| N01-11118 |  |  | YES | YES | 17 |
| N01-11136 |  |  | YES | YES | 18 |
| N05-316 |  |  | YES | YES | 19 |
| N05-7260 |  |  | YES | YES | 20 |
| N05-7229 |  |  | YES | YES | 21 |
| N06-7564 |  |  | YES | YES | 22 |
| N06-7535 |  |  | YES | YES | 23 |
| N01-11884 |  |  | YES | YES | 24 |
| N01-11771 |  |  | YES | YES | 25 |
| N01-11777 |  |  | YES | YES | 26 |
| N01-11832 |  |  | YES | YES | 27 |
| N01-11791 |  |  | YES | YES | 28 |
| N06-7187 |  |  | YES | YES | 29 |
| TCWN23-507 |  |  | YES | YES | 30 |
| N99-8141 |  |  | YES | YES | 31 |
| N05-7281 |  |  | YES | YES | 32 |
| N07-14221 |  |  | YES | YES | 33 |
| G00-3213 |  |  | YES | YES | 34 |
| N07-14182 |  |  | YES | YES | 35 |
| G00-3083 |  |  | YES | YES | 36 |
| N05-7375 |  |  | YES | YES | 37 |
| N05-7380 |  |  | YES | YES | 38 |
| N05-7396 |  |  | YES | YES | 39 |
| N05-7353 |  |  | YES | YES | 40 |
| N8002 | N05-7432 | PI 676972 | YES | YES | 41 |
| N06-7280 |  |  | YES | YES | 42 |
| N09-12441 |  |  | YES | YES | 43 |
| N09-12455 |  |  | YES | YES | 44 |
| N09-12414 |  |  | YES | YES | 45 |
| 5601T |  | PI 630984 | NO | YES | 46 |
| N07-15529 |  |  | YES | YES | 47 |
| N07-15546 |  |  | YES | YES | 48 |
| Hartz H7242RR |  |  | NO | YES | 49 |
| G95-346RR |  |  | NO | YES | 50 |
| N96-6894 |  |  | YES | YES | 51 |
| N97-9812 |  |  | YES | YES | 52 |
| NTCPR94-5157 |  |  | NO | YES | 53 |
| N93-1264 |  |  | YES | YES | 54 |
| NC-Roy |  | PI 617045 | NO | YES | 55 |
| N96-6767 |  |  | YES | YES | 56 |
| N96-7031 |  |  | YES | YES | 57 |
| N96-6752 |  |  | YES | YES | 58 |
| N96-6755 |  |  | YES | YES | 59 |
| N96-6751 |  |  | YES | YES | 60 |
| N96-6809 |  |  | YES | YES | 61 |
| N8001 | N97-9612 | PI 647086 | YES | YES | 62 |
| Graham |  | PI 594922 | NO | YES | 63 |
| N98-7265 |  |  | NO | YES | 64 |
| N7002 | N97-9658 | PI 647085 | YES | YES | 65 |
| Boggs |  | PI 602597 | NO | YES | 66 |
| Benning |  | PI 595645 | NO | YES | 67 |
| Misuzu Daizu |  | PI 423912 | NO | YES | 68 |
| G94-3117 |  |  | NO | YES | 69 |
| TN89-39 |  |  | NO | YES | 70 |
| PI 221717 |  | PI 221717 | NO | YES | 71 |
| Resnik RR |  |  | NO | YES | 72 |
| N90-7216 |  |  | NO | YES | 73 |
| N91-7254 |  |  | YES | YES | 74 |
| Holladay |  | PI 572239 | NO | YES | 75 |
| Brim |  | PI 548986 | NO | YES | 76 |
| N90-7241 |  |  | YES | YES | 77 |
| N93-110-6 |  |  | YES | YES | 78 |
| N90-7202 |  |  | YES | YES | 79 |
| N7001 | N90-7199 | PI 615694 | YES | YES | 80 |
| Clifford |  | PI 596414 | NO | YES | 81 |
| Cook |  | PI 553045 | NO | YES | 82 |
| PI 471938 |  | PI 471938 | NO | YES | 83 |
| G81-152 |  |  | NO | YES | 84 |
| Coker 6738 |  |  | NO | YES | 85 |
| Hutcheson |  | PI 518664 | NO | YES | 86 |
| Hagood |  | PI 555453 | NO | YES | 87 |
| TN80-69 |  |  | NO | YES | 88 |
| G86-1434 |  |  | NO | YES | 89 |
| P449 |  |  | NO | YES | 90 |
| Dixie |  | PI 548452 | NO | YES | 91 |
| Resnik |  | PI 534645 | NO | YES | 92 |
| Nanda |  | PI 548474 | NO | YES | 93 |
| Mon40-3-2 |  |  | NO | YES | 94 |
| Johnston |  | PI 508267 | NO | YES | 95 |
| PI 416937 |  | PI 416937 | YES | YES | 96 |
| N77-179 |  |  | NO | YES | 97 |
| N73-1102 |  |  | NO | YES | 98 |
| Young |  | PI 508266 | NO | YES | 99 |
| N77-114 |  |  | NO | YES | 100 |
| V68-1034 |  |  | NO | NO | 101 |
| J74-40 |  |  | NO | NO | 102 |
| Coker 368 |  |  | NO | NO | 103 |
| D74-7741 |  |  | NO | NO | 104 |
| D79-6058 |  |  | NO | NO | 105 |
| Twiggs |  | PI 511813 | NO | NO | 106 |
| PI 37330 |  | PI 37330 | NO | NO | 107 |
| Asgrow A3127 |  | PI 556511 | NO | NO | 108 |
| PI 95727 |  | PI 95727 | NO | NO | 109 |
| Davis |  | PI 553039 | NO | NO | 110 |
| N70-2173 |  |  | NO | NO | 111 |
| Gasoy17 |  |  | NO | NO | 112 |
| Coker 237 |  | PI 556536 | NO | NO | 113 |
| N72-3213 |  |  | NO | NO | 114 |
| N70-1549 |  |  | NO | NO | 115 |
| Braxton |  | PI 548659 | NO | NO | 116 |
| Pixie |  | PI 543856 | NO | NO | 117 |
| Tracy |  | PI 548983 | NO | NO | 118 |
| PI 88788 |  | PI 88788 | NO | NO | 119 |
| D68-18 |  |  | NO | NO | 120 |
| Coker 71-211 |  |  | NO | NO | 121 |
| Essex |  | PI 548667 | NO | NO | 122 |
| Forrest |  | PI 548655 | NO | NO | 123 |
| D70-3001 |  |  | NO | NO | 124 |
| Centennial |  | PI 548975 | NO | NO | 125 |
| Williams 82 |  | PI 518671 | NO | NO | 126 |
| Ransom |  | PI 548989 | NO | NO | 127 |
| Hutton |  | PI 548662 | NO | NO | 128 |
| N63-858 |  |  | NO | NO | 129 |
| D65-6765 |  |  | NO | NO | 130 |
| N64-2451 |  |  | NO | NO | 131 |
| D67-B5 |  |  | NO | NO | 132 |
| Dare |  | PI 548987 | NO | NO | 133 |
| D69-7965 |  |  | NO | NO | 134 |
| Bragg |  | PI 548660 | NO | NO | 135 |
| D61-618 |  |  | NO | NO | 136 |
| PI 71506 |  | PI 71506 | NO | NO | 137 |
| D64-4636 |  |  | NO | NO | 138 |
| Dyer |  | PI 548976 | NO | NO | 139 |
| Pickett 71 |  | PI 548982 | NO | NO | 140 |
| TN81-2 |  |  | NO | NO | 141 |
| Williams |  | PI 548631 | NO | NO | 142 |
| N55-3818 |  |  | NO | NO | 143 |
| N55-5931 |  |  | NO | NO | 144 |
| Hampton |  | PI 614516 | NO | NO | 145 |
| C.N.S-4 |  |  | NO | NO | 146 |
| F55-822 |  |  | NO | NO | 147 |
| N55-3843 |  |  | NO | NO | 148 |
| D59-9289 |  |  | NO | NO | 149 |
| D58-3358 |  |  | NO | NO | 150 |
| D62-7816 |  |  | NO | NO | 151 |
| N55-2908 |  |  | NO | NO | 152 |
| F59-1505 |  |  | NO | NO | 153 |
| D56-1185 |  |  | NO | NO | 154 |
| D60-9647 |  |  | NO | NO | 155 |
| PI 171442 |  | PI 171442 | NO | NO | 156 |
| Hampton 266 |  |  | NO | NO | 157 |
| S5-7075 |  |  | NO | NO | 158 |
| York |  | PI 553038 | NO | NO | 159 |
| D58-3311 |  |  | NO | NO | 160 |
| Hill |  | PI 548654 | NO | NO | 161 |
| Pickett |  |  | NO | NO | 162 |
| Lee74 |  | PI 548658 | NO | NO | 163 |
| R66-1517 |  |  | NO | NO | 164 |
| Kingwa |  | PI 548359 | NO | NO | 165 |
| L57-0034 |  |  | NO | NO | 166 |
| Wayne |  | PI 548628 | NO | NO | 167 |
| N45-1497 |  |  | NO | NO | 168 |
| N45-2994 |  |  | NO | NO | 169 |
| D49-2573 |  |  | NO | NO | 170 |
| N44-92 |  |  | NO | NO | 171 |
| N48-1867 |  |  | NO | NO | 172 |
| D52-810 |  |  | NO | NO | 173 |
| Hood |  | PI 548980 | NO | NO | 174 |
| D51-4877 |  |  | NO | NO | 175 |
| Majos |  | PI 548697 | NO | NO | 176 |
| PI 181537 |  | PI 181537 | NO | NO | 177 |
| Jackson |  | PI 548657 | NO | NO | 178 |
| D49-2491 |  |  | NO | NO | 179 |
| FC31745 |  |  | NO | NO | 180 |
| N48-1248 |  |  | NO | NO | 181 |
| Perry |  | PI 548603 | NO | NO | 182 |
| D49-2510 |  |  | NO | NO | 183 |
| D49-2525 |  |  | NO | NO | 184 |
| D63-215 |  |  | NO | NO | 185 |
| Lee |  | PI 548656 | NO | NO | 186 |
| Dorman |  | PI 548653 | NO | NO | 187 |
| Peking |  | PI 548402 | NO | NO | 188 |
| FC33243 |  |  | NO | NO | 189 |
| Adams |  | PI 548502 | NO | NO | 190 |
| Clark |  | PI 548533 | NO | NO | 191 |
| L49-4091 |  |  | NO | NO | 192 |
| Ralsoy |  | PI 548484 | NO | NO | 193 |
| Roanoke |  | PI 548485 | NO | NO | 194 |
| N45-745 |  |  | NO | NO | 195 |
| D55-4168 |  |  | NO | NO | 196 |
| Yelredo |  | PI 548497 | NO | NO | 197 |
| Haberlandt |  | PI 548456 | NO | NO | 198 |
| Palmetto |  | PI 548480 | NO | NO | 199 |
| Patoka |  | PI 548400 | NO | NO | 200 |
| L37-1355 |  |  | NO | NO | 201 |
| S-100 |  | PI 548488 | NO | NO | 202 |
| Arksoy 2913 |  |  | NO | NO | 203 |
| Dunfield |  | PI 548318 | NO | NO | 204 |
| Richland |  | PI 548406 | NO | NO | 205 |
| Lincoln |  | PI 548362 | NO | NO | 206 |
| Ogden |  | PI 548477 | NO | NO | 207 |
| Nanking |  | PI 71597 | NO | NO | 208 |
| Biloxi |  | PI 548444 | NO | NO | 209 |
| Mammoth Yellow |  | PI 548469 | NO | NO | 210 |
| Laredo |  | PI 548463 | NO | NO | 211 |
| PI 6396 |  | PI 6396 | NO | NO | 212 |
| Volstate |  | PI 548494 | NO | NO | 213 |
| PI 71587 |  | PI 71587 | NO | NO | 214 |
| PI 7218-2 |  | PI 7218-2 | NO | NO | 215 |
| Kuro Daizu |  | PI 81041 | NO | NO | 216 |
| CNS |  | PI 548445 | NO | NO | 217 |
| Arksoy |  | PI 548438 | NO | NO | 218 |
| Illini |  | PI 548348 | NO | NO | 219 |
| PI 36846 |  | PI 36846 | NO | NO | 220 |
| Manchu |  | PI 548365 | NO | NO | 221 |
| Mandarin |  | PI 548378 | NO | NO | 222 |
| PI 23211 |  | PI 23211 | NO | NO | 223 |
| Tokyo |  | PI 548493 | NO | NO | 224 |
| PI 54610 |  | PI 54610 | NO | NO | 225 |
| Clemson |  | PI 548448 | NO | NO | 226 |
| PI 35335 |  | PI 35335 | NO | NO | 227 |
| A.K. |  | PI 548297 | NO | NO | 228 |
| PI 30593 |  | PI 30593 | NO | NO | 229 |
| PI 36653 |  | PI 36653 | NO | NO | 230 |
| PI 8424 |  | PI 8424 | NO | NO | 231 |
| PI 71659 |  | PI 71659 | NO | NO | 232 |
