# Supplementary material for: Characterizing the impact of an exotic soybean line on elite cultivar development
Source: PLoS One. 2020 Jul 10;15(7):e0235434. doi: 10.1371/journal.pone.0235434 (PMC7351202; doi:10.1371/journal.pone.0235434)
Supplement: S4 Table — (DOCX) [file pone.0235434.s013.docx]

S4 Table. Results of whole-genome based RIL pedigree analysis.

| Population | Genomic region | Chr | Positive/negative selection | Physical start position (bp) | Physical stop position (bp) | No. of markers | Trios tested | No. of times inherited | *P*-value |
| --- | --- | --- | --- | --- | --- | --- | --- | --- | --- |
| RIL-1 | RIL-1_01 | 1 | positive | 2102513 | - | 1 | 84 | 62 | 1.4656E-05 |
| RIL-1 | RIL-1_01 | 1 | positive | 2194371 | 2724688 | 4 | 84 | 63 | 4.96802E-06 |
| RIL-1 | RIL-1_01 | 1 | positive | 2829751 | 3387651 | 6 | 84 | 67 | 3.49589E-08 |
| RIL-1 | RIL-1_01 | 1 | positive | 3458900 | - | 1 | 84 | 66 | 1.33287E-07 |
| RIL-1 | RIL-1_01 | 1 | positive | 3530881 | 3853942 | 6 | 84 | 67 | 3.49589E-08 |
| RIL-1 | RIL-1_01 | 1 | positive | 3926357 | 4142416 | 3 | 84 | 68 | 8.54234E-09 |
| RIL-1 | RIL-1_01 | 1 | positive | 4235320 | 4286525 | 2 | 84 | 66 | 1.33287E-07 |
| RIL-1 | RIL-1_01 | 1 | positive | 4387206 | - | 1 | 84 | 68 | 8.54234E-09 |
| RIL-1 | RIL-1_01 | 1 | positive | 4496361 | 4664561 | 3 | 84 | 70 | 4.06796E-10 |
| RIL-1 | RIL-1_02 | 1 | negative | 52989334 | - | 1 | 84 | 9 | 4.30858E-14 |
| RIL-1 | RIL-1_02 | 1 | negative | 53041644 | - | 1 | 56 | 10 | 1.24545E-06 |
| RIL-1 | RIL-1_02 | 1 | negative | 53141084 | 53527381 | 3 | 55 | 10 | 2.05726E-06 |
| RIL-1 | RIL-1_02 | 1 | negative | 53772821 | - | 1 | 54 | 10 | 3.38569E-06 |
| RIL-1 | RIL-1_02 | 1 | negative | 54127023 | - | 1 | 83 | 10 | 5.80066E-13 |
| RIL-1 | RIL-1_02 | 1 | negative | 54163451 | 55194325 | 11 | 84 | 10 | 3.28569E-13 |
| RIL-1 | RIL-1_02 | 1 | negative | 55348314 | 55848750 | 9 | 83 | 10 | 5.80066E-13 |
| RIL-1 | RIL-1_02 | 1 | negative | 55879101 | 56524987 | 6 | 84 | 17 | 3.49589E-08 |
| RIL-1 | RIL-1_03 | 2 | negative | 207504 | - | 1 | 39 | 1 | 1.45519E-10 |
| RIL-1 | RIL-1_03 | 2 | negative | 398523 | 831795 | 7 | 43 | 2 | 2.15323E-10 |
| RIL-1 | RIL-1_03 | 2 | negative | 881270 | 971919 | 2 | 48 | 7 | 6.24041E-07 |
| RIL-1 | RIL-1_03 | 2 | negative | 1033638 | - | 1 | 83 | 10 | 5.80066E-13 |
| RIL-1 | RIL-1_04 | 5 | negative | 35961486 | - | 1 | 84 | 20 | 1.58493E-06 |
| RIL-1 | RIL-1_05 | 6 | negative | 4651121 | 4826081 | 2 | 84 | 22 | 1.4656E-05 |
| RIL-1 | RIL-1_05 | 6 | negative | 4941014 | - | 1 | 84 | 21 | 4.96802E-06 |
| RIL-1 | RIL-1_05 | 6 | negative | 5001043 | - | 1 | 84 | 20 | 1.58493E-06 |
| RIL-1 | RIL-1_05 | 6 | negative | 5064301 | 5140936 | 2 | 84 | 21 | 4.96802E-06 |
| RIL-1 | RIL-1_05 | 6 | negative | 5202219 | 5303094 | 2 | 84 | 22 | 1.4656E-05 |
| RIL-1 | RIL-1_05 | 6 | negative | 5324457 | 6099232 | 10 | 84 | 23 | 4.07713E-05 |
| RIL-1 | RIL-1_06 | 6 | negative | 10809898 | 10919443 | 3 | 84 | 22 | 1.4656E-05 |
| RIL-1 | RIL-1_06 | 6 | negative | 11019800 | 11191735 | 3 | 84 | 23 | 4.07713E-05 |
| RIL-1 | RIL-1_07 | 7 | negative | 36935345 | 37098897 | 4 | 83 | 23 | 5.9736E-05 |
| RIL-1 | RIL-1_07 | 7 | negative | 37166523 | 38295714 | 14 | 84 | 23 | 4.07713E-05 |
| RIL-1 | RIL-1_08 | 7 | negative | 39776460 | 42396323 | 15 | 84 | 23 | 4.07713E-05 |
| RIL-1 | RIL-1_08 | 7 | negative | 42459910 | 42797211 | 5 | 84 | 7 | 5.13756E-16 |
| RIL-1 | RIL-1_09 | 7 | negative | 43012666 | - | 1 | 59 | 7 | 1.35899E-09 |
| RIL-1 | RIL-1_09 | 7 | negative | 43128249 | 43893649 | 9 | 83 | 7 | 9.43055E-16 |
| RIL-1 | RIL-1_09 | 7 | negative | 44307035 | 44567848 | 3 | 83 | 12 | 2.39464E-11 |
| RIL-1 | RIL-1_10 | 10 | negative | 39906756 | - | 1 | 84 | 10 | 3.28569E-13 |
| RIL-1 | RIL-1_11 | 11 | negative | 5800217 | 6242845 | 6 | 84 | 16 | 8.54234E-09 |
| RIL-1 | RIL-1_11 | 11 | negative | 6297386 | 6393348 | 3 | 84 | 14 | 4.06796E-10 |
| RIL-1 | RIL-1_11 | 11 | negative | 6485300 | 6892876 | 5 | 84 | 13 | 7.86392E-11 |
| RIL-1 | RIL-1_11 | 11 | negative | 6911717 | - | 1 | 77 | 13 | 3.02363E-09 |
| RIL-1 | RIL-1_11 | 11 | negative | 6916605 | - | 1 | 72 | 13 | 3.8086E-08 |
| RIL-1 | RIL-1_11 | 11 | negative | 7253927 | - | 1 | 84 | 13 | 7.86392E-11 |
| RIL-1 | RIL-1_11 | 11 | negative | 7368580 | 7854392 | 6 | 84 | 15 | 1.93819E-09 |
| RIL-1 | RIL-1_12 | 11 | negative | 9204696 | 9695622 | 4 | 84 | 17 | 3.50E-08 |
| RIL-1 | RIL-1_12 | 11 | negative | 9949486 | 11031273 | 11 | 84 | 7 | 5.13756E-16 |
| RIL-1 | RIL-1_13 | 11 | positive | 33885696 | - | 1 | 83 | 63 | 2.4307E-06 |
| RIL-1 | RIL-1_14 | 11 | negative | 34272092 | 34656421 | 6 | 84 | 22 | 1.4656E-05 |
| RIL-1 | RIL-1_14 | 11 | negative | 34725337 | - | 1 | 84 | 23 | 4.07713E-05 |
| RIL-1 | RIL-1_15 | 12 | negative | 3550371 | 4830371 | 16 | 83 | 20 | 2.4307E-06 |
| RIL-1 | RIL-1_16 | 12 | negative | 6652775 | 6971475 | 6 | 84 | 20 | 1.58493E-06 |
| RIL-1 | RIL-1_16 | 12 | negative | 6981708 | - | 1 | 84 | 22 | 1.4656E-05 |
| RIL-1 | RIL-1_17 | 18 | positive | 4254294 | 4643663 | 4 | 84 | 63 | 4.96802E-06 |
| RIL-1 | RIL-1_17 | 18 | positive | 4701507 | 4886585 | 4 | 84 | 61 | 4.07713E-05 |
| RIL-1 | RIL-1_18 | 19 | negative | 52957 | 174608 | 3 | 72 | 13 | 3.8086E-08 |
| RIL-1 | RIL-1_18 | 19 | negative | 267267 | - | 1 | 84 | 12 | 1.39323E-11 |
| RIL-1 | RIL-1_18 | 19 | negative | 338015 | 809326 | 7 | 84 | 11 | 2.24909E-12 |
| RIL-2 | RIL-2_01 | 1 | positive | 48171267 | 48216978 | 2 | 84 | 72 | 1.39323E-11 |
| RIL-3 | RIL-3 | NA | NA | NA | NA | NA | NA | NA | NA |
| RIL-4 | RIL-4_01 | 8 | positive | 47400674 | 47655267 | 2 | 84 | 63 | 4.97E-06 |
| RIL-4 | RIL-4_01 | 8 | positive | 47733111 | 47796376 | 2 | 84 | 66 | 1.33E-07 |
| RIL-4 | RIL-4_02 | 18 | positive | 50731387 | - | 1 | 84 | 62 | 1.47E-05 |
| RIL-4 | RIL-4_02 | 18 | positive | 50893479 | - | 1 | 84 | 63 | 4.97E-06 |
| RIL-5 | RIL-5_01 | 6 | negative | 38920680 | 41147967 | 7 | 148 | 14 | 9.19193E-26 |
| RIL-5 | RIL-5_01 | 6 | negative | 41376571 | 43064582 | 4 | 148 | 20 | 1.80414E-20 |
